# Synthesis of 4-substituted azopyridine-functionalized Ni(II)-porphyrins as molecular spin switches

**DOI:** 10.3762/bjoc.16.210

**Published:** 2020-10-21

**Authors:** Jannis Ludwig, Tobias Moje, Fynn Röhricht, Rainer Herges

**Affiliations:** 1 Otto Diels Institute of Organic Chemistry, University of Kiel, Otto-Hahn-Platz 3–4, 24098 Kiel, Germany

**Keywords:** azopyridines, Ni(II)-porphyrins, photoswitch, record player molecules, spin state, spin switch

## Abstract

We present the synthesis and the spin switching efficiencies of Ni(II)-porphyrins substituted with azopyridines as covalently attached photoswitchable ligands. The molecules are designed in such a way that the azopyridines coordinate to the Ni ion if the azo unit is in *cis* configuration. For steric reasons no intramolecular coordination is possible if the azopyridine unit adopts the *trans* configuration. Photoisomerization of the azo unit between *cis* and *trans* is achieved upon irradiation with 505 nm (*trans*→*cis*) and 435 nm (*cis*→*trans*). Concurrently with the isomerization and coordination/decoordination, the spin state of the Ni ion switches between singlet (low-spin) and triplet (high-spin). Previous studies have shown that the spin switching efficiency is strongly dependent on the solvent and on the substituent at the 4-position of the pyridine unit. We now introduced thiol, disulfide, thioethers, nitrile and carboxylic acid groups and investigated their spin switching efficiency.

## Introduction

**Figure 1:**
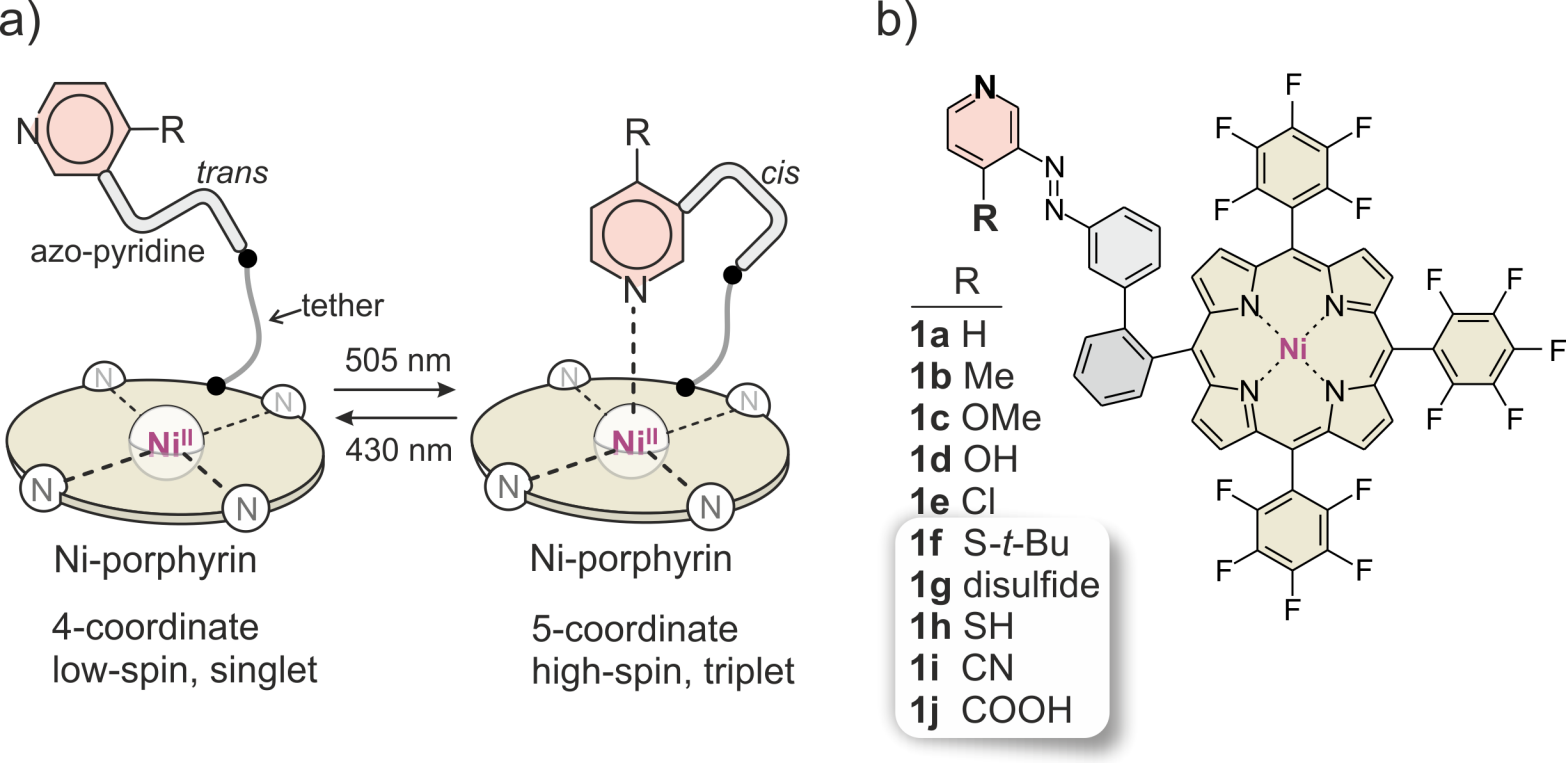
“Record player” approach for molecular spin switching. a) General principle b) Variation of the substituent R in 4-position of the pyridine unit. Record player molecules synthesized in this work (**1f–j**) are highlighted.

Molecular spin switches operated with visible light in homogeneous solution [1] or on surfaces [2], hold promise for a number of hitherto unprecedented applications such as switchable contrast agents [3–7], switchable NMR relaxation agents [8–10], and building blocks for molecular spintroncis [11]. Our modular design is based on three components: a) a Ni(II)-porphyrin (square planar base complex), b) an azoaryl unit (photoswitchable ligand) and c) a molecular linker or tether (connecting the porphyrin with the switchable ligand). This approach has been dubbed “record player” design for obvious reasons (Figure 1a).

The Ni-porphyrin (“disk”) is low-spin if the azo-aryl unit (“tone arm”) is in *trans* configuration. Upon switching to the bent *cis* configuration, the lone pair of the pyridine (or imidazole) nitrogen (“needle”) is placed onto the Ni ion. The coordination number of the Ni^2+^ ion changes from 4 (square planar) to 5 (square pyramidal) and concomitantly, the spin state changes from low-spin (singlet, diamagnetic) to high-spin (triplet, paramagnetic). Previous studies have shown that all three components (Ni-porphyrin, azo-aryl unit, and tether) have to be carefully designed and tuned regarding their geometry and electronic properties. The Ni-porphyrin should be electron deficient (electron-withdrawing substituents in *meso* position), the azo-aryl unit should be electron rich (electron-donating substituents at the pyridine or imidazole part) to increase the Ni–ligand coordination strength. Strong coordination in turn improves the performance of the spin switch, namely the conversion rate to the *cis* isomer, the conversion rate to the high-spin state, as well as the thermal stability of the high-spin state. 4-Substituted pyridines exhibit a distinguished correlation between basicity and coordination strength as axial ligands with Ni-porphyrins [12]. Hence, Hammet σ values might be used to predict and to systematically optimize the performance of the spin switch. In previous studies we have shown that methoxy substitution (σ = −0.27) improved the spin switching efficiency in organic solvents to >98% in both directions [13]. However, the switching efficiency drops in protic solvents, particularly in water [3,14]. We now synthesized sulfur and carboxylic acid substituted record players aiming at the improvement of the switching efficiency. Thiol (σ = 0.15) and thiolethers (σ ≈ 0.0) are less electron donating than methoxy substituents, however, upon deprotonation, the thiolate (σ = −1.2) should considerably improve coordination and potentially restore switchability in water. Carboxylic acids are electron withdrawing (σ = 0.45), however, they are almost completely dissociated at neutral (physiological) pH. Carboxylate anion substituents are weakly electron donating and should improve both switching efficiency and water solubility.

## Results and Discussion

### Synthesis

The record player molecules **1f–j** (Figure 1) were synthesized by a modular approach described by Heitmann et al. [15]. The boronic ester **22** was prepared according to a mixed aldehyde procedure [16–17] and the substituted azopyridines **14**, **18**, **20**, and **21** were attached using Suzuki conditions.

#### Synthesis of azopyridines **10**–**12**, **14**, **16**–**18**, **20**, and **21**

**Scheme 1:**
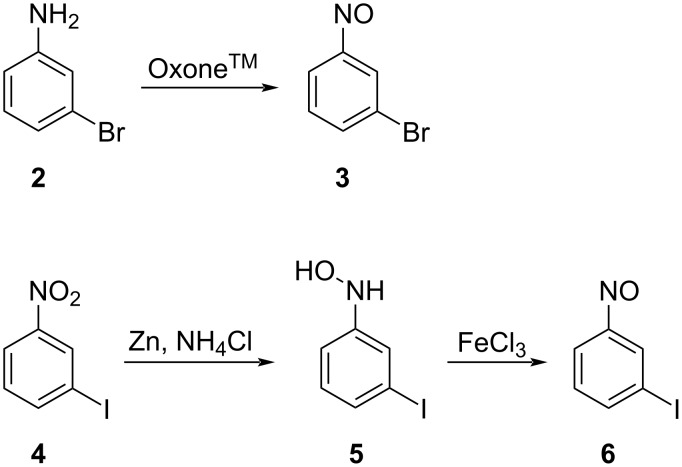
Synthesis of the nitroso compounds **3** and **6** using the two different methods described by Wegner et al. [19–21] and Dommaschk et al. [4,27].

For the synthesis of the azopyridines, Baeyer–Mills reactions [18] with 4-substituted 3-aminopyridines were applied [4]. The nitroso compounds **3** and **6** were synthesized by two different methods. 1-Bromo-3-nitrosobenzene (**3**) was obtained by oxidation of 3-bromoaniline (**2**) using Oxone^™^ (Wegner et al. [19–21], Scheme 1). Isolation of **3** was achieved, however, a one pot approach including a subsequent Baeyer–Mills reaction to yield **10** is preferred. 1-Iodo-3-nitrosobenzene (**6**) cannot be prepared by oxidation of the corresponding aniline because hypervalent iodine is formed [22–26]. Hence, 3-iodonitrobenzene (**4**) was reduced to obtain hydroxylamine **5**, which was oxidized by iron(III) chloride to yield a mixture of **6** and starting material **4** (Scheme 1), which was directly used as a crude product in the subsequent alkaline Baeyer–Mills reaction.

**Scheme 2:**
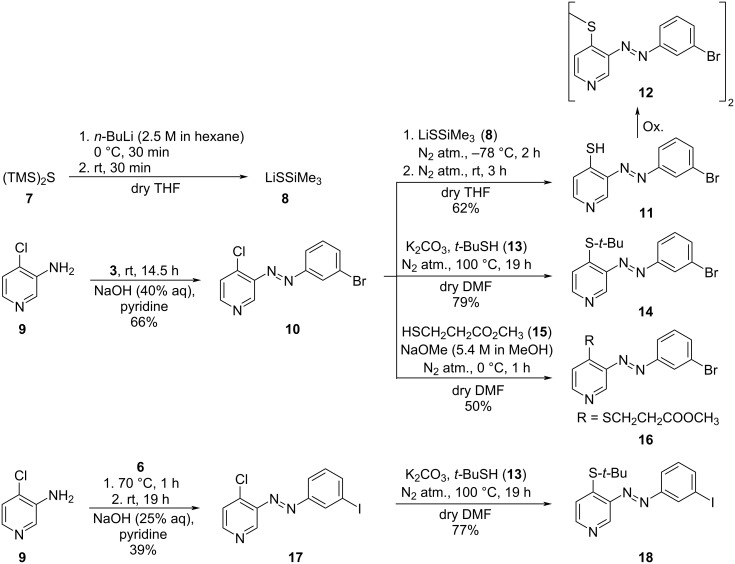
Synthesis of azopyridines **11**, **14**, **16** and **18** by nucleophilic aromatic substitution.

It is known that the yields of the Baeyer–Mills reactions, if performed under basic conditions, are higher for electron-deficient amines. 4-Chloropyridines are susceptible to nucleophilic aromatic substitutions [4], which was confirmed by the successful substitution of 3-(3-bromophenylazo)-4-chloropyridine (**10**) and 3-(3-iodophenylazo)-4-chloropyridine (**17**) with LiSSiMe_3_ (**8**) [28], *t*-BuSH (**13**) and HSCH_2_CH_2_CO_2_CH_3_ (**15**) [29]. Electron-deficient aromatic, silylated thiols exhibit very labile Si–S bonds [30]. Thus, even bulky silyl protection groups are not suitable as protecting groups for the subsequent Suzuki reaction. Azopyridines **14**, **16** and **18** were prepared by nucleophilic substitution with 2-methylpropane-2-thiol (**13**) and methyl 3-mercaptopropionate (**15**) as nucleophiles (Scheme 2).

**Scheme 3:**
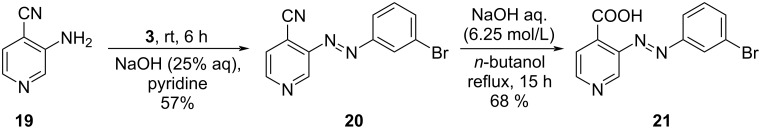
Synthesis of 3-(3-bromophenylazo)-4-cyanopyridine (**20**), which was hydrolyzed to yield 3-(3-bromophenylazo)-4-pyridinecarboxylic acid (**21**).

3-(3-Bromophenylazo)-4-cyanopyridine (**20**) was directly obtained by conversion of 3-amino-4-cyanopyridine (**19**) and **3**. Alkaline hydrolysis as described by Sato et al. [31] of **20** yielded 3-(3-bromophenylazo)-4-pyridinecarboxylic acid (**21**, Scheme 3).

#### Synthesis of the Ni(II)-porphyrins

**Scheme 4:**
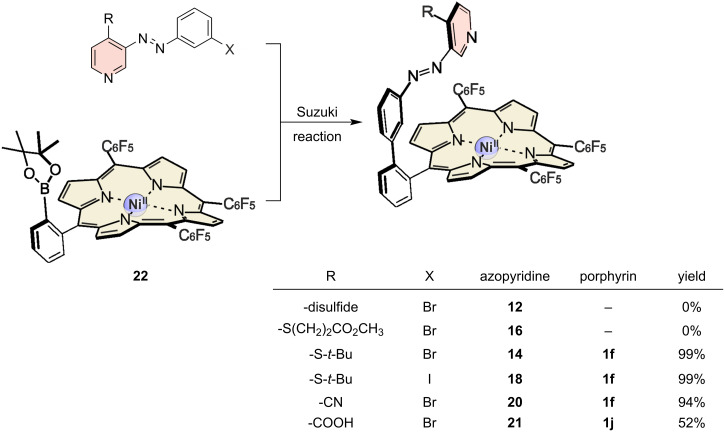
Modular approach for the C–C connection of the Ni(II)-porphyrin **22** and the different 4-substituted azopyridines **12**, **14**, **16**, **18**, **20**, and **21**.

The phenylboronic acid pinacol ester **22** was used as a component for the Suzuki reaction (Scheme 4).

**Scheme 5:**
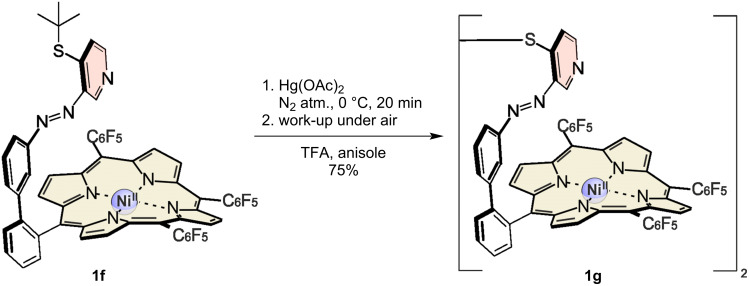
Cleavage of **1f** to yield disulfide **1g** [34].

No conversion was observed for the Suzuki reaction of the porphyrin **22** and the disulfide **12**, obviously because of catalyst poisoning by sulfur [32–33]. To circumvent these problems, protection groups were introduced. Protection with methyl 3-mercaptopropionate (**15**) [29] was successful, however, inhibition of the Suzuki reaction was observed. We assume an alkaline cleavage of the methyl 3-mercaptopropionate under the Suzuki conditions. Nevertheless, azopyridines **14**, **18** and **20** were converted almost quantitatively to the corresponding cross-coupling products, while the yield with **21** to **1j** is only 52%. The *tert*-butyl protection group on **1f** was cleaved according to a procedure of Nishimura et al. using mercury acetate in trifluoroacetic acid and anisole as radical scavenger (Scheme 5) [34]. Cleavage of the *tert*-butyl protection group yielded the disulfide **1g**, which was reduced with catecholborane in tetrahydrofuran to give the free thiol **1h**, which readily reoxidizes under air to form the disulfide.

### Investigation on the spin switching performance of the Ni(II)-porphyrins

The spin switching performance of a record player molecule depends on different parameters: 1. the coordination strength of the axial ligand, 2. the effectivity of the *cis–trans* isomerization, since only the *cis* isomer coordinates to the Ni(II) for steric reasons and 3. intermolecular coordination. At higher concentrations dimer formation or coordination oligomers between *trans* record player molecules occurs [35].

To compare the new molecules (**1f–j**) with previous systems (**1a**–**d**), the switching efficiencies were calculated based on the shift of the pyrrole protons relative to an independently synthesized fivefold coordinated Ni-porphyrin, which was used as a reference for a completely coordinated 5-coordinate Ni-porphyrin [13] (Table 1). Since coordination and decoordination is fast on the NMR time scale, the average pyrrole shift is a very accurate indicator of the proportion of species with and without a coordinating axial ligand (100% coordination (paramagnetic) 48.9 ppm and 0% coordination (diamagnetic) 9.1 ppm in acetone-*d*_6_).

**Table 1:** Switching states and switching efficiencies (Δ) of record player molecules **1a–j** in the PSS at 505 nm and 435 nm in acetone as a non-coordinating solvent. %*_cis-_*_para_ and %*_cis-_*_dia_ are defined as the proportion of the *cis* isomer in the paramagnetic and diamagnetic state, and %*_trans_* is the total proportion of the *trans* isomer, which is assumed to be completely diamagnetic at low concentrations.

	R	PSS 505 nm			PSS 435 nm				Δ
					
		%*_cis_*_-para_	%*_cis_*_-dia_	%*_trans_*		%*_cis_*_-para_	%*_cis_*_-dia_	%*_trans_*	%

**1a**^a^	-H	54	11	35		<5		>95	54
**1b**^a^	-Me	82	3	15		<5		>95	82
**1c**^a^	-OMe	>95	<1	<5		<5		>95	>95
**1d**^a^	-Cl	47	15	38		<5		>95	47
**1e**^b^	-OH	42	2	56		16	1	83	26
**1e^−^**^b^	-O^−^	72	<1	28		45	<1	55	27
**1f**	-S-*t*-Bu	70	3	27		<5		>95	70
**1g**	-[S]_2_-	–^c^	–^c^	–^c^		–^c^	–^c^	–^c^	–^c^
**1h**^b^	-SH	41	<1	59		13	<1	87	28
**1h^−^**^b^	-S^−^	44	2	54		10	<1	90	34
**1i**	-CN	11	16	73		<5		>95	11
**1j**^b^	-COOH	90	<1	10		13	<1	87	77
**1j^−^**^b^	-COO^−^	93	<1	7		10	<1	90	83

^a^Recalculated from Dommaschk et al. [4], ^b^determined by UV–vis spectroscopy, ^c^not determined because of very strong intramolecular coordination (Supporting Information File 1, Figure S17).

It should be noted that the *cis*/*trans* ratio does not necessarily correspond to the coordinated (paramagnetic)/decoordinated (diamagnetic) ratio because the *cis* isomer does not always coordinate completely and the *trans* isomer still can coordinate intermolecularly. The *cis*/*trans* ratios in the photostationary state (PSS) at 505 nm and 435 nm were investigated by independent NMR experiments. For **1f** and **1i** the *cis/trans* ratio was obtained from integration of the signals of *H*-11, which differ in *cis* and *trans* configuration. (Supporting Information File 1, Figure S14). However, NMR spectroscopy was not suitable to determine the *cis–trans* isomerization yields of **1e**, **1g**, **1h** and **1j** because of paramagnetic line broadening and overlapping signals. To circumvent these problems, UV–vis spectroscopy was performed at very low concentrations (Table 1). The porphyrin Soret bands are different for the diamagnetic (λ_max_ ≈ 406 nm) and the paramagnetic species (λ_max_ ≈ 423 nm). The molar extinctions of both species were determined by adding a strong axial ligand such as piperidine (to achieve almost complete coordination) and acid (TFA) (protonation of the pyridine unit to prevent coordination, completely diamagnetic). At concentrations of ≈10 µM, intermolecular coordination is negligible. Thus, the absorption band at 423 nm provides information of the total amount of paramagnetic *cis* species and the band at 406 nm comprises the total amount of diamagnetic *trans* and uncoordinated *cis* isomer. Combined with the information from NMR the *cis/trans* ratios were determined.

Disulfide **1g** is always in a high paramagnetic state (>80%) and showed only minor switching efficiency after irradiation (4%). This is attributed to intramolecular coordination of the *trans* state (Supporting Information File 1, Figure S17). Application of density functional theory (DFT) at the B3LYP/def2TZVP//PBE/def2SVP level of theory (for details see Supporting Information File 1) revealed that the thermodynamically most stable structure is a *trans* configuration, where each of the pyridine units coordinate the nickel of the opposite porphyrin. Reductive cleavage of **1g** by catecholeborane yields the photoswitchable thiol **1h**.

**Figure 2:**
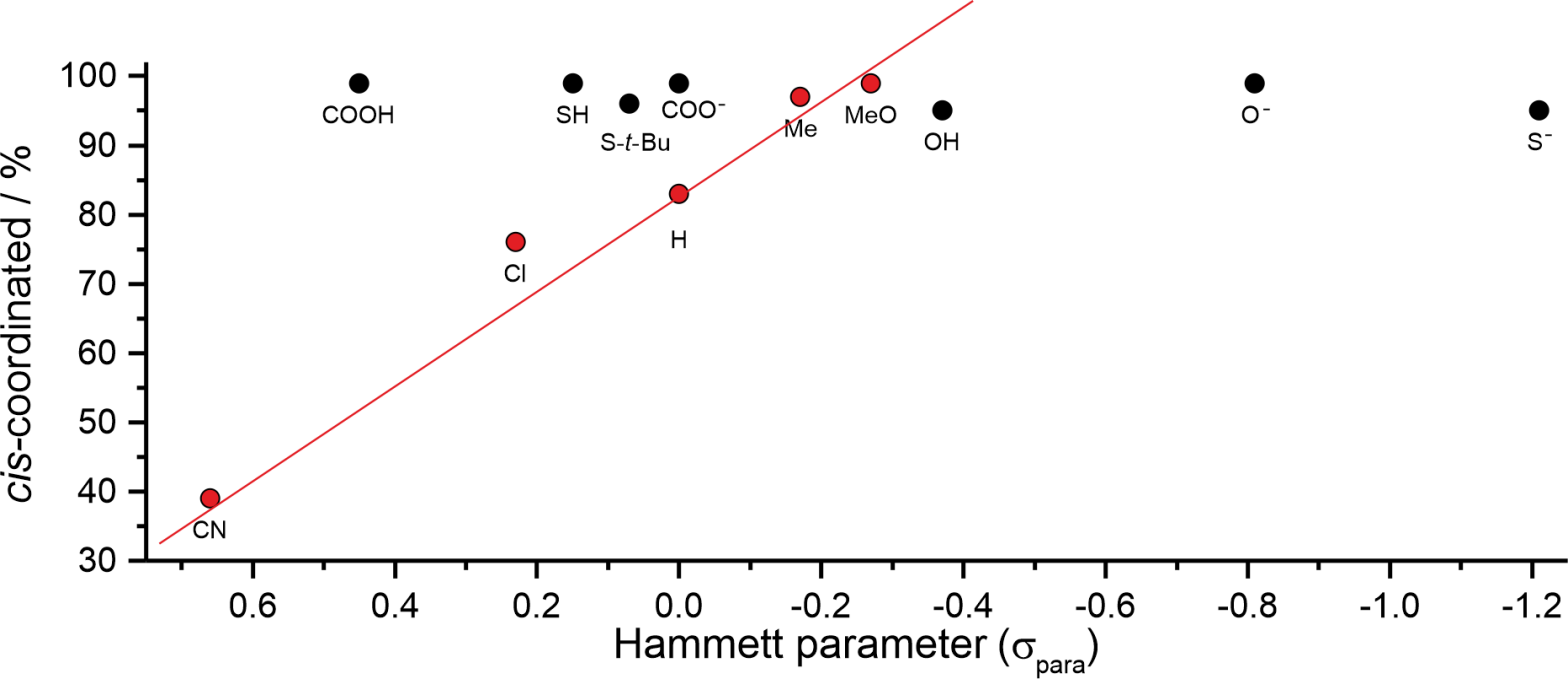
Hammett plot of the investigated pyridine substituents [36].

Table 1 summarizes the photostationary states at 505 and 435 nm, the diamagnetic/paramagnetic ratio (%*_cis-_*_dia_/%*_cis-_*_para_) of the *cis* isomer for compounds **1a–j** and the spin switching efficiency (Δ). The spin switching efficiency is defined as the difference of the percentage of the diamagnetic (or the paramagnetic) species in the two photostationary states. The intermolecular association constants of pyridines as axial ligands to Ni-porphyrins as a function of their *para* substituents usually follow the Hammett relationship [5,12]. Hence, the association constants should also be reflected in the intramolecular coordination of the covalently bound pyridine units within the *cis* isomers (**1a–j**). Whereas the substituents R = H, S-*t*-Bu, Me, OMe, Cl and CN very roughly comply with this assumption, the protic and deprotonated substituents R = OH, O^−^, SH, S^−^, COOH, COO^−^ strongly deviate (Figure 2). The substituents COOH, SH, COO^−^ and O^−^ give rise to almost complete coordination of the *cis* isomer. In contrast to bimolecular coordination [12] the intramolecular coordination in the substituted *cis* isomers, obviously, does not follow a clear linear free energy relationship. Steric hindrance or strain might be the reasons for the deviations. We cannot exclude that these groups coordinate directly to the Ni ion.

Whereas the thiol (**1h**) and thiolate (**1h^−^**) exhibit disappointingly low switching efficiencies (Δ = 28% and 34%), the carboxylic acid (**1j**) and the corresponding carboxylate anion (**1j**^−^) perform surprisingly well (Δ = 77% and 83%).

**Figure 3:**
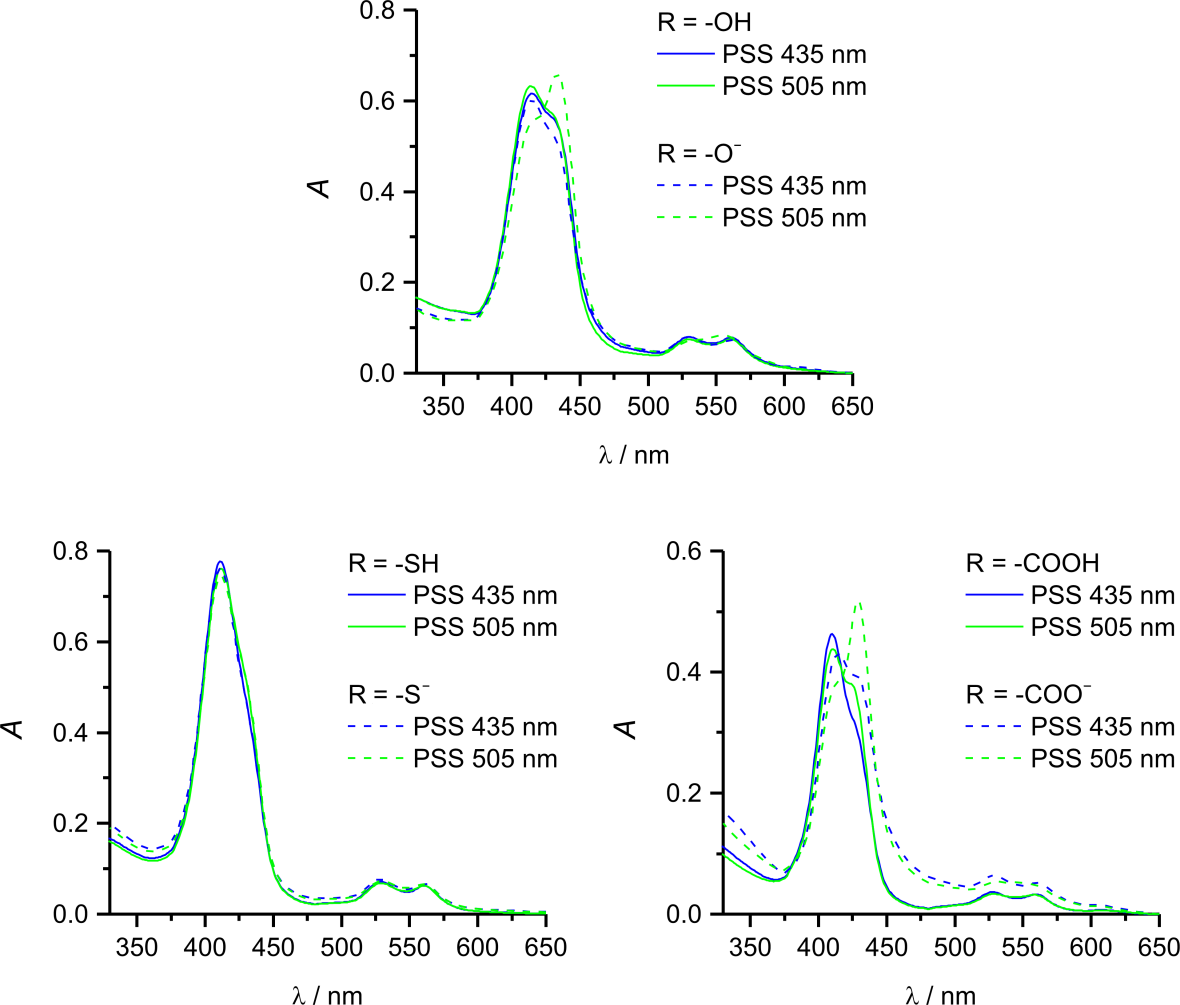
UV–vis spectra of **1e** (top), **1h** (left) and **1j** (right) in acetone water (1:9) (solid line) and after deprotonation using cesium carbonate (dashed line) in the PSS at 505 nm (green) and 435 nm (blue).

In order to check whether the switching efficiencies would decrease in aqueous environment to a similar extend as in previous cases [3], switching experiments with the protic record players **1e** (R = OH), **1h** (SH) and **1j** (R = COOH) in acetone/water 1:9 were performed. UV experiments revealed that the switching efficiencies of the protonated species indeed dropped dramatically as compared to those in pure acetone. However, the deprotonated 4-hydroxypyridine **1e^−^** and the pyridine-4-carboxylate **1j^−^** still perform with switching efficiencies of 40% and 28% in aqueous environment (Figure 3). If it comes to medical applications, such as switchable contrast agents [3–7], the new carboxylate substituted system **1j** has the distinct advantage of being deprotonated at neutral pH, whereas the previously synthesized hydroxylate system **1e** exhibits its highest switching efficiency at unphysiological pH 10.5 [14]. Hence, further attempts to optimize our molecular spin switches will be based on pyridine-4-carboxylate-substituted systems.

## Conclusion

Aiming at the optimization of molecular spin switches (record player molecules) towards higher switching efficiencies, particularly in water, we synthesized 5 new record player molecules (**1f–j**). Water forms H-bonds with the pyridine units, and reduces the coordination power to Ni^2+^ and thus decreases the efficiency of conversion to the paramagnetic (coordinated) state. Insufficient water solubility is another notorious problem of porphyrin-based spin switches. To kill two birds with one stone, we introduced acidic substituents in 4-position of the pyridines (**1h** (R = SH), **1j** (R = COOH)). Deprotonation to the corresponding anions should increase the coordination power of the pyridine and increase the solubility of the record player molecules. Surprisingly, the thiolate exhibits a low switching efficiency (Δ = 34%), however, the carboxylate performs quite well (Δ = 83%) in acetone and even in aqueous environment.

## Experimental

Detailed information on the experimental procedures are given in Supporting Information File 1.

## Supporting Information

File 1 Experimental procedures and spectra.
