# Supplementary material for: Synthesis of 4-substituted azopyridine-functionalized Ni(II)-porphyrins as molecular spin switches
Source: Beilstein J Org Chem. 2020 Oct 21;16:2589–97. doi: 10.3762/bjoc.16.210 (PMC7590622; doi:10.3762/bjoc.16.210)
Supplement: File 1 — Experimental procedures and spectra. [file Beilstein_J_Org_Chem-16-2589-s001.pdf]

BEILSTEIN JOURNAL OF ORGANIC CHEMISTRY

Supporting Information

for

Synthesis of 4-substituted azopyridine-functionalized Ni(II)-porphyrins as molecular spin switches

Jannis Ludwig, Tobias Moje, Fynn Röhricht and Rainer Herges

Beilstein J. Org. Chem. **2020,** *16,* 2589–2597. doi:10.3762/bjoc.16.210

Experimental procedures and spectra

License and Terms: This is a supporting information file under the terms of the Creative Commons Attribution License (https://creativecommons.org/licenses/by/4.0). Please note that the reuse, redistribution and reproduction in particular requires that the authors and source are credited.
The license is subject to the *Beilstein Journal of Organic Chemistry* terms and conditions: (https://www.beilstein-journals.org/bjoc)

I Equipment

II Synthetic procedure and NMR spectra

III Procedure for photophysical experiments

IV References

I: Equipment

Nuclear magnetic resonance (NMR) spectroscopy

The ^{1}H NMR measurements were carried out on a Bruker AvanceNeo 500 or a Bruker Avance 600 spectrometer at 500 MHz or 600 MHz in deuterated solvents. The solvent residual signals were used as internal reference ($CDCl_3$: δ = 7.26 ppm, acetone-d_6: δ = 2.05 ppm, methanol-d_4: δ = 3.31 ppm) [1]. ^{13}C NMR measurements were carried out on the above spectrometers at 126 MHz or 151 MHz and the solvent residual signals were used as internal reference ($CDCl_3$: δ = 77.16 ppm, acetone-d_6: δ = 29.84 ppm, methanol-d_4: δ = 49.00 ppm) [1]. Standard 2D-spectra (COSY, HSQC, HMBC) were used to assign the signals. ^{19}F NMR measurements were carried out on a Bruker AvanceNeo 500 spectrometer at 471 MHz. Samples containing deuterated trifluoroacetic acid (TFA-d_1) to make substances soluble or completely diamagnetic are especially labelled.

Mass spectrometry (MS)

Electron ionization (EIMS) and high resolution electron ionization mass spectrometry (HREIMS) was carried out on a Joel AuccTOF 4GCV mass spectrometer after electron ionization (EI) at 70 eV. High resolution electrospray ionization mass spectrometry (HRESIMS) was carried out on a Thermo Fisher Scientific Q Exactive™ UHMR Hybrid Quadrupole-Orbitrap™ mass spectrometer. Matrix-assisted laser desorption/ionization time of flight mass spectrometry (MALDI–TOF–MS) was carried out on a Bruker Biflex III mass spectrometer using 4-chloro-α-cyanocinnamic acid (Cl-CCA) as matrix.

UV–vis spectroscopy

Ultraviolet visible (UV–vis) spectra were recorded on a Perkin Elmer Lambda 650 spectrometer with a Quantum Northwest Inc. qpod 2e sample holder in quartz cuvettes with a path length of 10 mm.

Irradiation modules

Samples were irradiated with an irradiation module containing 12 Roithner VL-440-Emitter LEDs (435 nm) and 12 Nichia NS6E083 LEDs (505 nm) manufactured by Sahlmann Photochemical Solutions. During the irradiation process samples were cooled by an airstream if necessary cooled over liquid nitrogen.

Infrared (IR) spectroscopy

Fourier transform infrared spectroscopy (FTIR) was carried out on a Perkin Elmer Spectrum 100 spectrometer with A531-G Golden-Gate-Diamond attenuated total reflection (ATR) unit. The signal intensity is given as s = strong, m = medium, w = weak, br = broad.

Elemental analysis (EA)

The amount of carbon, hydrogen, nitrogen and sulfur in a compound was determined using an Elementar Vario Micro Cube.

Melting points

Melting points (m.p.) were measured using a Büchi Melting Point M 560 in one-sided open tubes and are given uncorrected in °C.

Chromatography

Silica gel (Merck, particle size 0.040–0.063 mm) was used for column chromatography. If indicated, a Biotage® Isolera™ Spektra One flash purification chromatography system was used with the named columns. R_f values were determined by thin layer chromatography on Polygram® Sil G/UV_{254} (Macherey-Nagel, 0.2 mm particle size).

II: Synthetic procedure and NMR spectra

Synthesis of 1-bromo-3-nitrosobenzene (3)

3-Bromoaniline (2.50 g, 14.5 mmol, **2**) was dissolved in 50 mL of dichloromethane, and Oxone™ (22.1 g, 145 mmol) in 125 mL deion. water was added. The mixture was stirred at ambient temperature for 4 h. Layers were separated and the aqueous layer was extracted with dichloromethane (3 × 40 mL). The combined org. layers were dried over magnesium sulfate and the solvent was removed in vacuo. The crude product was purified by flash column chromatography (Biotage, SNAP Ultra cartridge HP-Sphere 100 g, dichloromethane, R_f = 0.83). The product was obtained as a yellow-green solid.

Yield: 2.20 g (11.9 mmol, 82%), Lit.: 72% [2]

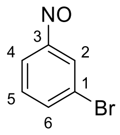

M.p.: 77 °C, Lit.: 77 °C [3]

^{1}H NMR (500 MHz, $CDCl_3$, 300 K): δ = 8.12 (ddd, ^3J = 7.85 Hz, ^4J = 1.96 Hz, ^4J = 1.10 Hz, 1H, *H*-6), 7.85 (ddd, ^3J = 7.91 Hz, ^3J = 1.94 Hz, ^4J = 1.05 Hz, 1H, *H*-4), 7.78 (t, ^4J = 1.88 Hz, 1H, *H*-2), 7.57 (t, ^3J = 7.95 Hz, 1H, *H*-5) ppm.

^{13}C NMR (126 MHz, $CDCl_3$, 300 K): δ = 165.1 (*C*-3), 137.9 (*C*-4), 131.0 (*C*-5), 123.8 (*C*-1), 121.9 (*C*-6), 121.7 (*C*-2) ppm.

EI-MS (70 eV): m/z (%) = 184.94 (53) $[M]^+$, 154.94 (100) $[M-NO]^+$.

HR-EI-MS (70 eV): m/z = 184.94763 (calcd. for C_6H_4BrNO), 184.94754 (found) (-0.48 ppm).

FT-IR (ATR): ν = 3086 (m), 1956 (w), 1892 (w), 1829 (w), 1577 (m), 1531 (m), 1465 (m), 1425 (m), 1348 (m), 1300 (m), 1249 (s), 1177 (m), 1159 (m), 1111 (m), 1089 (m), 1068 (m), 998 (m), 917 (m), 882 (m), 854 (m), 785 (s), 728 (m), 693 (s), 669 (s) cm^{-1}.

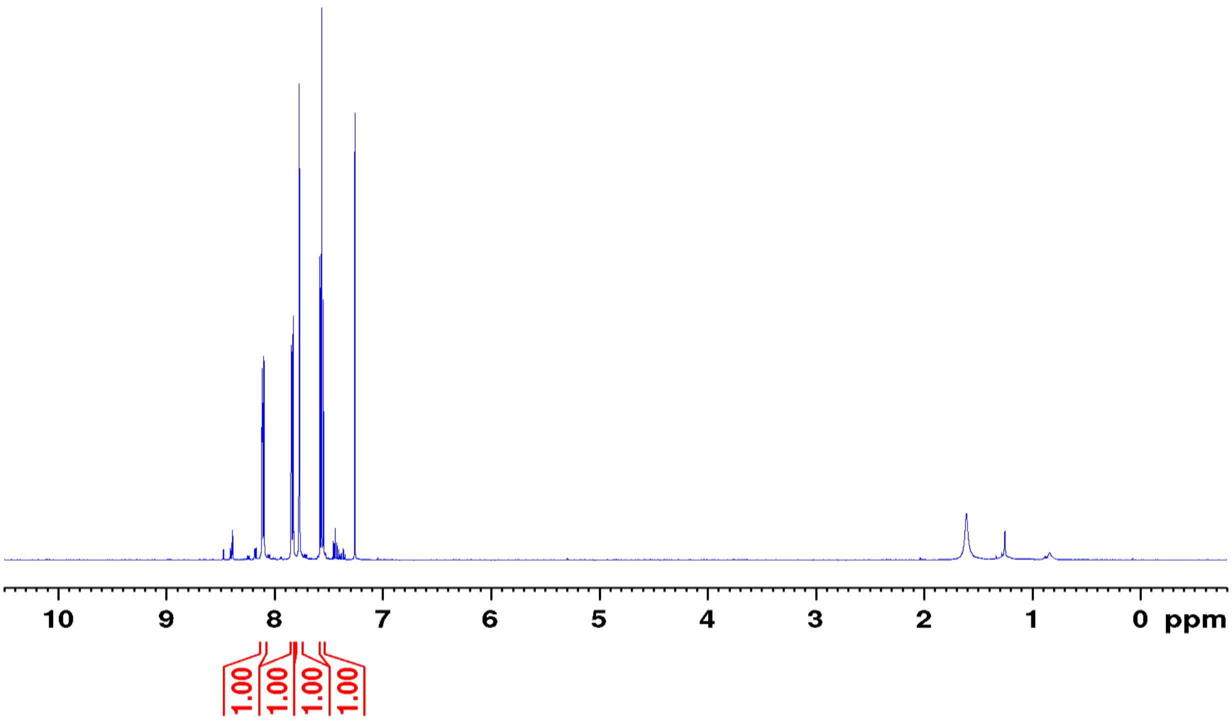

Figure S1.1: Full ^{1}H NMR spectrum of 1-bromo-3-nitrosobenzene (**3**) (500 MHz, $CDCl_3$, 300 K).

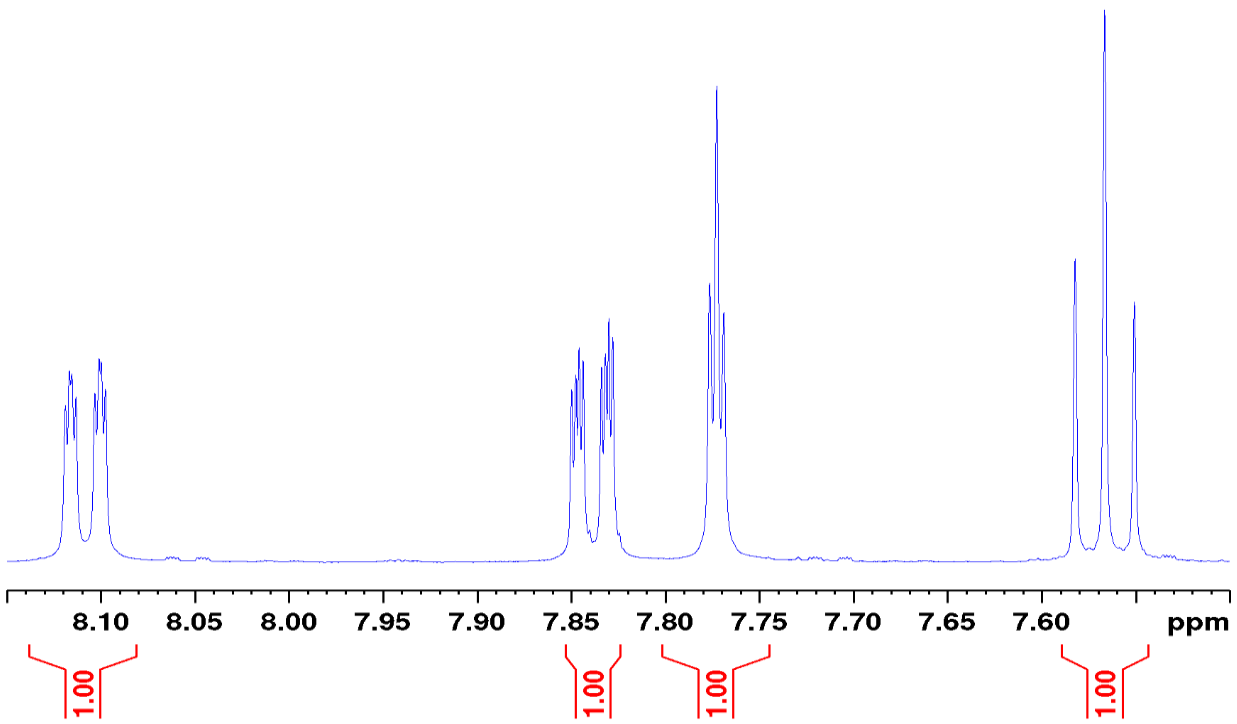

Figure S1.2: Aromatic section of the ^{1}H NMR spectrum of 1-bromo-3-nitrosobenzene (**3**) (500 MHz, $CDCl_3$, 300 K).

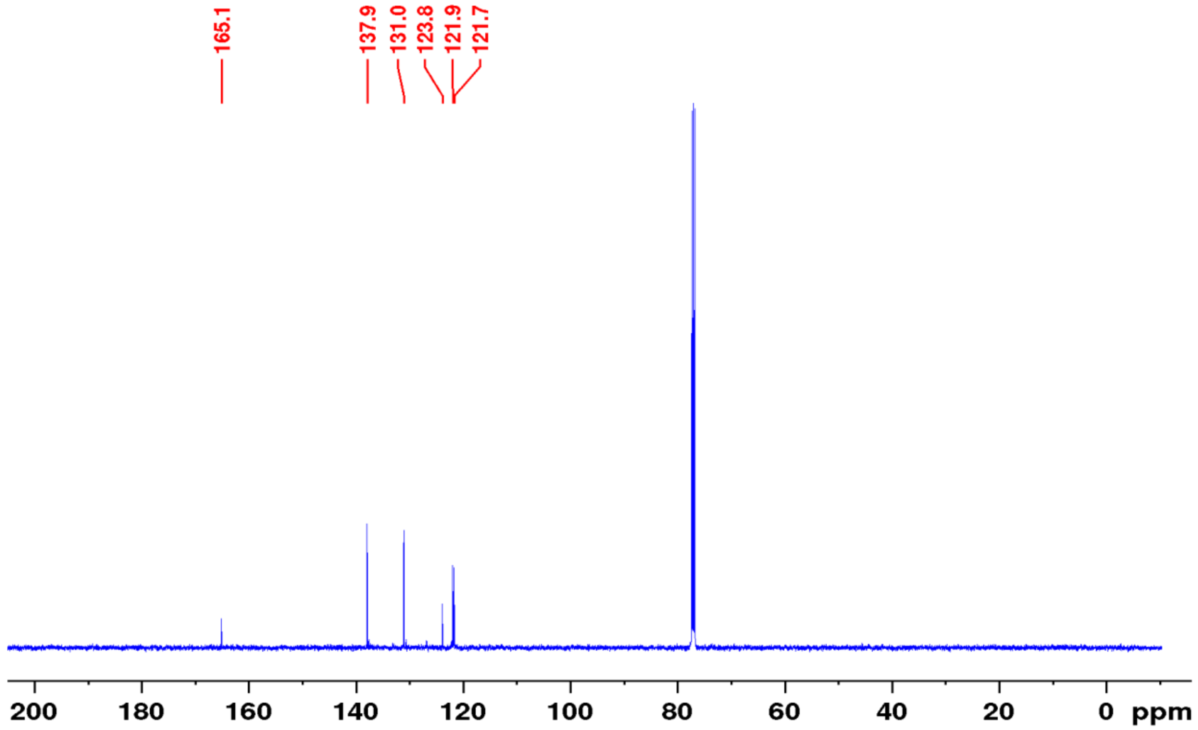

Figure S1.3: Full ^{13}C NMR spectrum of 1-bromo-3-nitrosobenzene (**3**) (126 MHz, $CDCl_3$, 300 K).

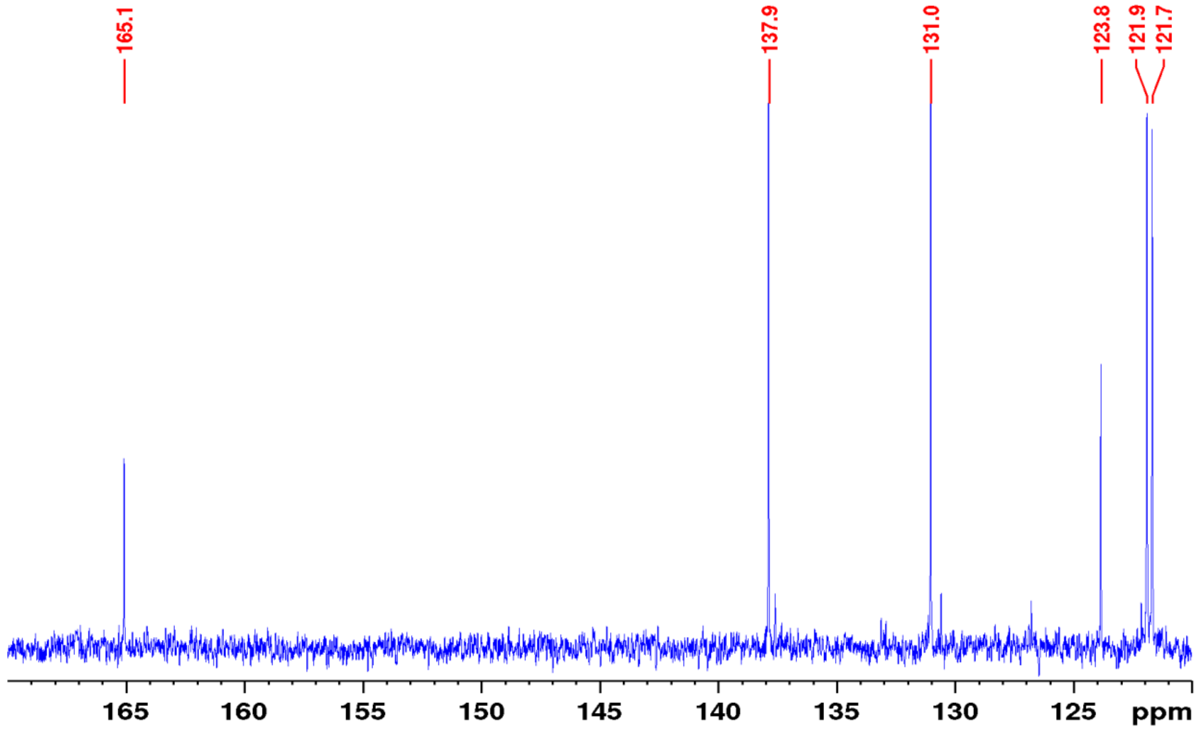

Figure S1.4: Aromatic section of the ^{13}C NMR spectrum of 1-bromo-3-nitrosobenzene (**3**) (126 MHz, $CDCl_3$, 300 K).

Synthesis of 3-(3-bromophenylazo)-4-chloropyridine (10)

3-Amino-4-chloropyridine (535 mg, 4.16 mmol, **9**) was dissolved in 40 mL pyridine and 15 mL sodium hydroxide solution (40% aq.) and 1-bromo-3-nitrosobenzene (1.00 g, 5.40 mmol, **3**) was added. The mixture was stirred for 14.5 h at ambient temperature, diluted with deion. water (150 mL) and extracted with ethyl acetate (4 × 100 mL). The combined org. layers were dried over sodium sulfate and the solvent was removed in vacuo. Remaining pyridine was removed by co-distillation with toluene (2 × 100 mL). The crude product was applied on silica and purified by flash column chromatography (Biotage, SNAP Ultra cartridge HP-Sphere 25 g, cyclohexane/ethyl acetate 32.33:1 → 2.57:1, R_f (3:1) = 0.59). The product was obtained as an orange solid.

Yield: 810 mg (2.75 mmol, 66%)

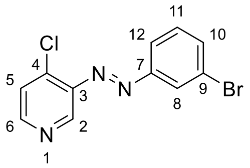

M.p.: 79 °C, Lit.: 78.1 °C [4]

^{1}H NMR (500 MHz, $CDCl_3$, 298 K): δ = 8.80 (s, 1H, *H*-2), 8.57 (d, ^3J = 5.36 Hz, 1H, *H*-6), 8.11 (t, ^4J = 1.88 Hz, 1H, *H*-8), 7.96 (ddd, ^3J = 7.97 Hz, ^4J = 1.85 Hz, ^4J = 1.03 Hz, 1H, *H*-12), 7.67 (ddd, ^3J = 7.96 Hz, ^4J = 1.97 Hz, ^4J = 1.03 Hz, 1H, *H*-10), 7.55 (d, ^3J = 5.36 Hz, 1H, *H*-5), 7.45 (t, ^3J = 7.96 Hz, 1H, *H*-11) ppm.

^{13}C NMR (126 MHz, $CDCl_3$, 298 K): δ = 153.4 (*C*-7), 151.6 (*C*-6), 144.2 (*C*-3, *C*-4), 139.3 (*C*-2), 134.9 (*C*-10), 130.6 (*C*-11), 125.7 (*C*-5), 125.3 (*C*-8), 123.6 (*C*-12), 123.3 (*C*-9) ppm.

EI-MS (70 eV): m/z (%) = 294.94 (28) $[M]^+$, 182.95 (49) $[M-C_5H_3ClN]^+$, 154.95 (100) $[M-C_5H_3ClN_3]^+$, 111.99 (58) $[M-C_6H_4BrN_2]^+$.

HR-EI-MS (70 eV): m/z = 294.95119 (calcd. for $C_{11}H_7BrClN_3$), 294.95079 (found) (-1.34 ppm).

FT-IR (ATR): ν = 3083 (w), 3040 (w), 1557 (s), 1476 (m), 1459 (m), 1416 (m), 1391 (m), 1299 (w), 1268 (w), 1247 (w), 1189 (m), 1152 (m), 1081 (m), 1062 (m), 995 (w), 929 (m), 886 (m), 826 (s), 788 (s), 731 (s), 677 (s), 653 (m), 582 (m), 545 (s), 532 (m) cm^{-1}.

EA (%): calcd. for $C_{11}H_7BrClN_3$: C: 44.55, H: 2.38, N: 14.17;
Found: C: 44.55, H: 2.41, N: 13.86.

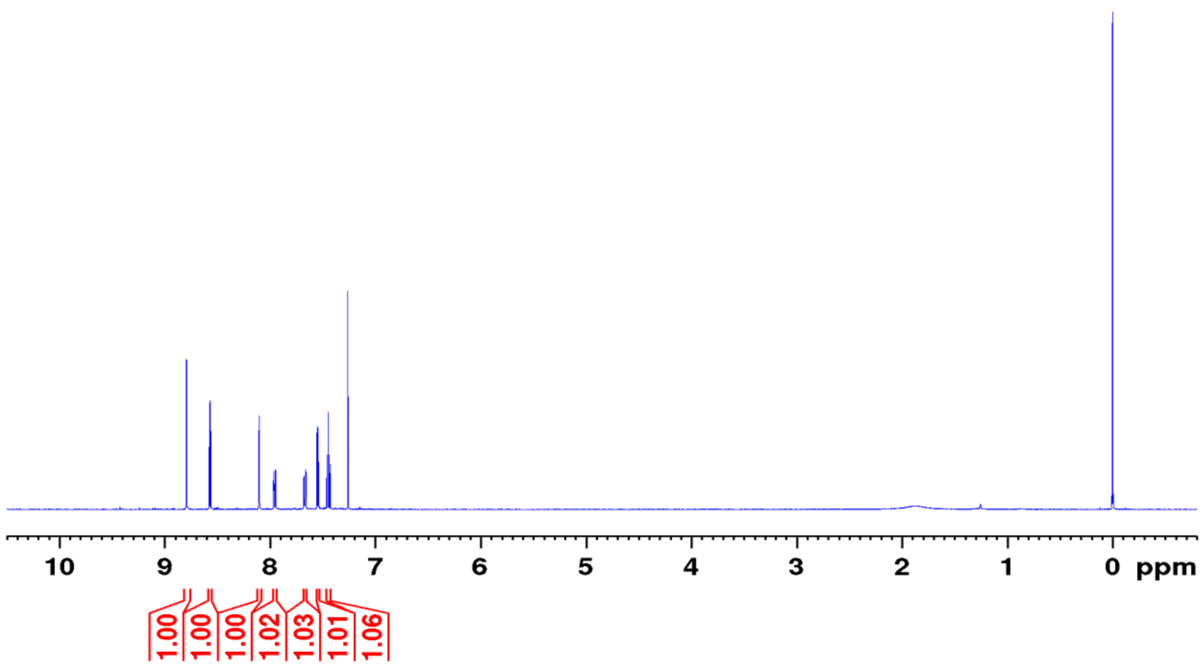

Figure S2.1: Full ^{1}H NMR spectrum of 3-(3-bromophenylazo)-4-chloropyridine (**10**) (500 MHz, $CDCl_3$, 298 K).

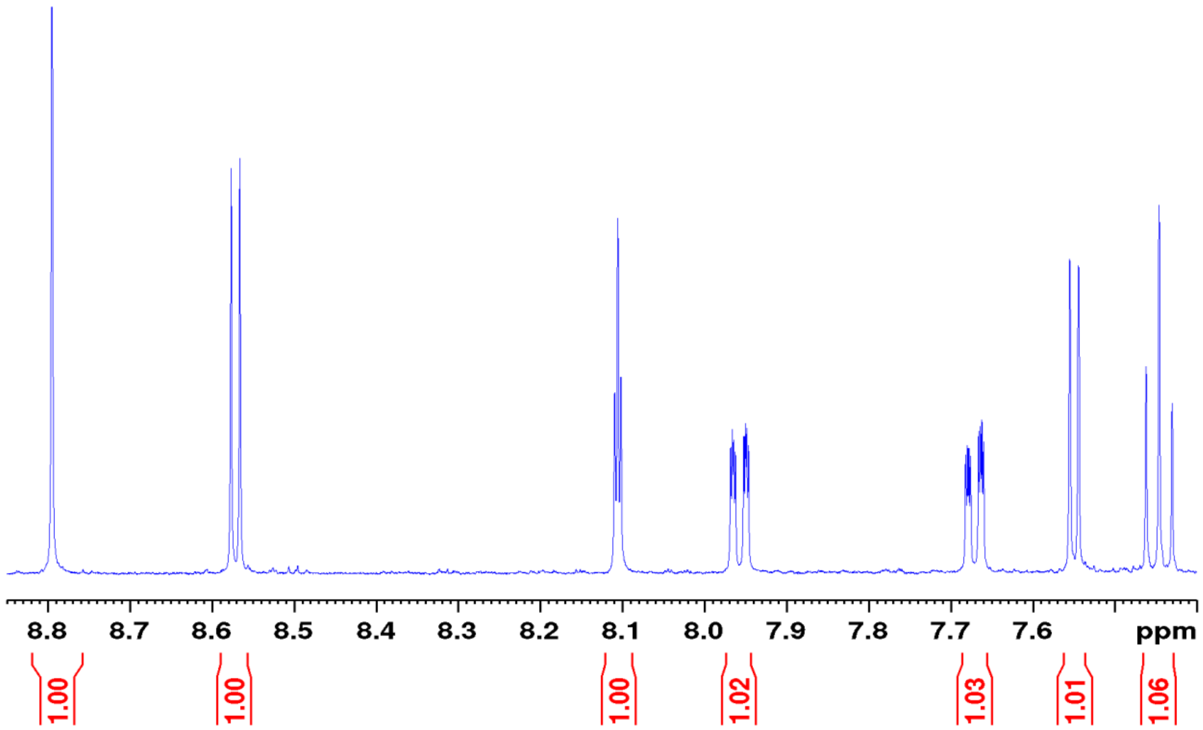

Figure S2.2: Aromatic section of the ^{1}H NMR spectrum of 3-(3-bromophenylazo)-4-chloropyridine (**10**) (500 MHz, $CDCl_3$, 298 K).

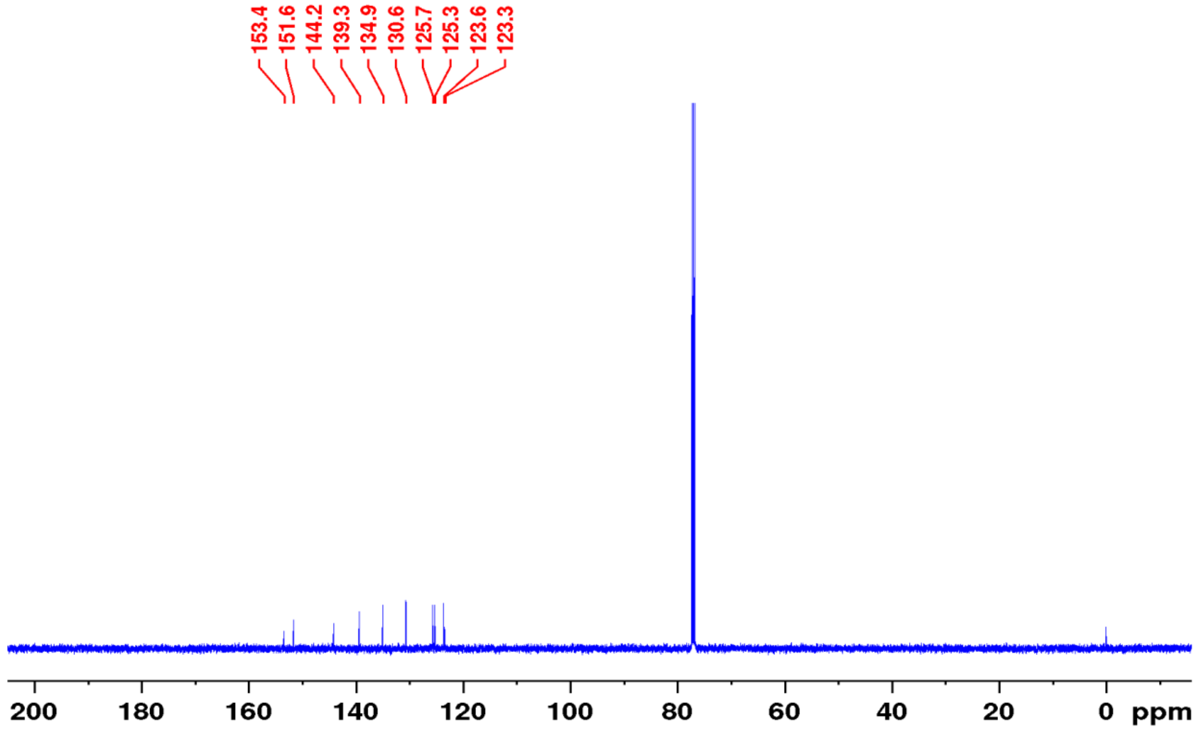

Figure S2.3: Full ^{13}C NMR spectrum of 3-(3-bromophenylazo)-4-chloropyridine (**10**) (126 MHz, $CDCl_3$, 298 K).

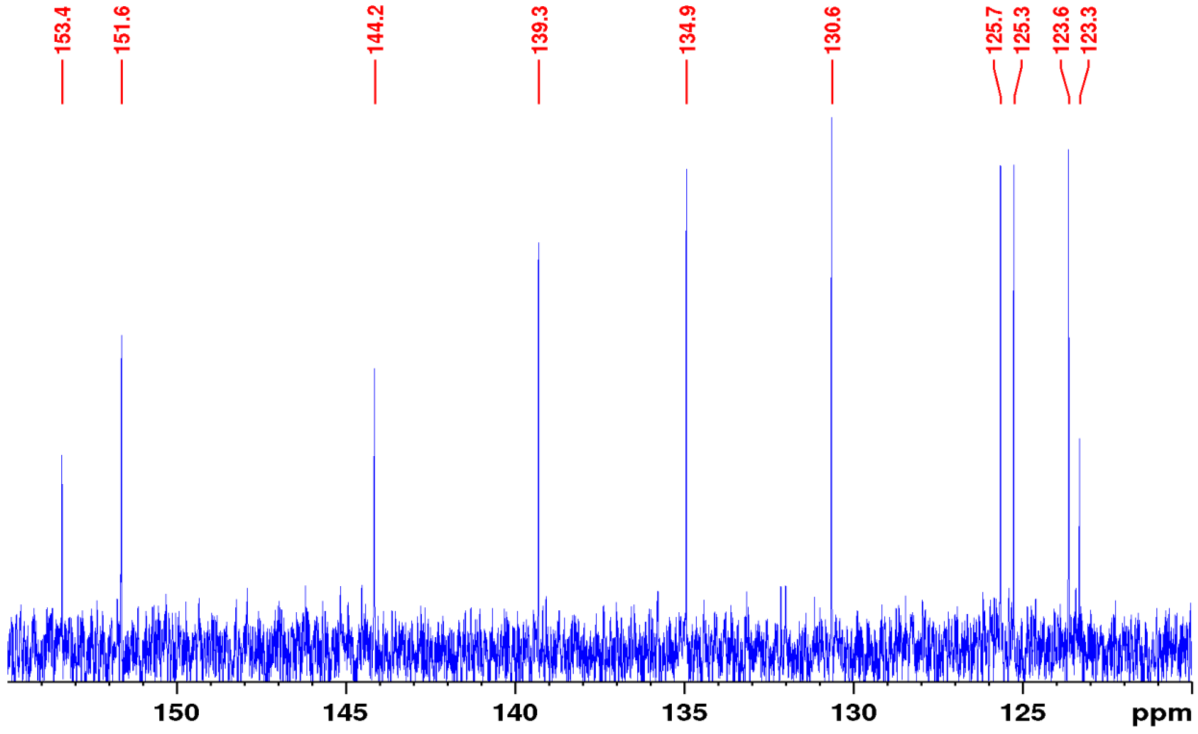

Figure S2.4: Aromatic section of the ^{13}C NMR spectrum of 3-(3-bromophenylazo)-4-chloropyridine (**10**) (126 MHz, $CDCl_3$, 298 K).

Synthesis of 3-(3-bromophenylazo)-4-mercaptopyridine (12)

In flame dried glassware under an atmosphere of nitrogen hexamethyldisilathiane (64.2 mg, 360 µmol, **7**) was dissolved in 7.6 mL of dry tetrahydrofuran and cooled to 0 °C. *n*-Buthyllithium (144 µL 2.5 M in hexane, 360 µmol) was slowly added and stirred for 30 min at 0 °C and further 30 min at ambient temperature. Separately, 3-(3-bromophenylazo)-4-chloropyridine (106 mg, 358 µmol, **10**) was dissolved in 3 mL of dry tetrahydrofuran. Both solutions were cooled to −78 °C, combined and stirred for 2 h at this temperature in darkness. Afterwards, the mixture was allowed to warm to ambient temperature and stirred for further 3 h in darkness. The mixture was diluted with ethyl acetate (30 mL), a saturated solution of sodium chloride (10 mL) and deionized water (20 mL). Layers were separated and the aqueous layer was extracted with ethyl acetate (7 × 20mL). The combined organic layers were dried over magnesium sulfate and the solvent was removed in vacuo*.* The crude product was purified by column chromatography (silica, cyclohexane/ethyl acetate 1.5:1, R_f (1.5:1) = 0.10). The product was obtained as an orange solid, the symmetric disulfide, which was formed under ambient workup conditions.

Yield: 65.0 mg (223 µmol, 62%)

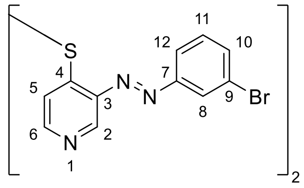

M.p.: 187 °C, decomposition

^{1}H NMR (500 MHz, $CDCl_3$, TFA-d_1, 298 K): δ = 9.40 (s, 2H, *H*-2), 8.74 (d, ^3J = 6.59Hz, 2H, *H*-6), 8.32 (d, ^3J = 6.59 Hz, 2H, *H*-5), 8.24 (t, ^4J = 1.82 Hz, 2H, *H*-8), 8.08 (ddd, ^3J = 8.01 Hz, ^4J = 1.67 Hz, ^4J = 0.89 Hz, 2H, *H*-12), 7.86 (ddd, ^3J = 8.00 Hz, ^4J = 1.69 Hz, ^4J = 0.90 Hz, 2H, *H*-10), 7.56 (t, ^3J = 8.01 Hz, 2H, *H*-11) ppm.

^{13}C NMR (126 MHz, $CDCl_3$, TFA-d_1, 298 K): δ = 155.0 (*C*-7), 151.6 (*C*-7), 146.6 (*C*-3), 140.2 (*C*-6), 138.5 (*C*-2), 138.1 (*C*-10), 131.5 (*C*-11), 126.2 (*C*-8), 124.2 (*C*-9), 124.1 (*C*-5), 123.9 (*C*-12) ppm.

EI-MS (70 eV): m/z (%) = 291.95 (82) $[C_{11}H_7BrN_3S]^+$, 154.95 (100) $[C_6H_3Br]^+$, 110.01 (57) $[C_5H_3NS]^+$.

HR-EI-MS (70 eV): m/z = 291.95441 (calcd. for $C_{11}H_7BrN_3S$), 291.95398 (found) (-1.44 ppm).

FT-IR (ATR): ν = 3065 (w), 2920 (w), 1797 (w), 1710 (w), 1563 (s), 1523 (m), 1456 (m), 1432 (m), 1410 (m), 1299 (w), 1256 (w), 1200 (s), 1147 (m), 1082 (m), 1059 (m), 995 (w), 934 (m), 923 (m), 880 (m), 851 (m), 823 (m), 808 (m), 791 (s), 724 (s), 678 (s), 655 (m) cm^{-1}.

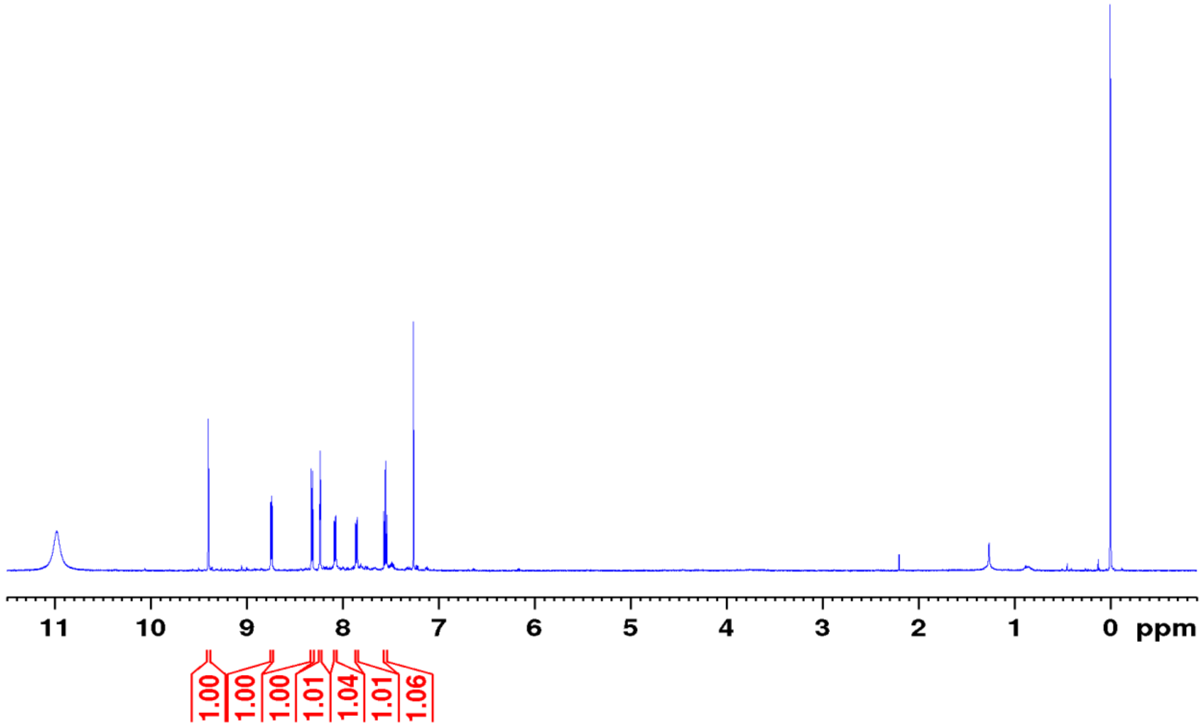

Figure S3.1: Full ^{1}H NMR spectrum of 3-(3-bromophenylazo)-4-mercaptopyridine (**12**) (500 MHz, $CDCl_3$, TFA-d_1, 298 K).

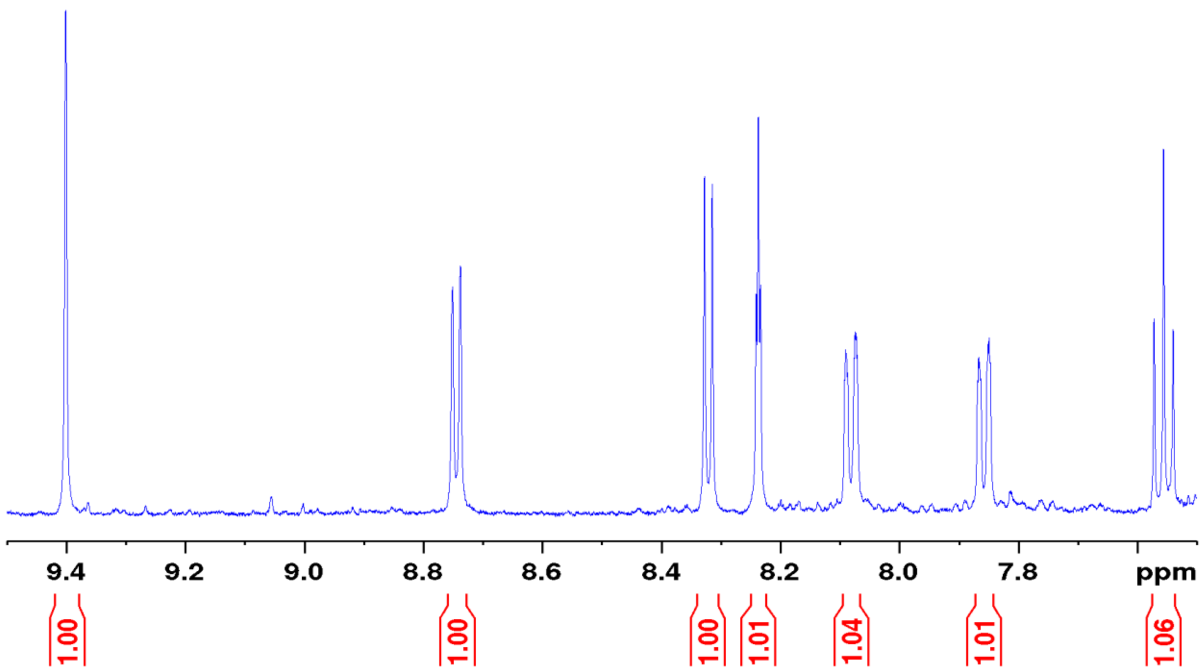

Figure S3.2: Aromatic section of the ^{1}H NMR spectrum of 3-(3-bromophenylazo)-4-mercaptopyridine (**12**) (500 MHz, $CDCl_3$, TFA-d_1, 298 K).

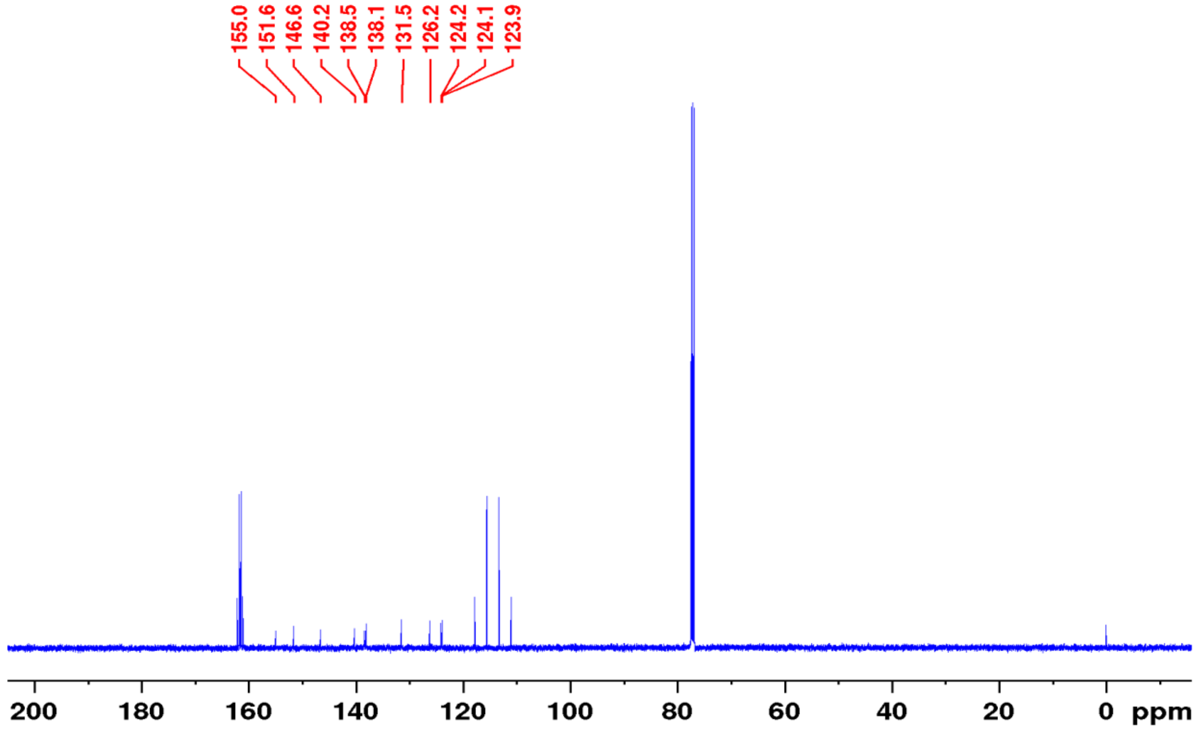

Figure S3.3: Full ^{13}C NMR spectrum of 3-(3-bromophenylazo)-4-mercaptopyridine (**12**) (126 MHz, $CDCl_3$, TFA-d_1, 298 K).

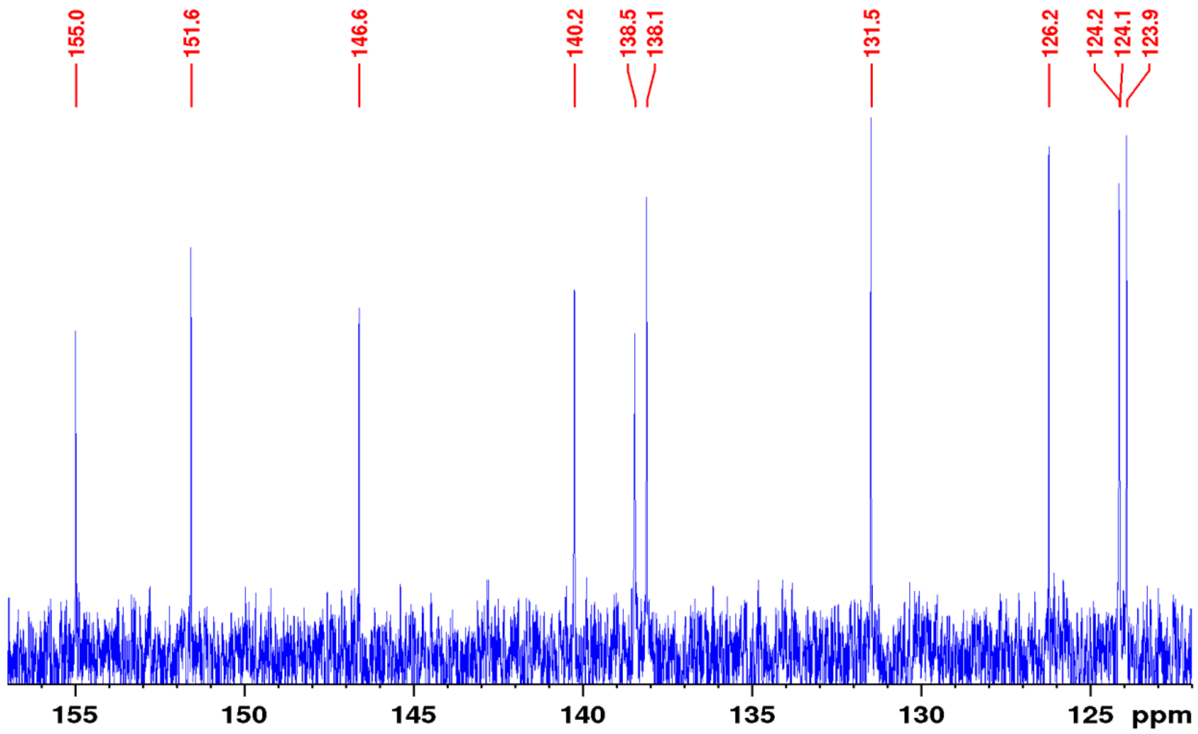

Figure S3.4: Aromatic section of the ^{13}C NMR spectrum of 3-(3-bromophenylazo)-4-mercaptopyridine (**12**) (126 MHz, $CDCl_3$, TFA-d_1, 298 K).

Synthesis of 3-(3-bromophenylazo)-4-*tert*-butylmercaptopyridine (14)

In flame dried glassware under an atmosphere of nitrogen 3-(3-bromophenylazo)-4-chloropyridine (250 mg, 843 µmol, **10**) and potassium carbonate (582 mg, 4.21 mmol) were dissolved in 100 mL of dry dimethylformamide. 2-Methylpropane-2-thiol (83.6 mg, 927 µmol, **13**) in 2 mL of dry dimethylformamide was added and stirred for 19 h at 100 °C. The mixture was diluted with deionized water (300 mL), ethyl acetate (100 mL) and a saturated solution of sodium chloride (200 mL) and the layers were separated. The aqueous layer was extracted with ethyl acetate (2 × 100 mL) and the combined organic layers were washed with a sodium chloride solution (4 × 500 mL). The organic layer was dried over magnesium sulfate and the crude product was applied on silica and purified by flash column chromatography

(Biotage, SNAP Ultra cartridge HP-Sphere 10 g, cyclohexane/ethyl acetate 15.67:1 → 1:1, R_f (3:1) = 0.27). The product was obtained as an orange oil.

Yield: 231 mg (660 µmol, 79%)

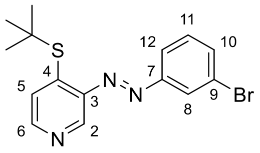

M.p.: 77 °C

^{1}H NMR (500 MHz, $CDCl_3$, 298 K): δ = 8.68 (s, 1H, *H*-2), 8.53 (d, ^3J = 5.27 Hz, 1H, *H*-5), 8.07 (t, ^4J = 1.87 Hz, 1H, *H*-8), 7.91 (ddd, ^3J = 7.94 Hz, ^4J = 1.76 Hz, ^4J = 1.02 Hz 1H, *H*-12), 7.63 (ddd, ^3J = 7.94 Hz, ^4J = 1.94 Hz, ^4J = 1.03 Hz, 1H, *H*-10), 7.60 (d, ^3J = 5.27 Hz, 1H, *H*-6), 7.42 (t, ^3J = 7.94 Hz, 1H, *H*-11), 1.51 (s, 9H, SCCH_3) ppm.

^{13}C NMR (126 MHz, $CDCl_3$, 298 K): δ = 153.5 (*C*-7), 150.5 (*C*-5), 147.7 (*C*-4), 145.4 (*C*-3), 139.0 (*C*-2), 134.3 (*C*-10), 130.6 (*C*-11), 127.4 (*C*-6), 125.5 (*C*-8), 123.2 (*C*-9), 123.1 (*C*-12), 47.7 (S*C*CH_3), 31.3 (SC*C*H_3) ppm.

EI-MS (70 eV): m/z (%) = 349.05 (1) $[M]^+$, 291.97 (100) $[M-C_4H_9]^+$, 154.96 (16) $[M-C_9H_{12}N_3S]^+$, 110.01 (7) $[M-C_{10}H_{13}BrN_2]^+$.

HR-EI-MS (70 eV): m/z = 349.02483 (calcd. for $C_{15}H_{16}BrN_3S$), 349.02462 (found) (-0.59 ppm).

FT-IR (ATR): ν = 3045 (w), 2987 (w), 2960 (w), 2925 (w), 2867 (w), 1562 (s), 1535 (m), 1456 (s), 1425 (m), 1396 (m), 1368 (m), 1297 (w), 1269 (w), 1242 (w), 1182 (m), 1157 (s), 1088 (s), 1057 (m), 1021 (w), 995 (m), 927 (m), 895 (m), 881 (m), 835 (m), 826 (m), 790 (m), 776 (s), 750 (m), 741 (m), 732 (s), 720 (s), 688 (m), 670 (s), 656 (s) cm^{-1}.

EA (%): calcd. for $C_{15}H_{16}BrN_3S$: C: 51.43, H: 4.60, N: 12.00, S: 9.15;
Found: C: 52.72, H: 4.91, N: 11.91, S: 9.00.

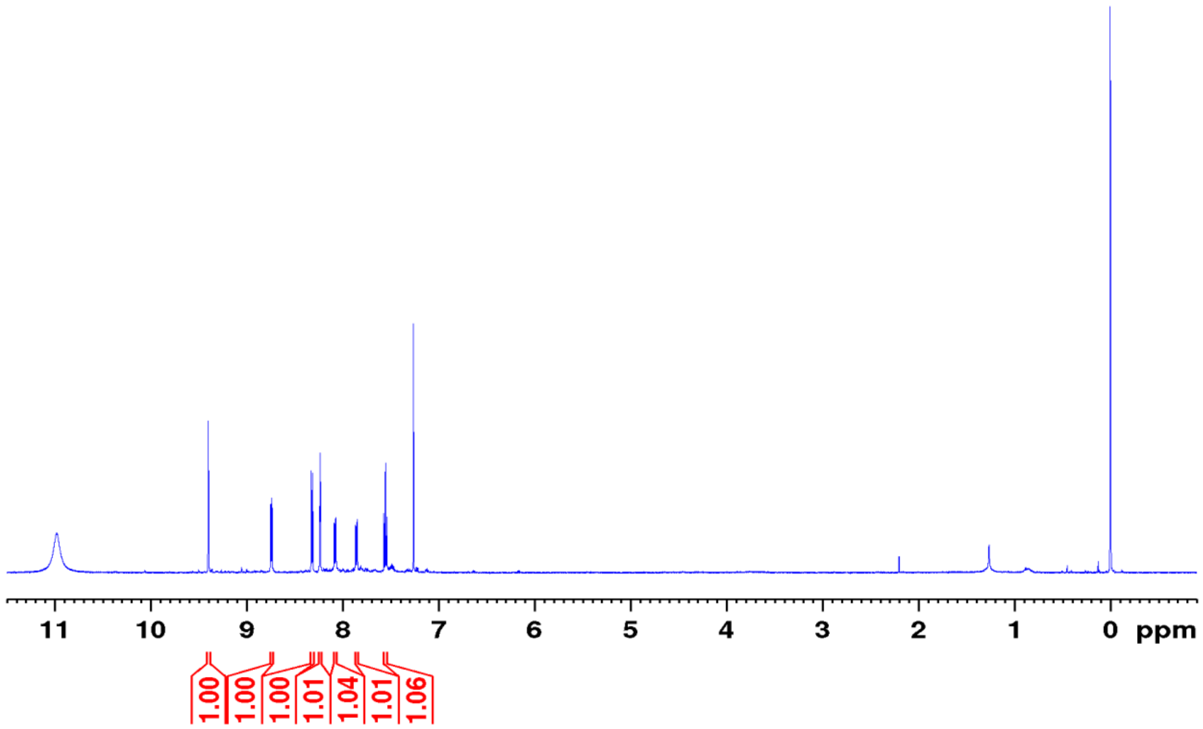

Figure S4.1: Full ^{1}H NMR spectrum of 3-(3-bromophenylazo)-4-*tert*-butylmercaptopyridine (**14**) (500 MHz, $CDCl_3$, 298 K).

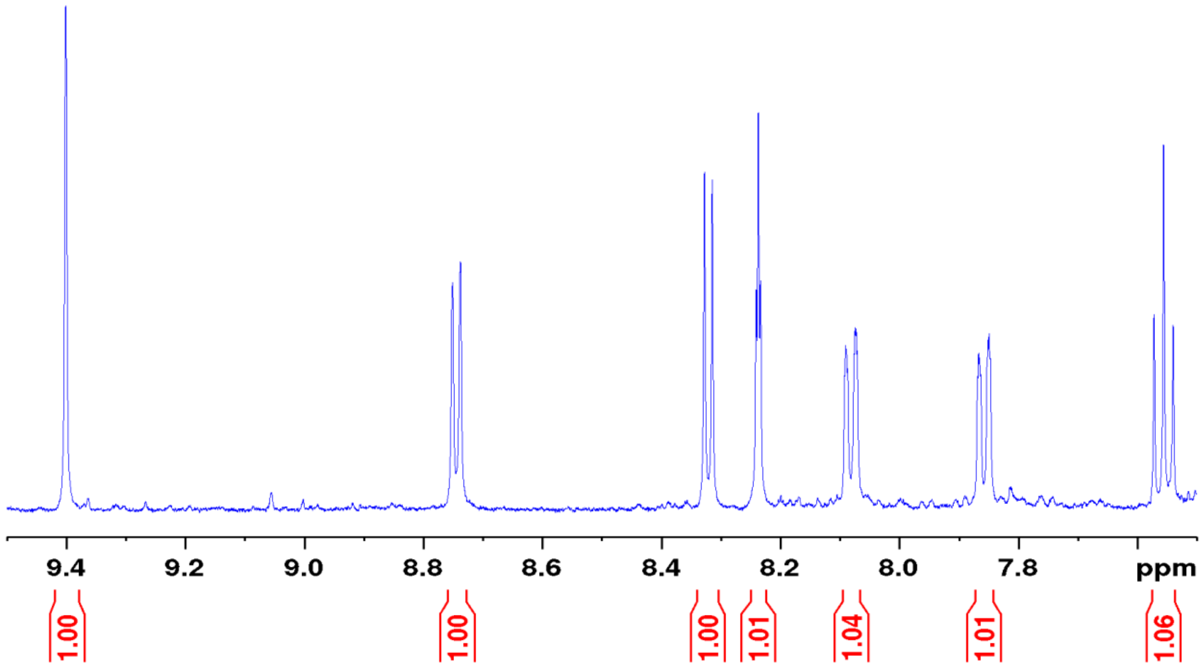

Figure S4.2: Aromatic section of the ^{1}H NMR spectrum of 3-(3-bromophenylazo)-4-*tert*-butylmercaptopyridine (**14**) (500 MHz, $CDCl_3$, 298 K).

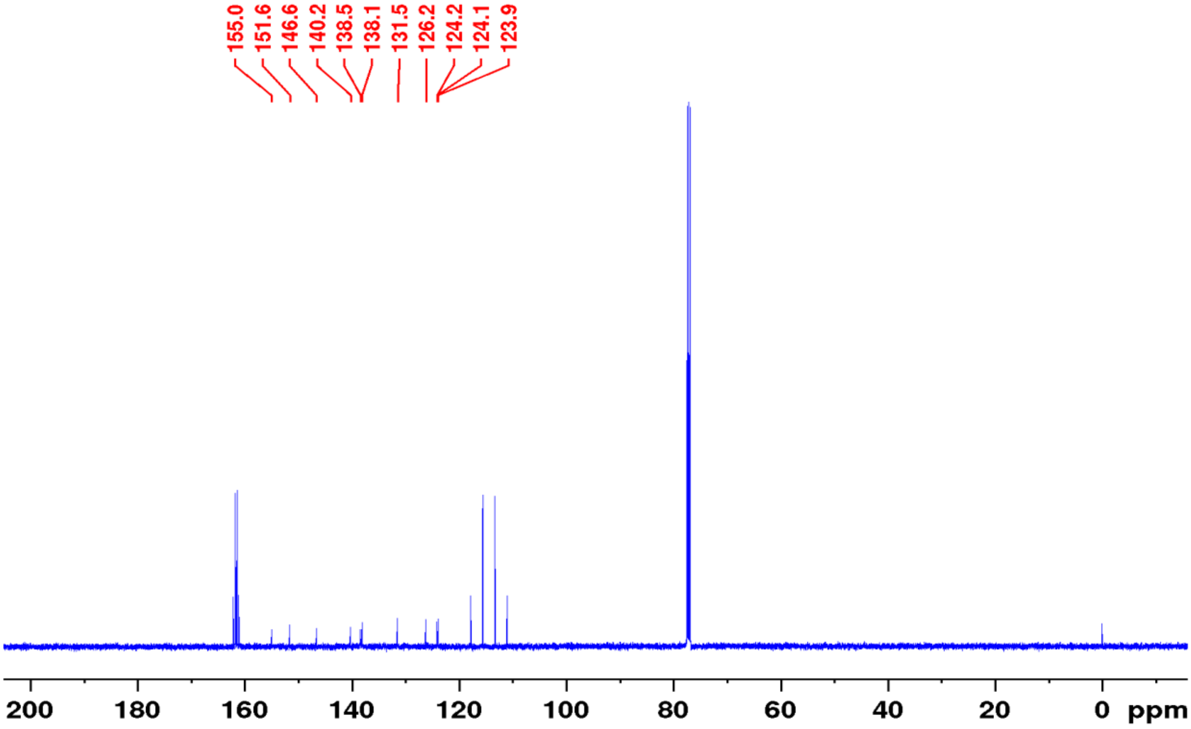

Figure S4.3: Full ^{13}C NMR spectrum of 3-(3-bromophenylazo)-4-*tert*-butylmercaptopyridine (**14**) (126 MHz, $CDCl_3$, 298 K).

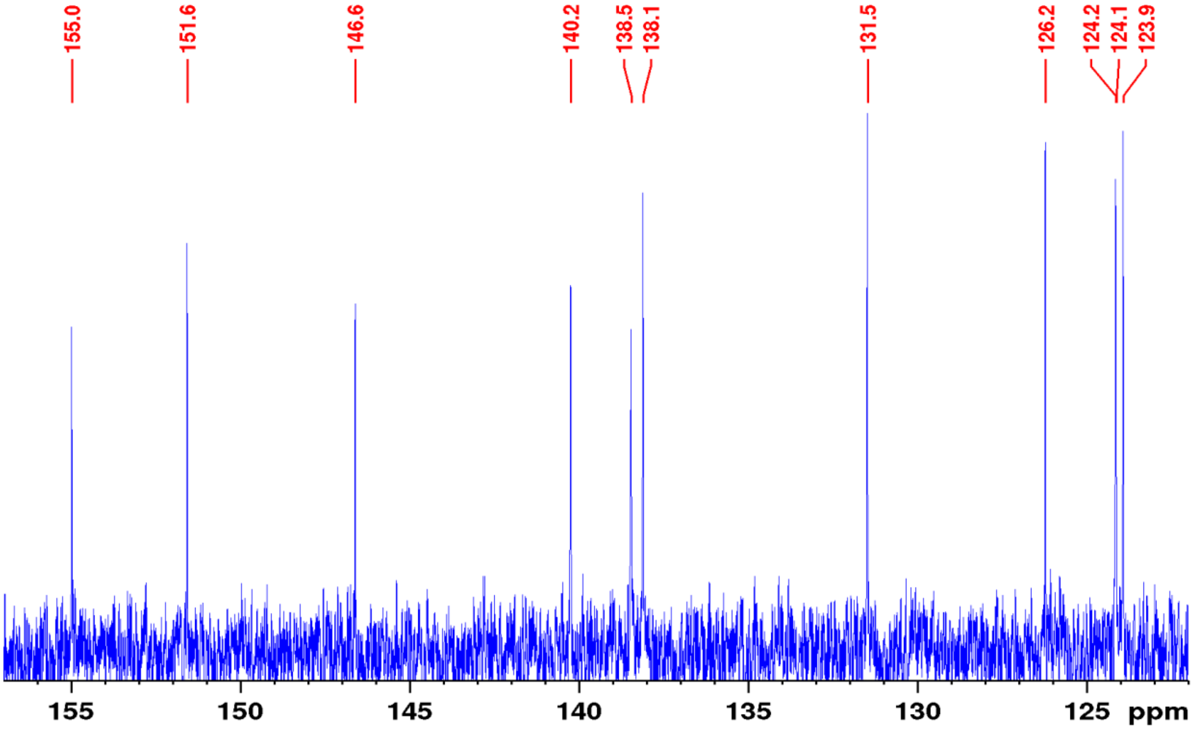

Figure S4.4: Aromatic section of the ^{13}C NMR spectrum of 3-(3-bromophenylazo)-4-*tert*-butylmercaptopyridine (**14**) (126 MHz, $CDCl_3$, 298 K).

Synthesis of 3-(3-iodophenylazo)-4-*tert*-butylmercaptopyridine (18)

In flame dried glassware under an atmosphere of nitrogen 3-(3-iodophenylazo)-4-chloropyridine (150 mg, 437 µmol, **17**) and potassium carbonate (302 mg, 2.19 mmol) were dissolved in 60 mL of dry dimethylformamide. 2-Methylpropane-2-thiol (43.3 mg, 480 µmol, **13**) in 2 mL of dry dimethylformamide was added and stirred for 19 h at 100 °C. The mixture was diluted with deionized water (120 mL), ethyl acetate (60 mL) and a saturated solution of sodium chloride (50 mL) and the layers were separated. The aqueous layer was extracted with ethyl acetate (2 × 60 mL) and the combined organic layers were washed with a sodium chloride solution (4 × 200 mL). The organic layer was dried over magnesium sulfate and the crude product was applied on silica and purified by flash column chromatography (Biotage, SNAP Ultra cartridge HP-Sphere 50 g, cyclohexane/ethyl acetate 15.67:1 → 1:1, R_f (3:1) = 0.23). The product was obtained as an orange solid.

Yield: 134 mg (337 µmol, 77%)

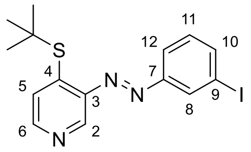

M.p.: 107 °C

^{1}H NMR (600 MHz, $CDCl_3$, 298 K): δ = 8.68 (s, 1H, *H*-2), 8.53 (d, ^3J = 4.90 Hz, 1H, *H*-5), 8.27 (t, ^4J = 1.73 Hz, 1H, *H*-8), 7.94 (ddd, ^3J = 7.96 Hz, ^4J = 1.44 Hz, ^4J = 0.88 Hz 1H, *H*-12), 7.83 (ddd, ^3J = 7.95 Hz, ^4J = 1.44 Hz, ^4J = 0.89 Hz, 1H, *H*-10), 7.61 (d, ^3J = 4.90 Hz, 1H, *H*-6), 7.28 (t, ^3J = 7.94 Hz, 1H, *H*-11), 1.52 (s, 9H, $SCCH_3$) ppm.

^{13}C NMR (126 MHz, $CDCl_3$, 298 K): δ = 153.4 (*C*-7), 150.2 (*C*-5), 147.7 (*C*-4), 145.8 (*C*-3), 140.3 (*C*-10), 138.8 (*C*-2), 131.7 (*C*-8), 130.8 (*C*-11), 127.4 (*C*-6), 123.5 (*C*-12), 94.6 (*C*-9), 47.8 (S*C*CH$_3$), 31.3 (SC*C*H$_3$) ppm.

EI-MS (70 eV): m/z (%) = 397.00 (1) $[M]^+$, 339.93 (100) $[M{-}C_4H_9]^+$, 202.93 (10) $[M{-}C_9H_{12}N_3S]^+$, 110.01 (3) $[M{-}C_{10}H_{13}IN_2]^+$.

HR-EI-MS (70 eV): m/z = 397.00252 (calcd. for $C_{15}H_{15}IN_3S$), 397.00170 (found) (-0.83 ppm).

FT-IR (ATR): ν = 3081 (w), 2964 (w), 2923 (w), 1560 (s), 1532 (m), 1458 (s), 1397 (m), 1370 (m), 1292 (w), 1270 (w), 1188 (m), 1154 (s), 1087 (s), 1054 (m), 993 (m), 976 (w), 935 (m), 922 (m), 899 (m), 881 (m), 824 (s), 784 (s), 739 (s), 715 (s), 684 (m), 671 (s), 649 (m) cm^{-1}.

EA (%): calcd. for $C_{15}H_{16}IN_3S$: C: 45.35, H: 4.06, N: 10.58, S: 8.07; Found: C: 45.94, H: 4.09, N: 10.66, S: 8.04.

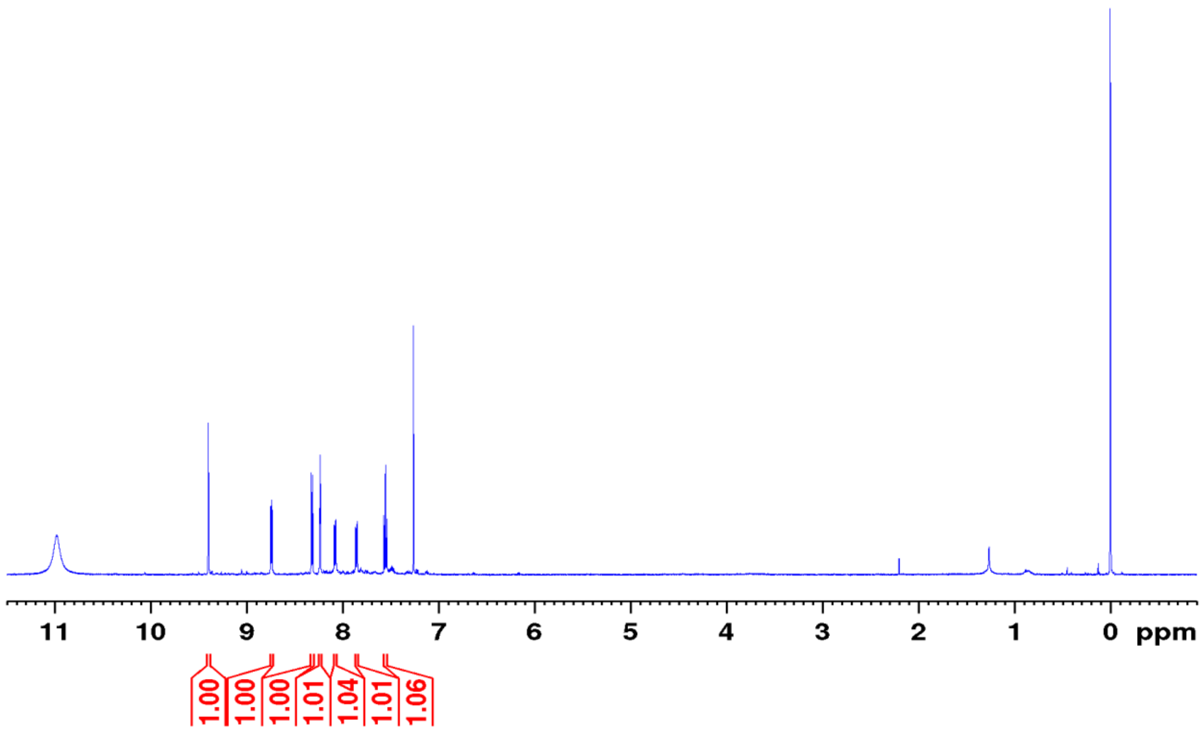

Figure S5.1: Full ^{1}H NMR spectrum of 3-(3-iodophenylazo)-4-*tert*-butylmercaptopyridine (**18**) (600 MHz, $CDCl_3$, 298 K).

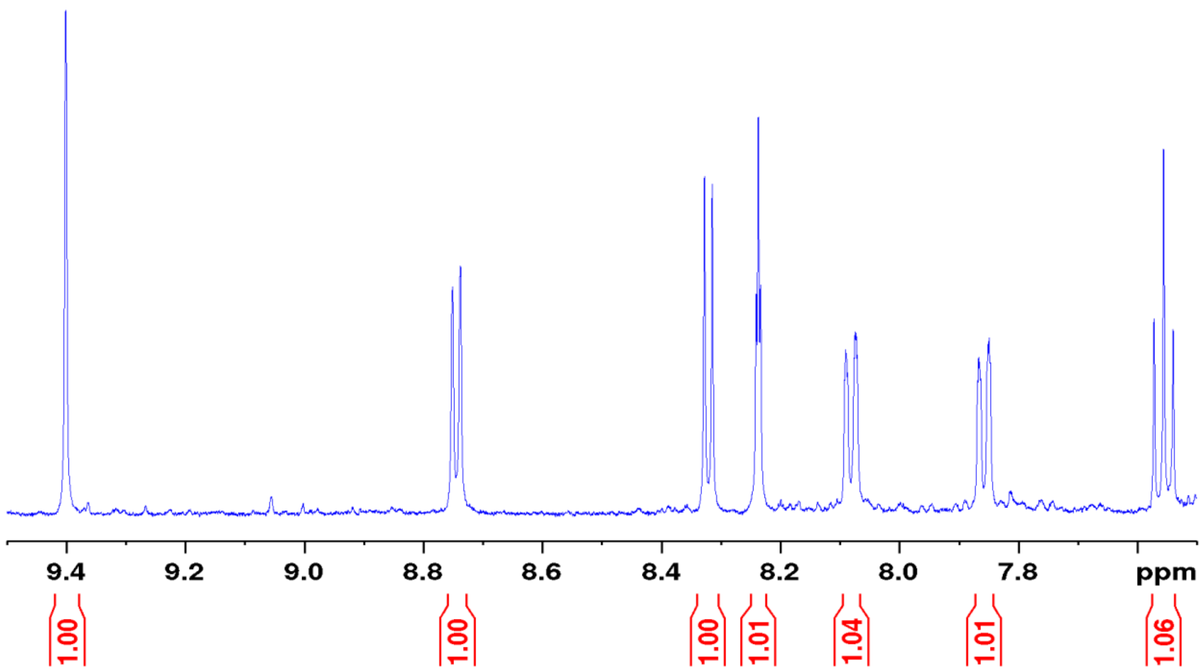

Figure S5.2: Aromatic section of the ^{1}H NMR spectrum of 3-(3-iodophenylazo)-4-*tert*-butylmercaptopyridine (**18**) (600 MHz, $CDCl_3$, 298 K).

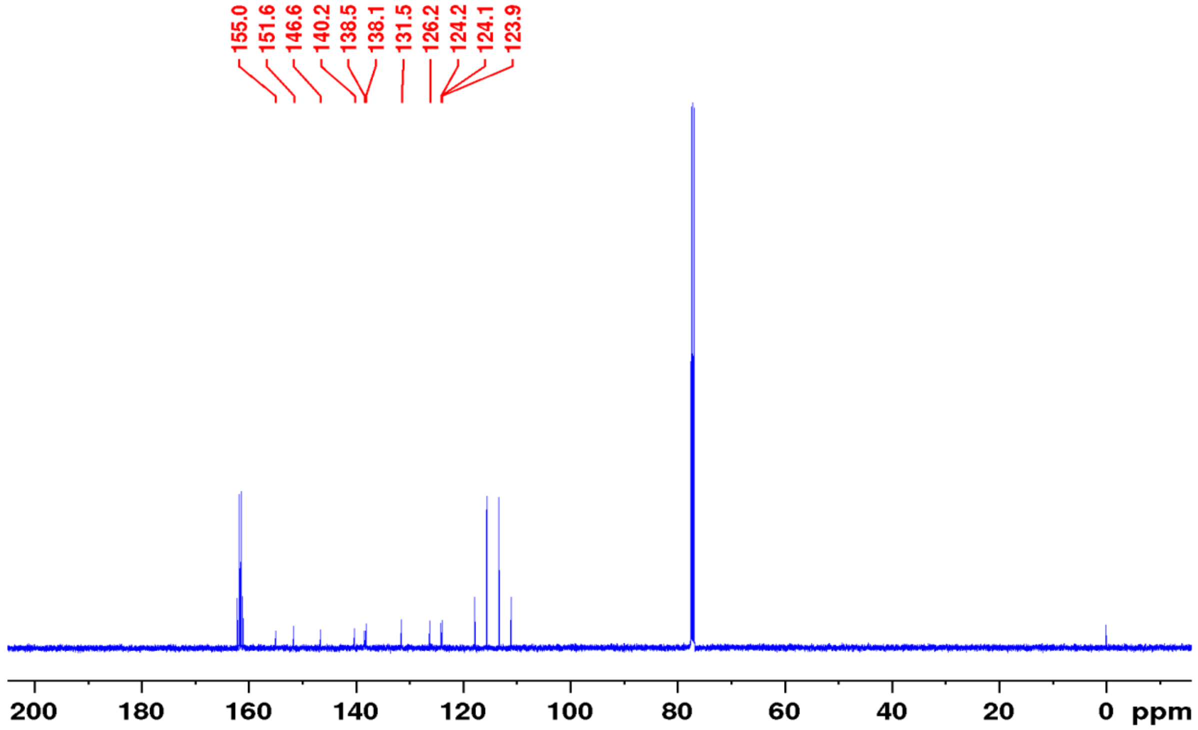

Figure S5.3: Full ^{13}C NMR spectrum of 3-(3-iodophenylazo)-4-*tert*-butylmercaptopyridine (**18**) (126 MHz, $CDCl_3$, 298 K).

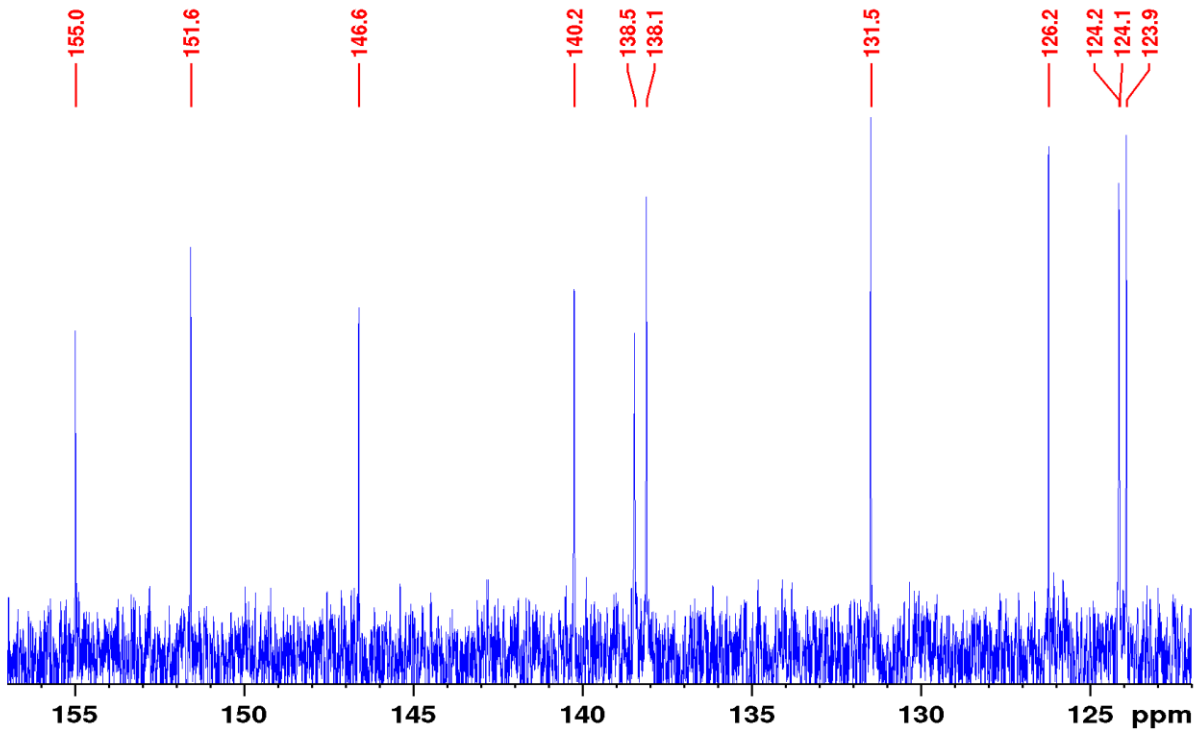

Figure S5.4: Aromatic section of the ^{13}C NMR spectrum of 3-(3-iodophenylazo)-4-*tert*-butylmercaptopyridine (**18**) (126 MHz, $CDCl_3$, 298 K).

Synthesis of methyl (*E*)-3-((3-((3-bromophenyl)diazenyl)pyridin-4-yl)thio)propanoate (16)

In flame dried glassware under an atmosphere of nitrogen methyl-3-mercaptopropionate (487 mg, 4.05 mmol, **15**) and sodium methoxide (0.75 mL 5.4 M in methanol, 4.05 mmol) were dissolved in 35 mL of dry dimethylformamide and cooled to 0 °C. 3-(3-bromophenylazo)-4-chloropyridine (1.00 g, 3.37 mmol, **10**) in 25 mL of dry dimethylformamide was added and stirred at 0 °C for 1 h. The mixture was poured in ice-cold water (200 mL), dichloromethane (300 mL) and a saturated solution of sodium chloride (150 mL). The aqueous layer was extracted with ethyl acetate (6 × 100 mL), the combined organic layers were reduced to 100 mL in vacuo and washed with deionized water (5 × 75 mL). The organic layer was dried over magnesium sulfate and the solvent was removed in vacuo. The crude product was

applied on silica and purified by column chromatography (silica, ethyl acetate, R_f (ethyl acetate) = 0.45). The product was obtained as an orange solid.

Yield: 647 mg (1.70 mmol, 50%)

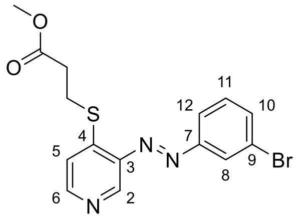

M.p.: 115 °C

^{1}H NMR (500 MHz, $CDCl_3$, 298 K): δ = 9.17 (s, 1H, *H*-2-*cis*), 8.84 (s, 1H, *H*-2-*trans*), 8.48 (d, ^3J = 5.48 Hz, 1H, *H*-5-*trans*), 8.47 (d, ^3J = 5.50 Hz, 1H, *H*-5-*cis*), 8.17 (t, ^4J = 1.82 Hz, 1H, *H*-8-*cis*), 8.06 (t, ^4J = 1.86 Hz, 1H, *H*-8-*trans*), 8.00 (ddd, ^3J = 8.00 Hz, ^4J = 1.82 Hz, ^4J = 1.00 Hz 1H, *H*-12-*cis*), 7.91 (ddd, ^3J = 7.94 Hz, ^4J = 1.76 Hz, ^4J = 1.02 Hz 1H, *H*-12-*trans*), 7.70-7.67 (m, 1H, *H*-10-*cis*), 7.68 (d, ^3J = 5.50 Hz, 1H, *H*-6-*cis*), 7.63 (ddd, ^3J = 7.94 Hz, ^4J = 1.94 Hz, ^4J = 1.03 Hz, 1H, *H*-10-*trans*), 7.46 (t, ^3J = 8.00 Hz, 1H, *H*-11-*cis*), 7.42 (t, ^3J = 7.94 Hz, 1H, *H*-11-*trans*), 7.60 (d, ^3J = 5.48 Hz, 1H, *H*-6-*trans*), 3.75 (s, 3H, $COOCH_3$), 3.30 (t, ^3J = 7.53 Hz, 2H, SCH_2CH_2), 2.80 (t, ^3J = 7.53 Hz, 2H, SCH_2CH_2) ppm.

^{13}C NMR (126 MHz, $CDCl_3$, 298 K): δ = 171.7 (*C*OO), 153.2 (*C*-7), 150.6 (*C*-5), 147.4 (*C*-3), 144.0 (*C*-4), 142.1 (*C*-2), 134.3 (*C*-10), 130.6 (*C*-11), 125.1 (*C*-8), 123.2 (*C*-9), 123.0 (*C*-12), 119.1 (*C*-6), 52.2 ($COOCH_3$), 32.8 (SCH_2CH_2), 25.7 (SCH_2CH_2) ppm.

EI-MS (70 eV): m/z (%) = 379.00 (1) $[M]^+$, 291.95 (100) $[M-C_4H_7O_2]^+$, 213.04 (2) $[M-C_4H_7O_2Br]^+$, 154.95 (16) $[M-C_9H_{10}N_3O_2S]^+$, 110.01 (3) $[M-C_{10}H_{11}BrN_2O_2]^+$.

HR-EI-MS (70 eV): m/z = 378.99901 (calcd. for $C_{15}H_{14}BrN_3O_2S$), 378.99947 (found) (1.22 ppm).

FT-IR (ATR): ν = 3083 (w), 3059 (w), 3005 (w), 2951 (w), 1728 (s), 1567 (s), 1537 (m), 1475 (m), 1458 (m), 1433 (m), 1408 (s), 1369 (s), 1297 (m), 1274 (w), 1219 (s),

1198 (s), 1169 (m), 1148 (s), 1118 (m), 1092 (m), 1058 (m), 996 (m), 971 (m), 945 (m), 930 (m), 908 (m), 889 (m), 851 (m), 832 (s), 788 (s), 728 (s), 702 (m), 676 (s), 656 (m) cm^{-1}.

EA (%): calcd. for $C_{15}H_{14}BrN_3O_2S$: C: 47.38, H: 3.71, N: 11.05, S: 8.43;

Found: C: 47.31, H: 3.56, N: 11.38, S: 8.40.

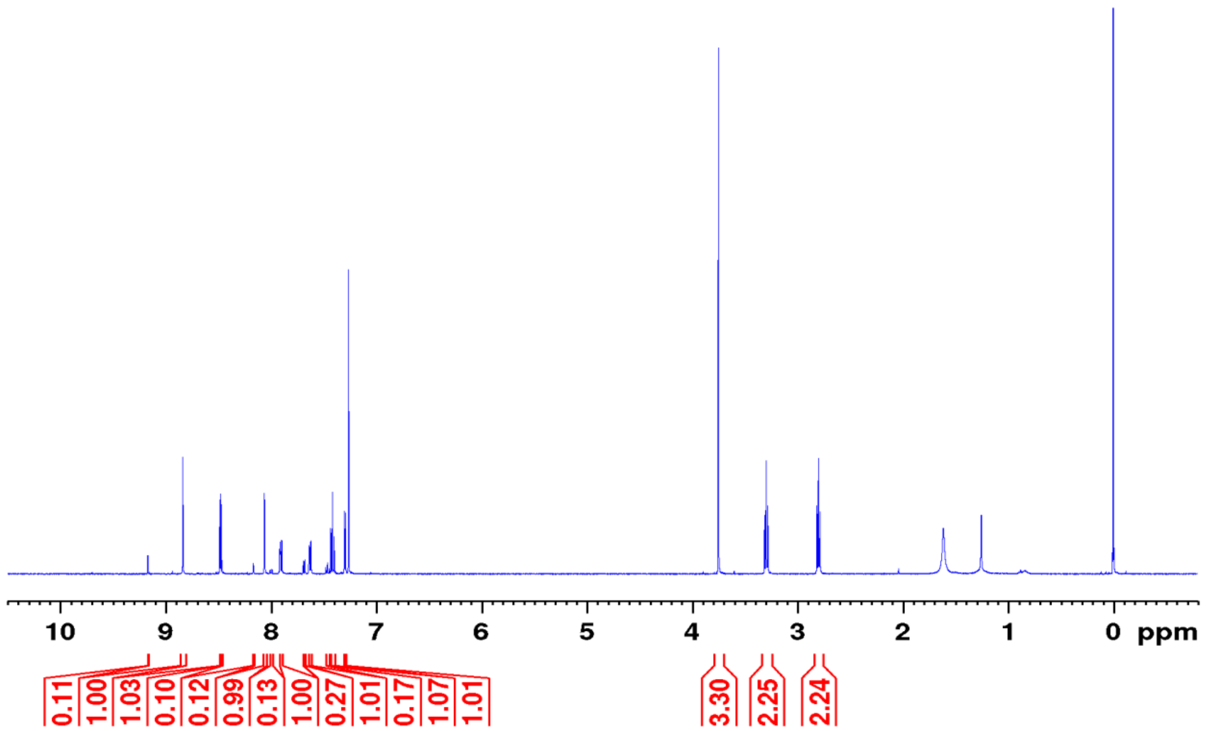

Figure S6.1: Full ^{1}H NMR spectrum of methyl (*E*)-3-((3-((3-bromophenyl)diazenyl)pyridin-4-yl)thio)propanoate (**16**) (500 MHz, $CDCl_3$, 298 K).

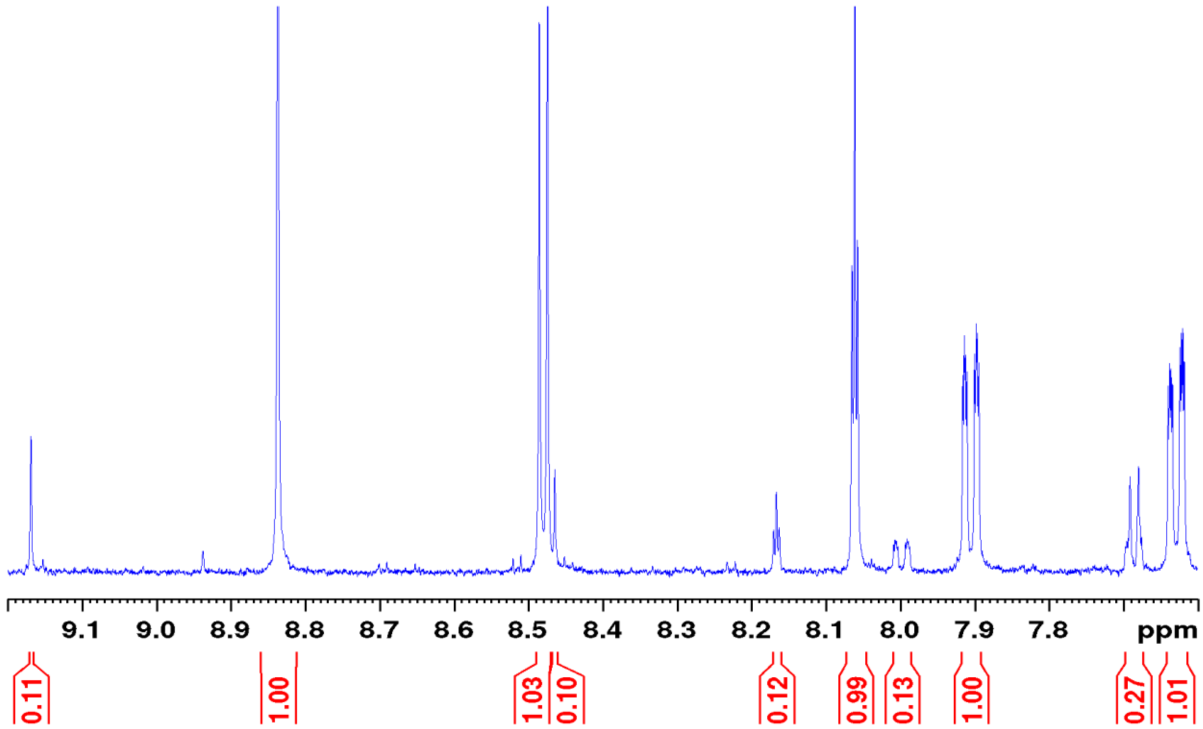

Figure S6.2: Aromatic section of the ^{1}H NMR spectrum of methyl (*E*)-3-((3-((3-bromophenyl)diazenyl)pyridin-4-yl)thio)propanoate (**16**) (500 MHz, $CDCl_3$, 298 K).

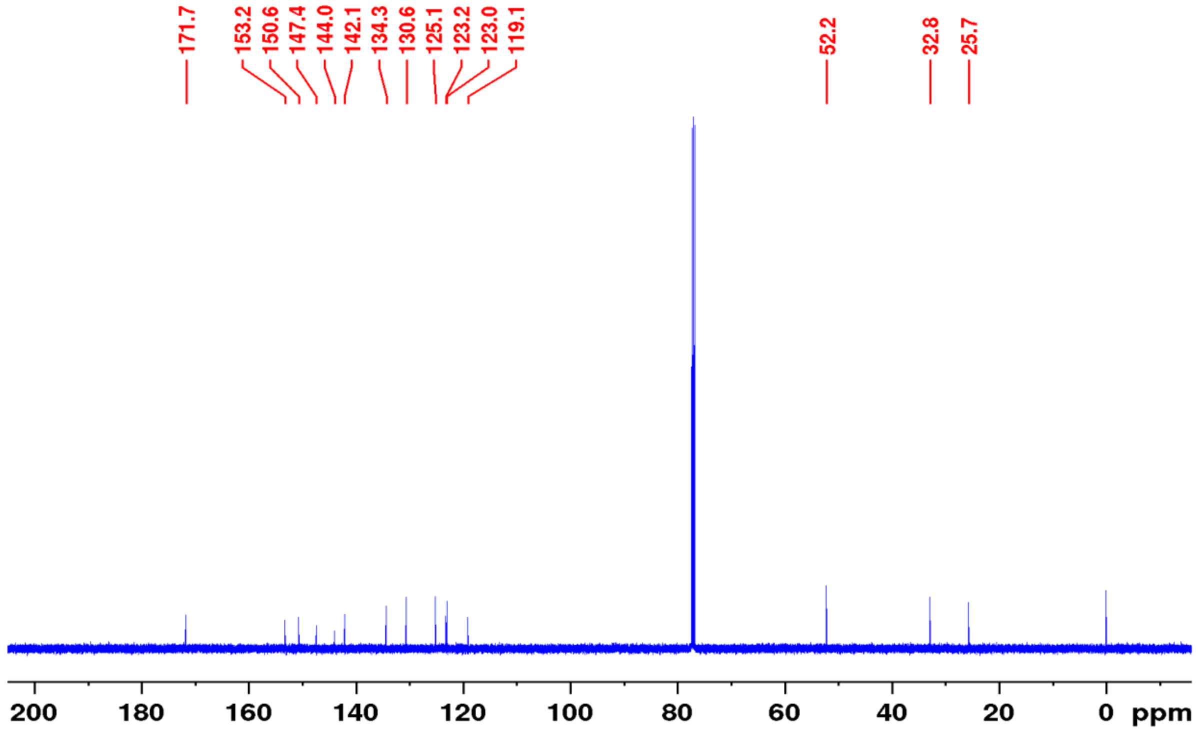

Figure S6.3: Full ^{13}C NMR spectrum methyl (*E*)-3-((3-((3-bromophenyl)diazenyl)pyridin-4-yl)thio)propanoate (**16**) (126 MHz, $CDCl_3$, 298 K).

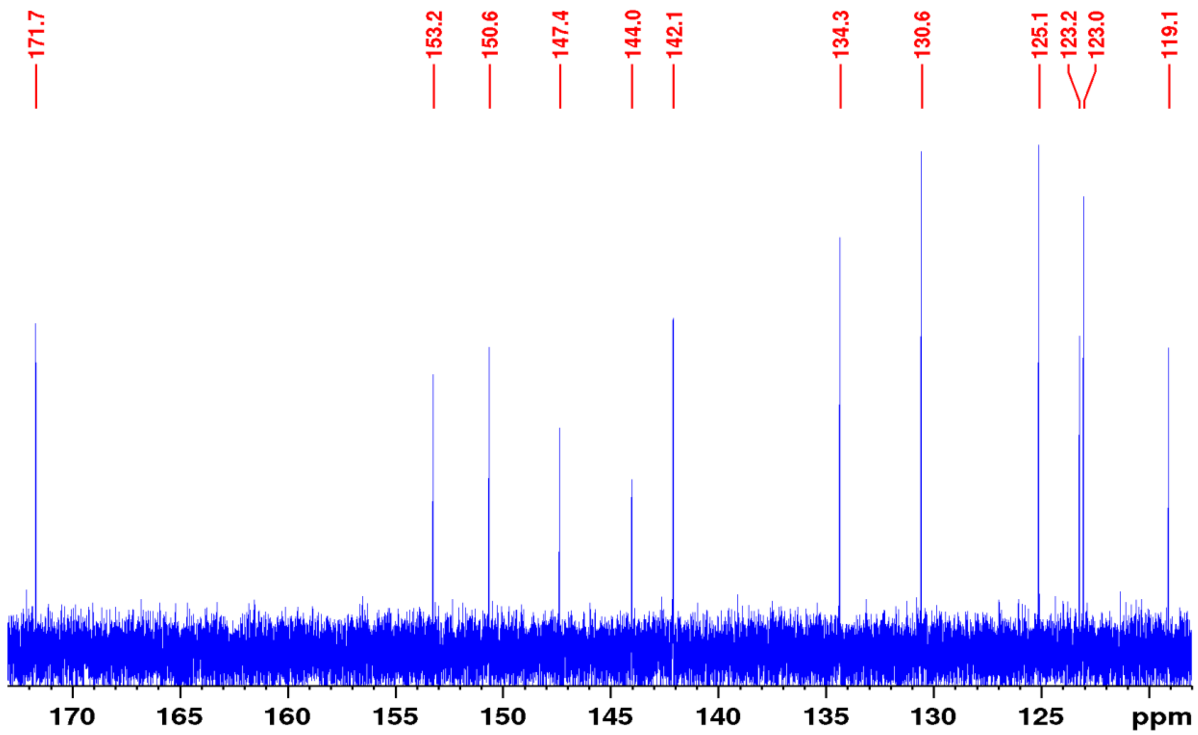

Figure S6.4: Aromatic section of the ^{13}C NMR spectrum of methyl (*E*)-3-((3-((3-bromophenyl)diazenyl)pyridin-4-yl)thio)propanoate (**16**) (126 MHz, $CDCl_3$, 298 K).

Synthesis of 3-(3-bromophenylazo)-4-cyanopyridine (20)

3-Bromoaniline (7.50 g, 43.6 mmol, **2**) was dissolved in 150 mL of dichloromethane and Oxone™ (66.3 g, 215 mmol) in 375 mL deion. water was added. The mixture was stirred at ambient temperature for 4 h. Layers were separated and the aqueous layer was extracted with dichloromethane (2 × 50 mL). The combined org. layers were dried over magnesium sulfate, the solvent was reduced in vacuo to about 10 mL and filtered through silica (dichloromethane, R_f = 0.83) to remove unreacted starting material **2** (dichloromethane, R_f = 0.10). Fractions containing the green nitroso compound **3** were reduced *in vacuo* to about 10 mL. Pyridine (195 mL), an aqueous solution of sodium hydroxide (25%, 65 mL) and 3-amino-4-cyanopyridine (2.40 g, 20.1 mmol, **19**) were added and stirred for 6 h at ambient temperature. The mixture was diluted with ethyl acetate (250 mL) and a saturated aqueous solution of sodium

chloride (150 mL). Layers were separated, the aqueous layer was extracted with ethyl acetate (3 × 100 mL) and the solvent was removed in vacuo. The residue was co-distilled with toluene (3 × 400 mL), applied on silica and purified by flash column chromatography (Biotage, SNAP Ultra cartridge HP-Sphere 340 g, cyclohexane/ethyl acetate 15.67:1 → 1.2:1, R_f (3:1) = 0.26). The product was obtained as an orange solid.

Yield: 3.30 g (11.5 mmol, 57%)

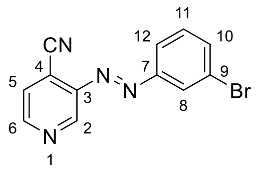

M.p.: 107 °C

^{1}H NMR (500 MHz, $CDCl_3$, 298 K): δ = 9.17 (s, 1H, *H*-2), 8.87 (d, ^3J = 4.97 Hz, 1H, *H*-5), 8.17 (t, ^4J = 1.86 Hz, 1H, *H*-8), 8.01 (ddd, ^3J = 7.95 Hz, ^4J = 1.90 Hz, ^4J = 1.04 Hz 1H, *H*-12), 7.73 (d, ^3J = 4.97 Hz, 1H, *H*-6), 7.71 (ddd, ^3J = 7.95 Hz, ^4J = 1.90 Hz, ^4J = 1.04 Hz, 1H, *H*-10), 7.47 (t, ^3J = 7.95 Hz, 1H, *H*-11) ppm.

^{13}C NMR (126 MHz, $CDCl_3$, 298 K): δ = 152.9 (*C*-7), 151.9 (*C*-5), 145.8 (*C*-4), 141.8 (*C*-2), 135.8 (*C*-10), 130.8 (*C*-11), 126.5 (*C*-6), 126.0 (*C*-8), 123.4 (*C*-9), 123.3 (*C*-12), 118.1 (*C*-3), 114.7 (*C*N) ppm.

EI-MS (70 eV): m/z (%) = 285.99 (23) $[M]^+$, 207.07 (3) $[M{-}Br]^+$, 182.96 (41) $[M\text{-}C_6H_3N_2]^+$, 154.95 (100) $[M{-}C_6H_3N_4]^+$, 103.03 (32) $[M{-}C_6H_4BrN_2]^+$.

HR-EI-MS (70 eV): m/z = 287.98336 (calcd. for $C_{12}H_7BrN_4$), 287.98470 (found) (4.63 ppm).

FT-IR (ATR): ν = 3076 (w), 3056 (w), 2237 (w), 1952 (w), 1580 (s), 1552 (m), 1475 (m), 1449 (w), 1415 (m), 1398 (s), 1305 (w), 1249 (m), 1222 (w), 1196 (m), 1178 (m), 1141 (m), 1091 (m), 1063 (m), 1054 (m), 1026 (w), 992 (w), 979 (w), 939 (w), 918 (w), 894 (s), 857 (s), 840 (s), 795 (s), 776 (s), 750 (s), 698 (s), 676 (s), 655 (s) cm^{-1}.

EA (%): calcd. for $C_{12}H_7BrN_4$: C: 50.20, H: 2.46, N: 19.51;

Found: C: 50.53, H: 2.41, N: 19.45.

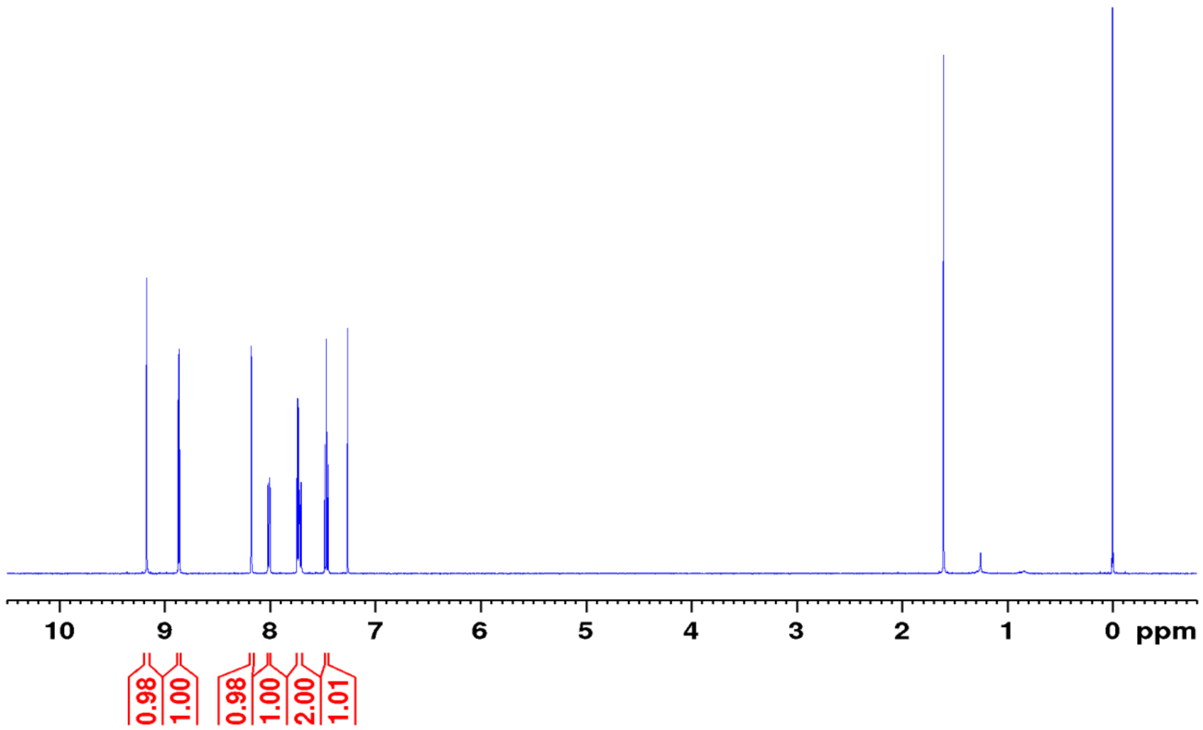

Figure S7.1: Full ^{1}H NMR spectrum of 3-(3-bromophenylazo)-4-cyanopyridine (**20**)

(500 MHz, $CDCl_3$, 298 K).

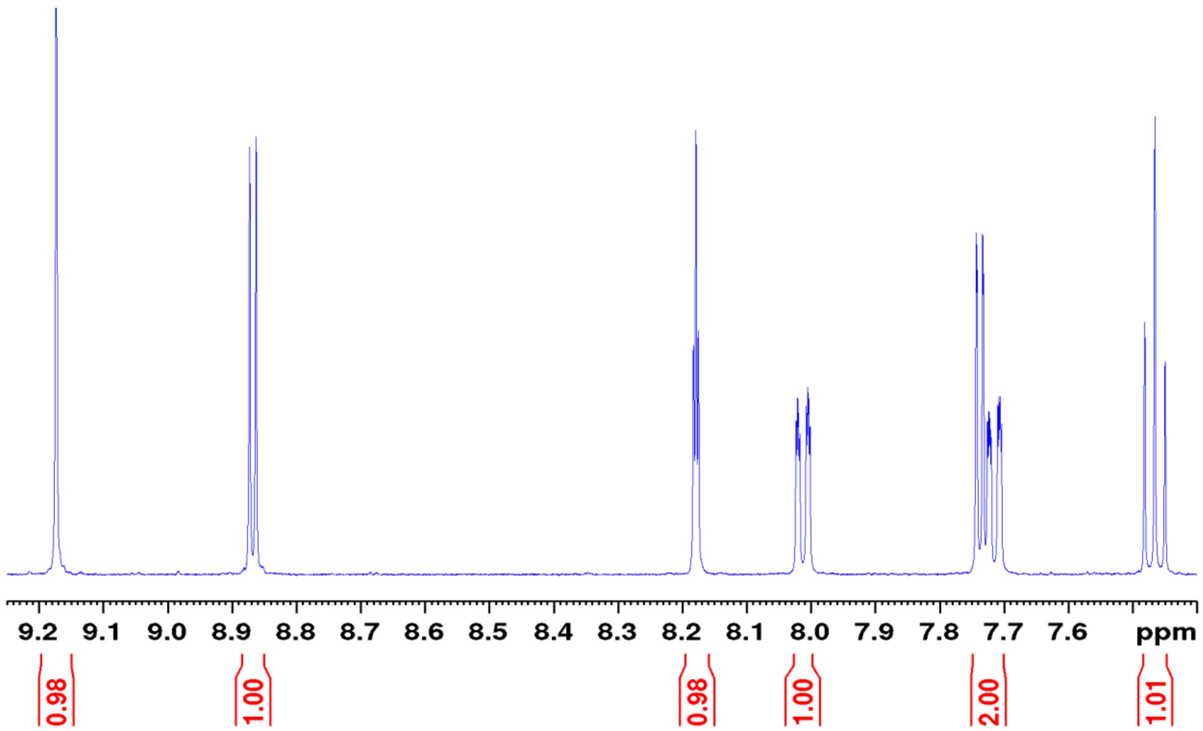

Figure S7.2: Aromatic section of the ^{1}H NMR spectrum of 3-(3-bromophenylazo)-4-cyanopyridine (**20**) (500 MHz, $CDCl_3$, 298 K).

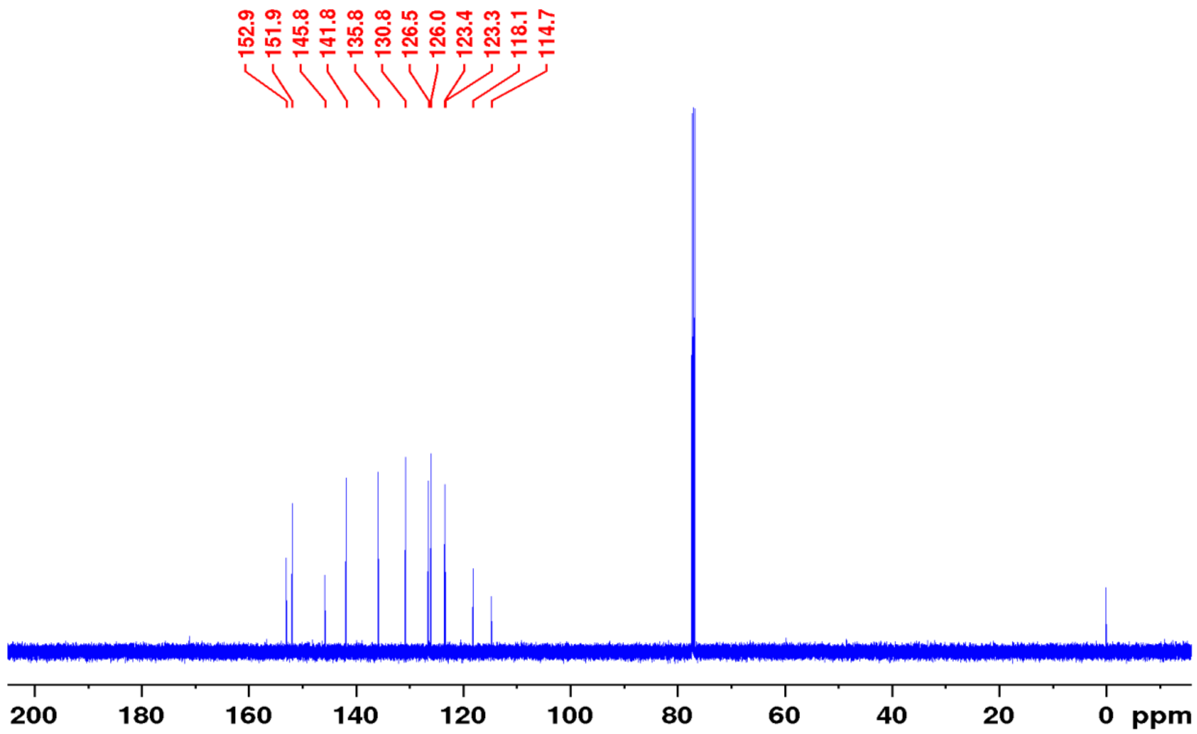

Figure S7.3: Full ^{13}C NMR spectrum of 3-(3-bromophenylazo)-4-cyanopyridine (**20**) (126 MHz, $CDCl_3$, 298 K).

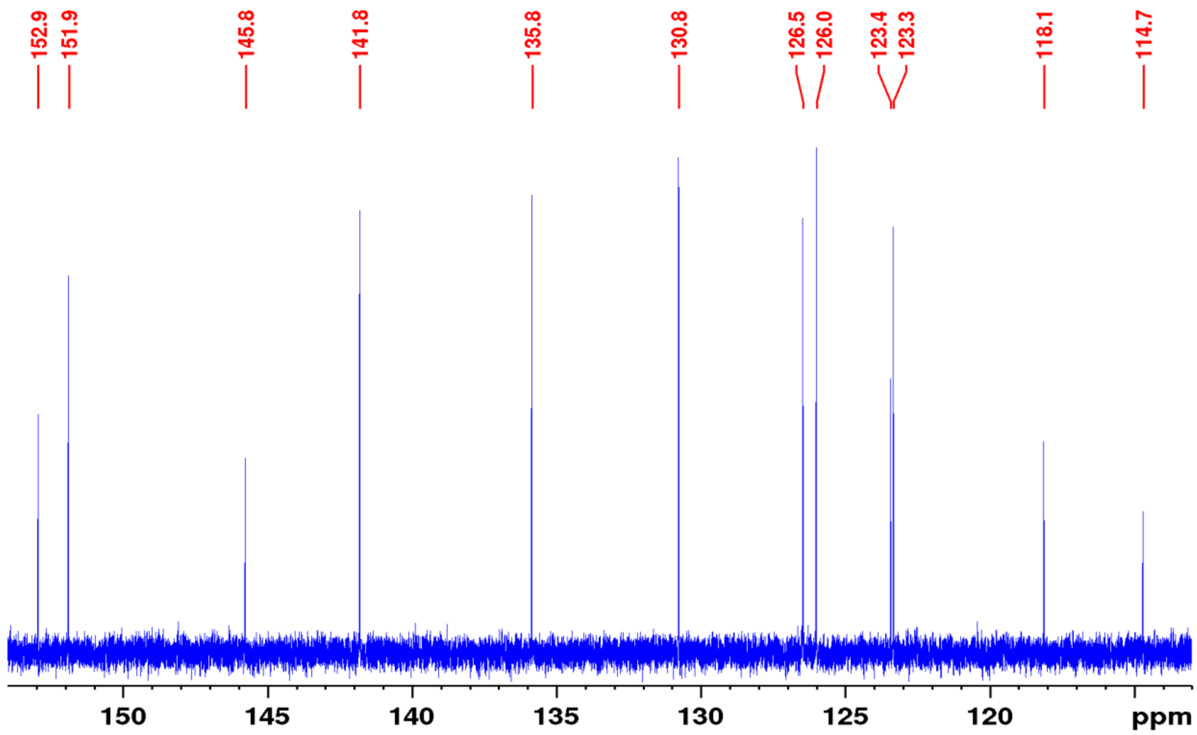

Figure S7.4: Aromatic section of the ^{13}C NMR spectrum of 3-(3-bromophenylazo)-4-cyanopyridine (**20**) (126 MHz, $CDCl_3$, 298 K).

Synthesis of 3-(3-bromophenylazo)-4-pyridinecarboxylic acid (21)

3-(3-bromophenylazo)-4-cyanopyridine (1.00 g, 3.48 mmol, **20**) and sodium hydroxide (1.75 mg, 43.8 mmol) were dissolved in deionized water (8 mL) and 1-butanol (30 mL) and refluxed for 15 h. The mixture was diluted with deionized water (100 mL) and washed with dichloromethane (3 × 100 mL). The aqueous layer was adjusted to pH 5 with glacial acetic acid and extracted with ethyl acetate (2 × 100 mL). The solvent was removed in vacuo and co-distilled with toluene (2 × 150 mL). The product was obtained as a brownish solid.

Yield: 733 mg (2.39 mmol, 68%)

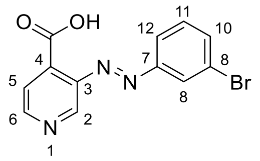

M.p.: 190 °C, decomposition

^{1}H NMR (600 MHz, methanol-d_4, TFA-d_1, 298 K): δ = 8.98 (s, 1H, *H*-2), 8.86 (d, ^3J = 5.25 Hz, 1H, *H*-6), 8.08 (t, ^4J = 1.80 Hz, 1H, *H*-8), 8.00 (d, ^3J = 5.25 Hz, 1H, *H*-5), 7.98 (ddd, ^3J = 7.98 Hz, ^4J = 1.81 Hz, ^4J = 0.88 Hz, 1H, *H*-12), 7.77 (ddd, ^3J = 8.02 Hz, ^4J = 2.04 Hz, ^4J = 0.88 Hz, 1H, *H*-10), 7.55 (t, ^3J = 7.99 Hz, 1H, *H*-11) ppm.

^{13}C NMR (151 MHz, methanol-d_4, TFA-d_1, 298 K): δ = 167.7 (*C*OOH), 154.6 (*C*-7), 149.8 (*C*-6), 147.4 (*C*-3), 140.7 (*C*-4), 140.5 (*C*-2), 136.5 (*C*-12), 132.3 (*C*-11), 126.1 (*C*-8), 125.7 (*C*-5), 124.7 (*C*-10), 124.3 (*C*-9) ppm.

EI-MS (70 eV): m/z (%) = 304.99 (17) $[M]^+$, 182.96 (53) $[M-C_6H_4NO_2]^+$, 154.95 (100) $[M-C_6H_4N_3O_2]^+$, 122.03 (25) $[M-C_6H_4BrN_2]^+$.

HR-EI-MS (70 eV): m/z = 304.97999 (calcd. for $C_{12}H_8BrN_3O_2$), 304.97852 (found) (-4.83 ppm).

FT-IR (ATR): ν = 2923 (w), 2383 (m, br), 1710 (s), 1599 (s), 1574 (s), 1461 (s), 1400 (s), 1297 (s), 1278 (s), 1250 (s), 1177 (s), 1148 (s), 1086 (s), 1055 (s), 996 (s), 942 (s), 918 (s), 890 (s), 858 (s), 790 (s), 777 (s), 711 (s) cm^{-1}.

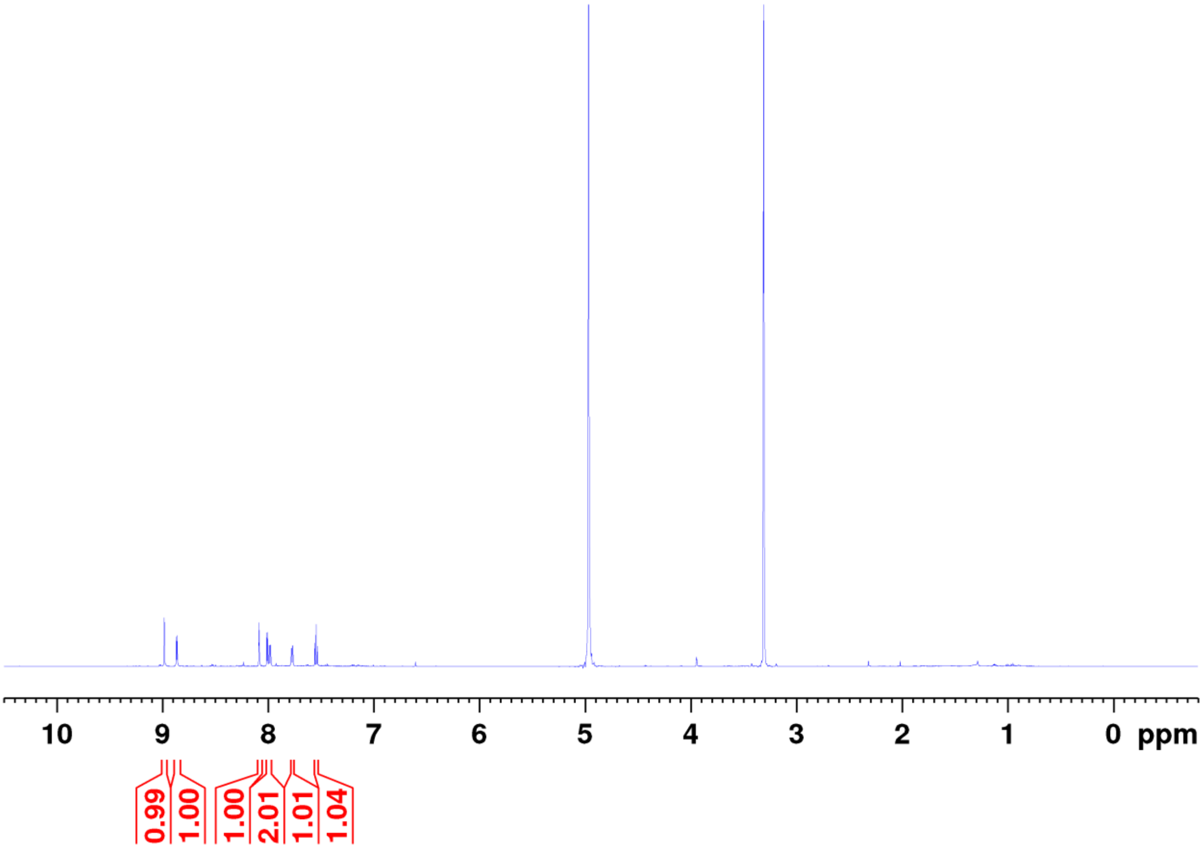

Figure S8.1: Full ^1H NMR spectrum of 3-(3-bromophenylazo)-4-pyridinecarboxylic acid (**21**) (500 MHz, methanol-d_4, TFA-d_1, 298 K).

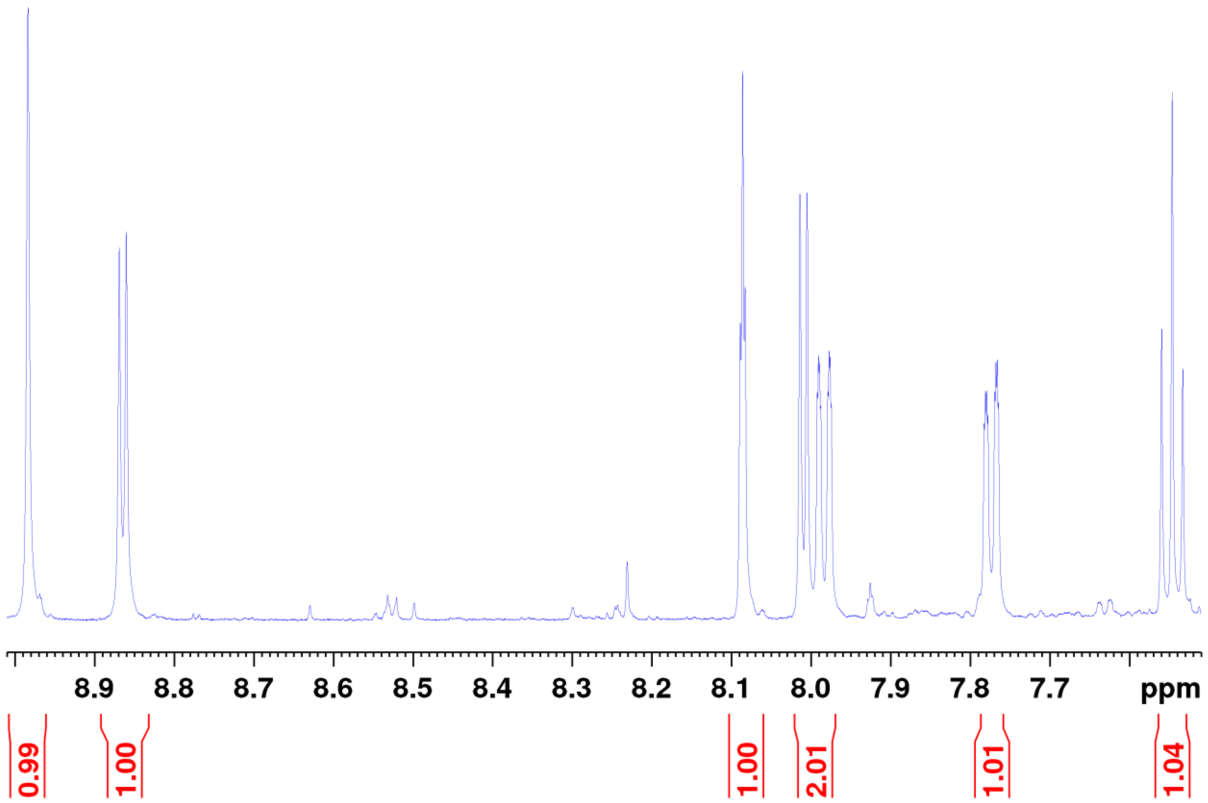

Figure S8.2: Aromatic section of the ^{1}H NMR spectrum of 3-(3-bromophenylazo)-4-pyridinecarboxylic acid (**21**) (500 MHz, methanol-d_4, TFA-d_1, 298 K).

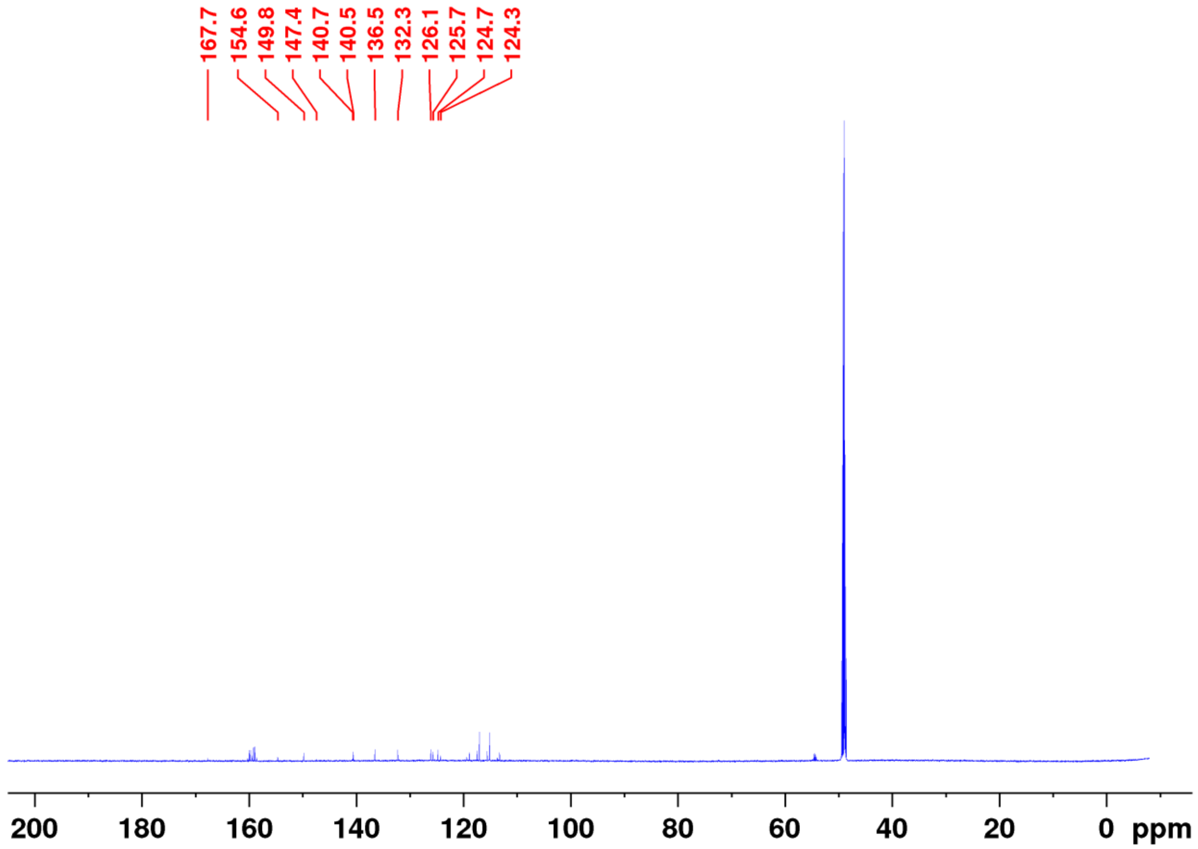

Figure S8.3: Full ^{13}C NMR spectrum of 3-(3-bromophenylazo)-4-pyridinecarboxylic acid (**21**) (126 MHz, methanol-d_4, TFA-d_1, 298 K).

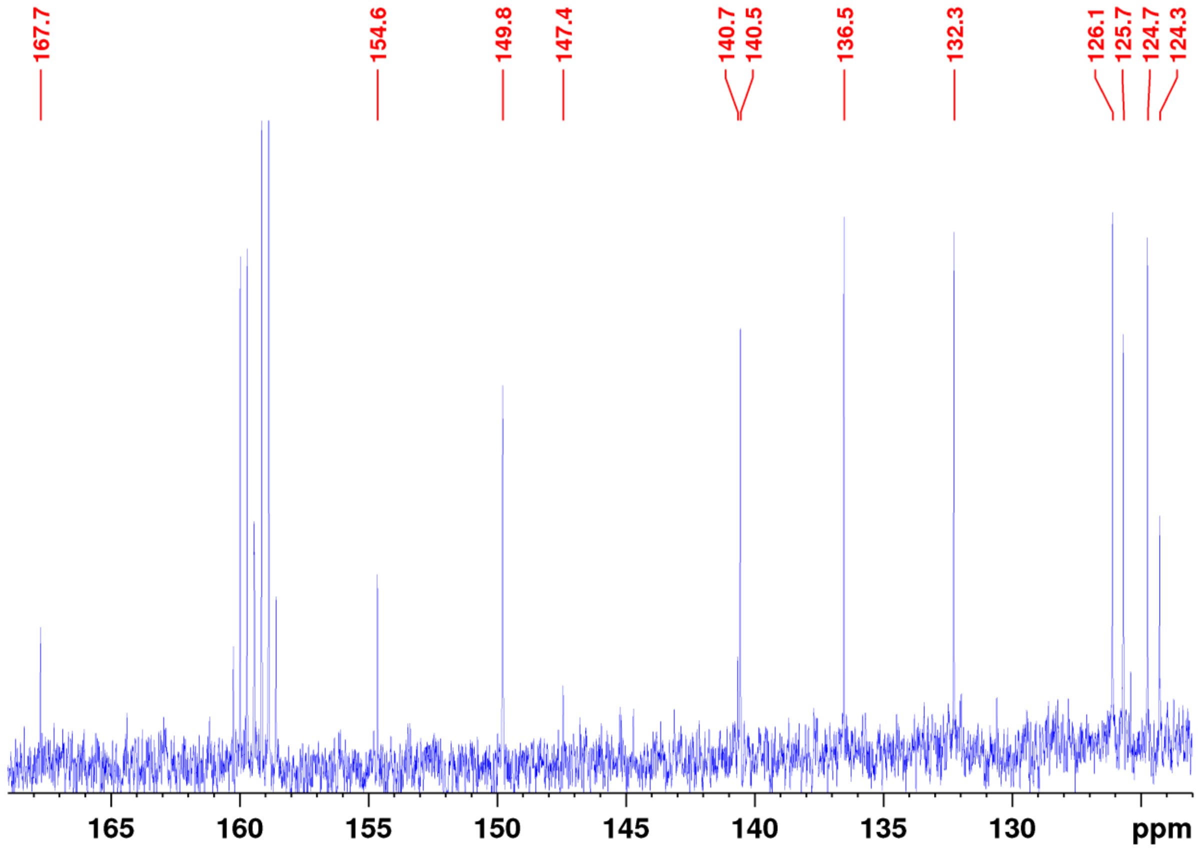

Figure S8.4: Aromatic section of the ^{13}C NMR spectrum of 3-(3-bromophenylazo)-4-pyridinecarboxylic acid (**21**) (126 MHz, methanol-d_4, TFA-d_1, 298 K).

Synthesis of 5-(Biphenylazo-*tert*-butylmercaptopyridine)-10,15,20-tris(pentafluorophenyl)nickel(II)porphyrin (1f)

<u>Synthesis of **1f** with azopyridine **14**:</u>

The solvent (30 mL toluene, 10.5 mL ethanol, 7 mL deionized water) was flushed with nitrogen for 45 min. Porphyrin precursor (191 mg, 179 µmol, **22**), 3-(3-bromophenylazo)-4-*tert*-butylmercaptopyridine (93.8 mg, 268 µmol, **14**), potassium carbonate (81.5 mg, 590 µmol), and tetrakis(triphenylphosphane)-palladium(0) (20.6 mg, 17.8 µmol) were added and stirred for 70 h at 90 °C. The mixture was diluted with a saturated aqueous solution of sodium chloride (80 mL), deionized water (140 mL) and dichloromethane (80 mL). Layers were separated and the aqueous layer was extracted with dichloromethane (3 × 50 mL). The combined

organic layers were dried over magnesium sulfate and the solvent was removed *in vacuo*. The crude product was applied on silica and purified by column chromatography (silica, toluene/ethanol 99.5:0.5, R_f (99.5:0.5) = 0.16). The product was obtained as a red solid.

Yield: 215 mg (178 µmol, 99%)

<u>Synthesis of **1f** with azopyridine **18**:</u>

The solvent (4.4 mL toluene, 1.6 mL ethanol, 1.0 mL deionized water) was flushed with nitrogen for 30 min. Porphyrin precursor (30 mg, 28.1 µmol, **22**), 3-(3-iodophenylazo)-4-*tert*-butylmercaptopyridine (22.3 mg, 56.1 µmol, **18**), potassium carbonate (11.7 mg, 84.7 µmol), and tetrakis(triphenylphosphane)-palladium(0) (3.25 mg, 2.81 µmol) were added and stirred for 15 h at 90 °C. The mixture was diluted with deionized water (10 mL) and dichloromethane (25 mL). Layers were separated and the aqueous layer was extracted with dichloromethane (2 × 25 mL). The combined organic layers were dried over magnesium sulfate and the solvent was removed in vacuo. The crude product was applied on silica and purified by column chromatography (silica, dichloromethane, R_f = 0.13). The product was obtained as a red solid.

Yield: 34 mg (28.1 µmol, 99%)

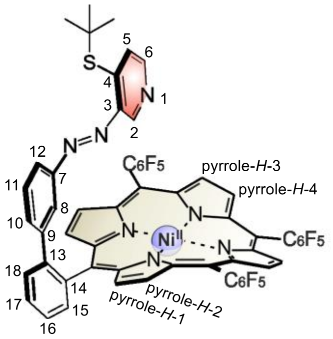

M.p.: 148 °C, decomposed

^{1}H NMR (500 MHz, acetone-d_6, TFA-d_1, 298 K): δ = 9.18 – 9.13 (m, 4H, pyrrole-*H*-3/4), 9.09 – 9.05 (m, 2H, pyrrole-*H*-2), 9.03 – 9.00 (m, 2H, pyrrole-*H*-1), 8.73 (dd, ^{3}J = 6.62 Hz, ^{4}J = 0.73 Hz, 1H, *H*-6), 8.38 (s, 1H, *H*-2), 8.37 (d, ^{4}J = 6.67 Hz, 1H, *H*-5), 8.32 (dd, ^{3}J = 7.57 Hz, ^{4}J = 0.94 Hz, 1H, *H*-15), 8.02 (td, ^{3}J = 7.71 Hz, ^{4}J = 1.35 Hz, 1H, *H*-17), 7.90 (td, ^{3}J = 7.62 Hz, ^{4}J = 1.32 Hz, 1H, *H*-16), 7.89 (dd, ^{3}J = 7.70 Hz, ^{4}J = 0.90 Hz, 1H, *H*-18), 7.70 (t, ^{4}J = 1.81 Hz, 1H, *H*-8), 7.19 (dt, ^{3}J = 7.78 Hz, ^{4}J = 1.35 Hz, 1H, *H*-10), 7.14 (ddd, ^{3}J = 7.94 Hz, ^{4}J = 1.92 Hz, ^{4}J = 1.03 Hz, 1H, *H*-12), 6.77 (t, ^{3}J = 7.95 Hz, 1H, *H*-11) ppm.

^{19}F NMR (471 MHz, acetone-d_6, 298 K): δ = -138.3 - -139.3 (m, 6F, *o*-*F*), -154.6 - -154.9 (m, 3F, *p*-*F*), -163.1 - -163.7 (m, 6F, *m*-*F*) ppm.

HR-ESI-MS: m/z = 1210.1310 (calcd. for $C_{59}H_{29}F_{15}N_7NiS$), 1210.13136 (found) (-0.33 ppm).

MALDI-MS (Cl-CCA): m/z = 1210.5 $[M]^+$.

FT-IR (ATR): ν = 2965 (w), 1651 (w), 1584 (w), 1561 (w), 1518 (s), 1487 (s), 1395 (m), 1342 (m), 1162 (m), 1072 (m), 1060 (m), 986 (s), 956 (m), 938 (s), 927 (m), 838 (m), 800 (m), 762 (s), 741 (m), 703 (m), 695 (m), 658 (w) cm^{-1}.

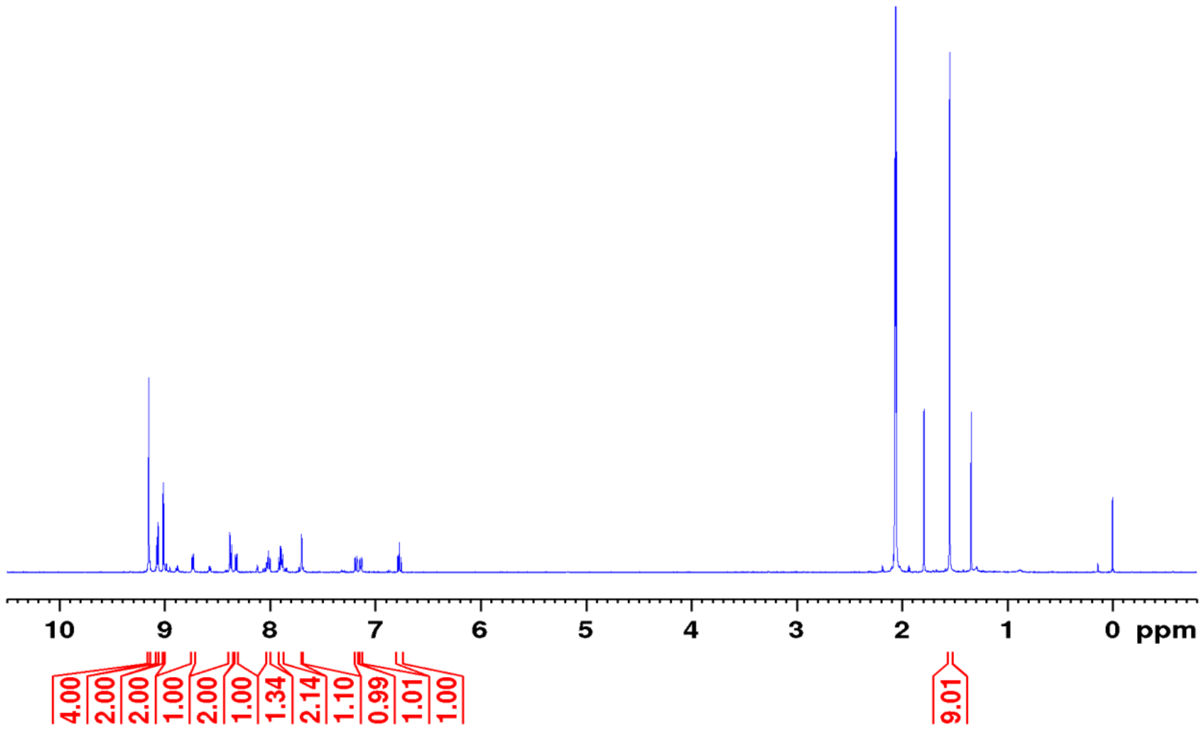

Figure S9.1: Full ^{1}H NMR spectrum of **1f** (500 MHz, acetone-d_6, TFA-d_1, 298 K).

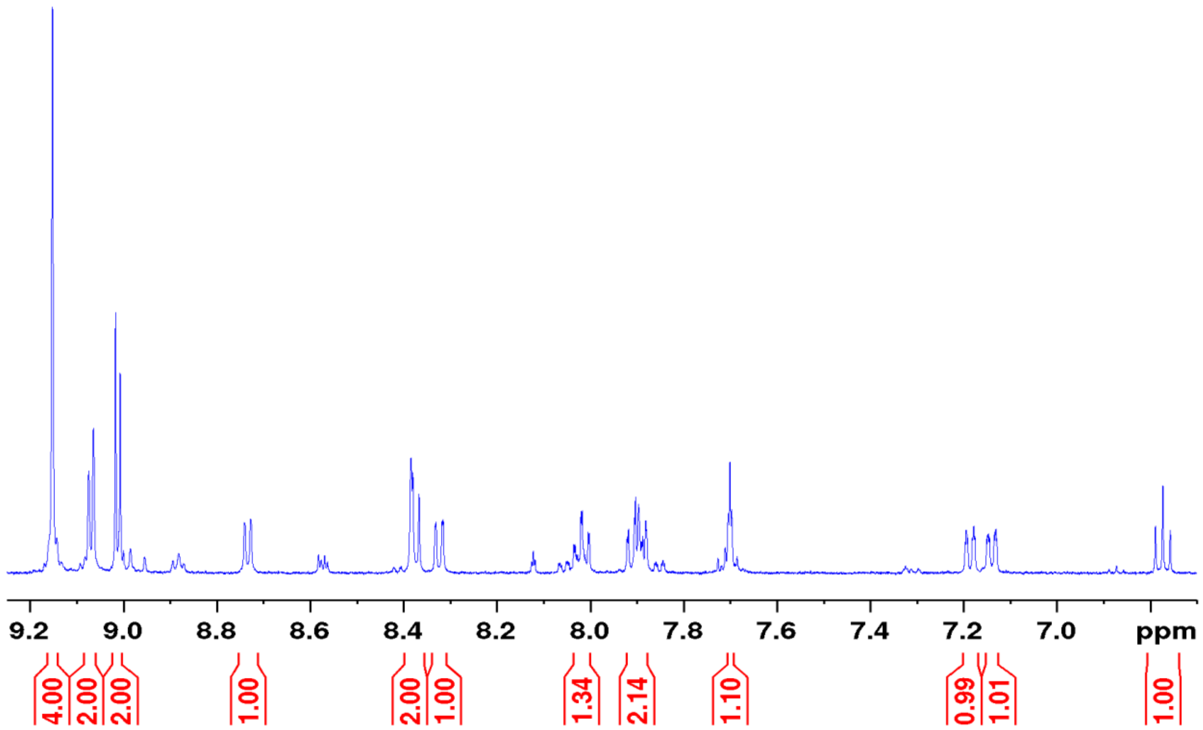

Figure S9.2: Aromatic section of the ^{1}H NMR spectrum of **1f** (500 MHz, acetone-d_6, TFA-d_1, 298 K).

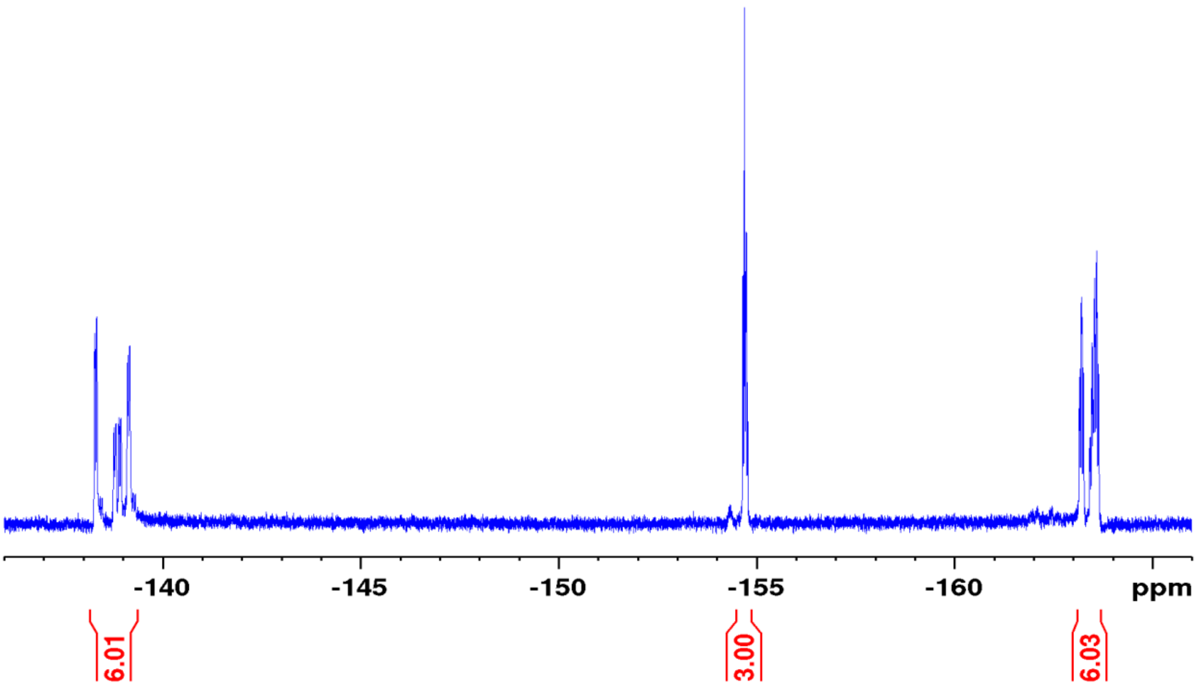

Figure S9.3: ^{19}F NMR spectrum of **1f** (471 MHz, acetone-d_6, 298 K).

Synthesis of bis(5-(biphenylazo-mercaptopyridine)-10,15,20-tris(pentafluorophenyl)nickel(II)porphyrin) disulfide (1g)

Trifluoroacetic acid (10 mL) and anisole (0.2 mL) were cooled to 0 °C and porphyrin **1f** (30 mg, 24.8 µmol) and mercury acetate (7.90 mg, 24.8 µmol) were added. The mixture was stirred at 0 °C for 1.5 h. An aqueous solution of hydrogen sulfide (10 mL, 1 M) was added and stirred for 10 min at ambient temperature. The aqueous layer was extracted with dichloromethane (5 × 25 mL). The combined organic layers were dried over magnesium sulfate and the solvent was removed in vacuo. The crude product was applied on silica and purified by column chromatography (silica, diethyl ether/toluene+trifluoroacetic acid 1:1+0.1%, R_f (1:1+0.1%) = 0.97). The product was obtained as symmetric disulfide, a red solid.

Yield: 21.5 mg (9.33 µmol, 75%)

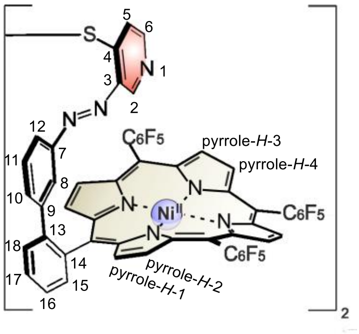

M.p.: 249 °C

^{1}H NMR (600 MHz, acetone-d_6, TFA-d_1, 298 K): δ = 9.27-8.79 (m, 9H, pyrrole-*H*-1/2/3/4, *H*-2), 8.31-8.09 (m, br, 1H, *H*-6), 8.18 (d, ^{3}J = 7.50 Hz, 1H, *H*-15), 7.90 (td, ^{3}J = 7.75 Hz ^{4}J = 0.70 Hz, 1H, *H*-17), 7.80 (td, ^{3}J = 7.50 Hz, ^{4}J = 0.70 Hz, 1H, *H*-16), 7.76 (d, ^{3}J = 7.75 Hz, 1H, *H*-18), 7.30 (s, br, 1H, *H*-5), 6.99 (d, ^{3}J = 7.90 Hz, 1H, *H*-12), 6.95 (t, ^{4}J = 1.71 Hz,, 1H, *H*-8), 6.53 (d, ^{3}J = 7.90 Hz, 1H, *H*-10), 6.35 (t, ^{3}J = 7.90 Hz, 1H, *H*-11) ppm.

^{19}F NMR (471 MHz, acetone-d_6, TFA-d_1 298 K): δ = -138.4 - -139.4 (m, 6F, *o*-*F*), -154.2 - -154.7 (m, 3F, *p*-*F*), -163.0 - -163.9 (m, 6F, *m*-*F*) ppm.

HR-ESI-MS: m/z = 1152.05311 (calcd. for $C_{55}H_{19}F_{15}N_7NiS$), 1152.05269 (found) (-0.37 ppm).

MALDI-MS (Cl-CCA): m/z = 1152.1 [M- $C_{55}H_{19}F_{15}N_7NiS]^{+}$.

FT-IR (ATR): ν = 2923 (w), 2855 (w), 1725 (w), 1651 (w), 1589 (w), 1519 (s), 1488 (s), 1375 (w), 1340 (m), 1162 (w), 1071 (m), 1054 (m), 986 (s), 956 (m), 938 (s), 927 (s), 871 (w), 838 (w), 800 (m), 779 (m), 707 (m), 693 (m), 672 (w), 658 (w) cm^{-1}.

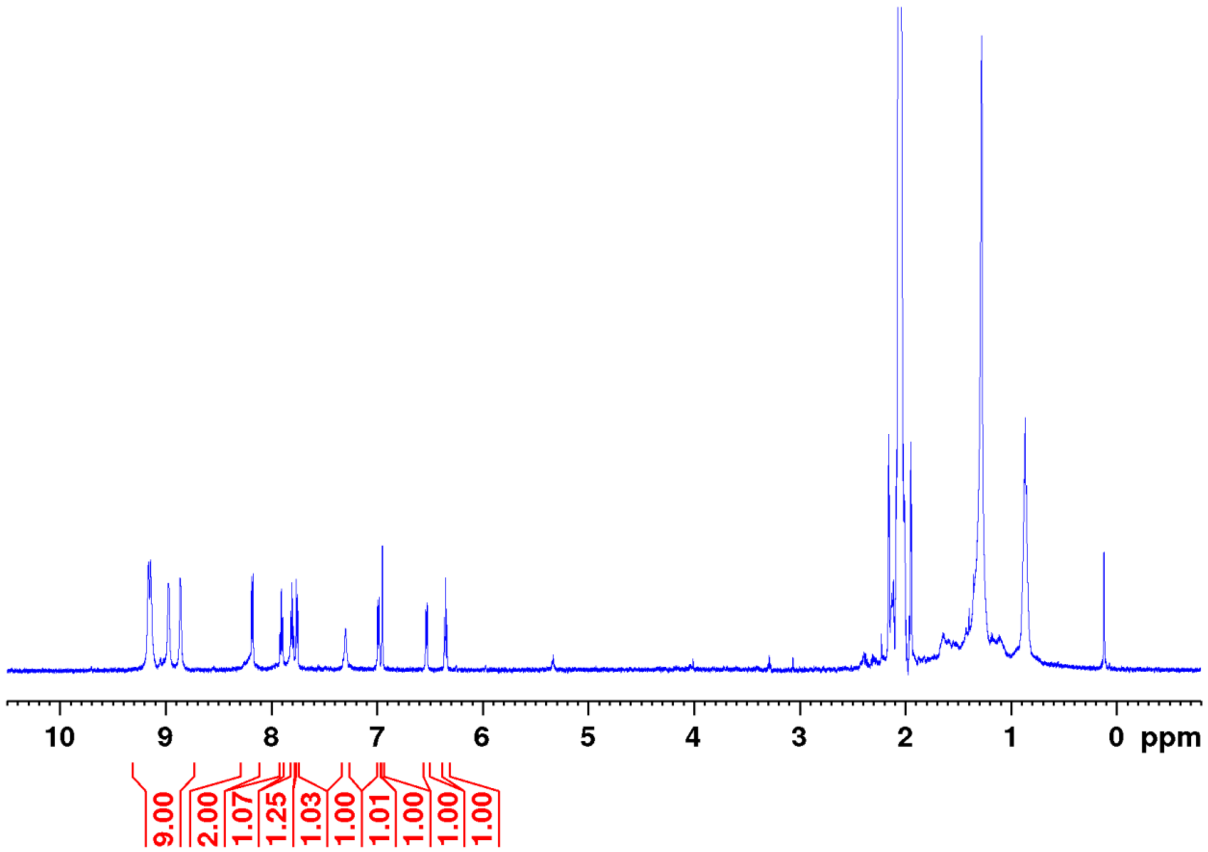

Figure S10.1: Full ^{1}H NMR spectrum of **1g** (600 MHz, acetone-d_6, TFA-d_1, 298 K).

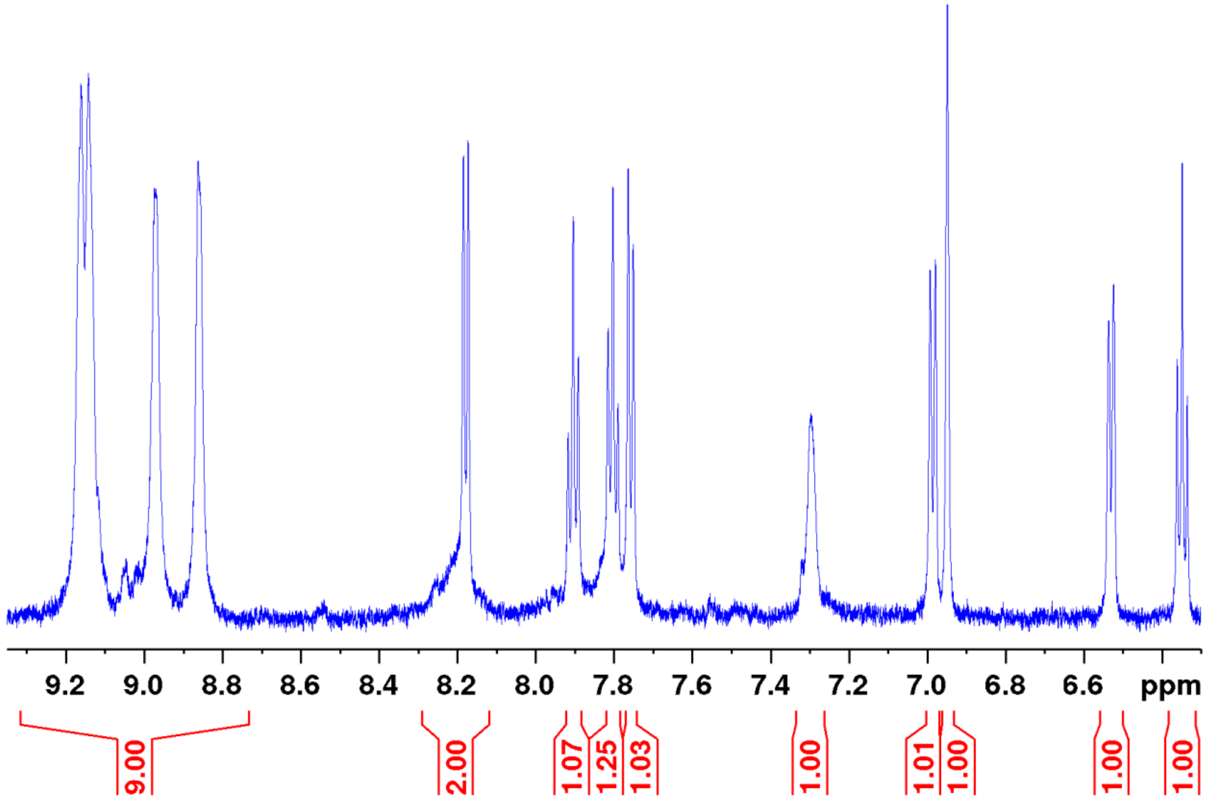

Figure S10.2: Aromatic section of the ^{1}H NMR spectrum of **1g** (600 MHz, acetone-d_6, TFA-d_1, 298 K).

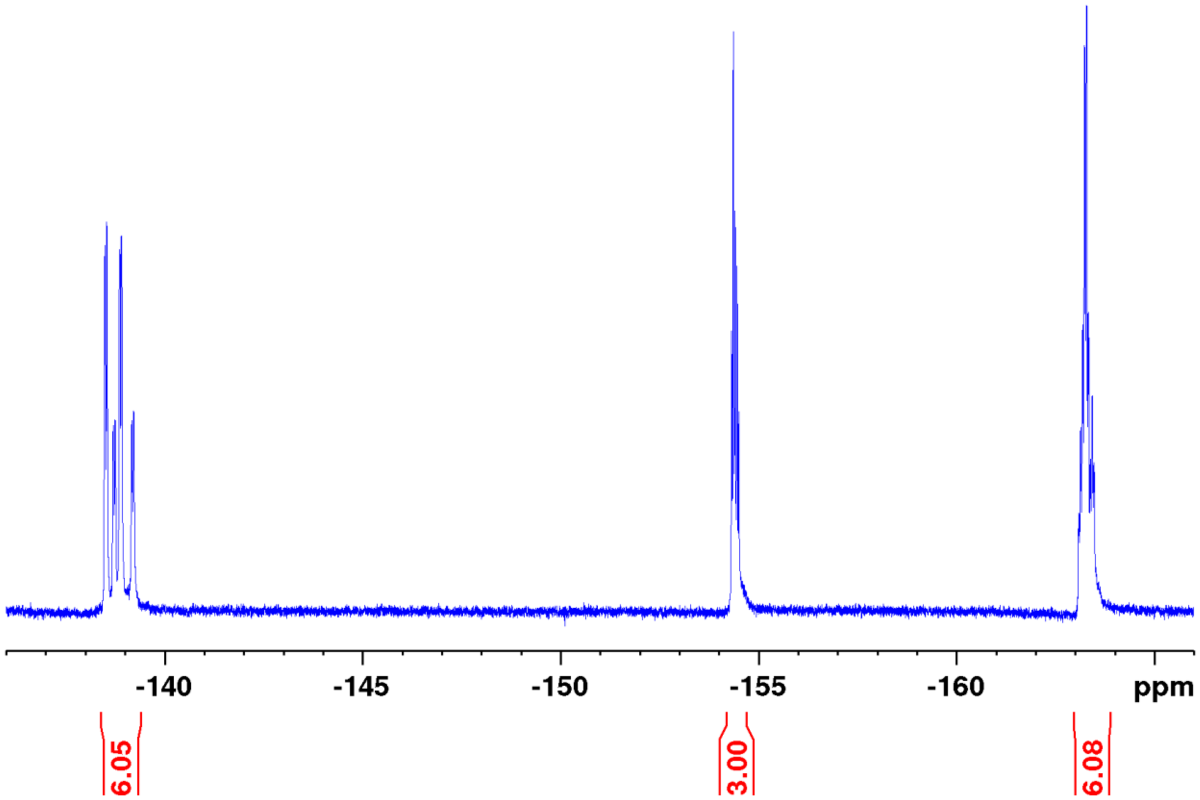

Figure S10.3: ^{19}F NMR spectrum of **1g** (471 MHz, acetone-d_6, TFA-d_1 298 K).

Synthesis of 5-(biphenylazo-mercaptopyridine)-10,15,20-tris(pentafluorophenyl)nickel(II)porphyrin (1h)

Under an atmosphere of nitrogen 5-(biphenylazo-mercaptopyridine)-10,15,20-tris(pentafluorophenyl)nickel(II)porphyrin disulfide (1.10 mg, 0.477 µmol, **1g**) was dissolved in dry tetrahydrofuran (1 mL). Catecholborane (19 µL, 50 mM in tetrahydrofurane) was added and the mixture was stored at ambient temperature for 20 h. Methanol (200 µL) and an excess of ammonium chloride were added and the mixture was vigorously shaken and allowed to stand for 1 h. There was no remaining disulfide detected by NMR spectroscopy and HRESIMS. Solid material was filtered off and the solution was directly used in NMR- and UV–vis measurements. Towards

this end the required volume of the stock solution was added into the NMR tube or cuvette under an atmosphere of nitrogen. The solvent was removed in vacuo to yield a red solid and the vessel was refilled with the desired solvent for the measurements under inert atmosphere. Purification by chromatography was not possible due to the extreme oxygen sensitivity of the thiol. NMR spectra therefore contain quenching products of catecholborane.

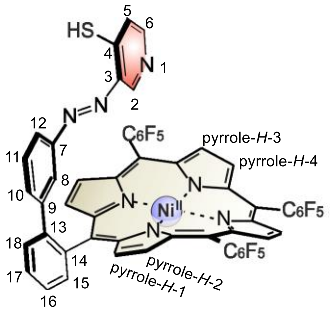

^{1}H NMR (500 MHz, acetone-d_6, TFA-d_1, 298 K): δ = 9.07-9.02 (m, 4H, pyrrole-*H*-3/4), 9.00-8.95 (m, pyrrole-*H*-2/1), 8.70 (dd, ^3J = 6.65 Hz, ^4J = 0.98 Hz, 1H, *H*-6), 8.55 (d, ^4J = 0.98 Hz, 1H, *H*-2), 8.26 (dd, ^3J = 8.11 Hz, ^4J = 1.52 Hz, 1H, *H*-15), 8.00 (td, ^3J = 7.82 Hz, ^4J = 1.50 Hz, 1H, *H*-17), 7.94 (d, ^3J = 6.65 Hz, 1H, *H*-5), 7.92-7.85 (m, 2H, *H*-16/18), 7.70 (t, ^4J = 1.82 Hz, 1H, *H*-8), 7.30-7.27 (m, 1H, *H*-10), 7.22-7.19 (m, 1H, *H*-12), 6.85 (t, ^3J = 7.88 Hz, 1H, *H*-11) ppm.

^{19}F NMR (471 MHz, acetone-d_6, 298 K): δ = -138.3 - -139.7 (m, 6F, *o*-*F*), -154.4 - -154.7 (m, 3F, *p*-*F*), -163.1 - -163.7 (m, 6F, *m*-*F*) ppm.

HR-ESI-MS: m/z = 1154.06876 (calcd. for $C_{55}H_{21}F_{15}N_7NiS$), 1154.0675 (found) (-1.09 ppm).

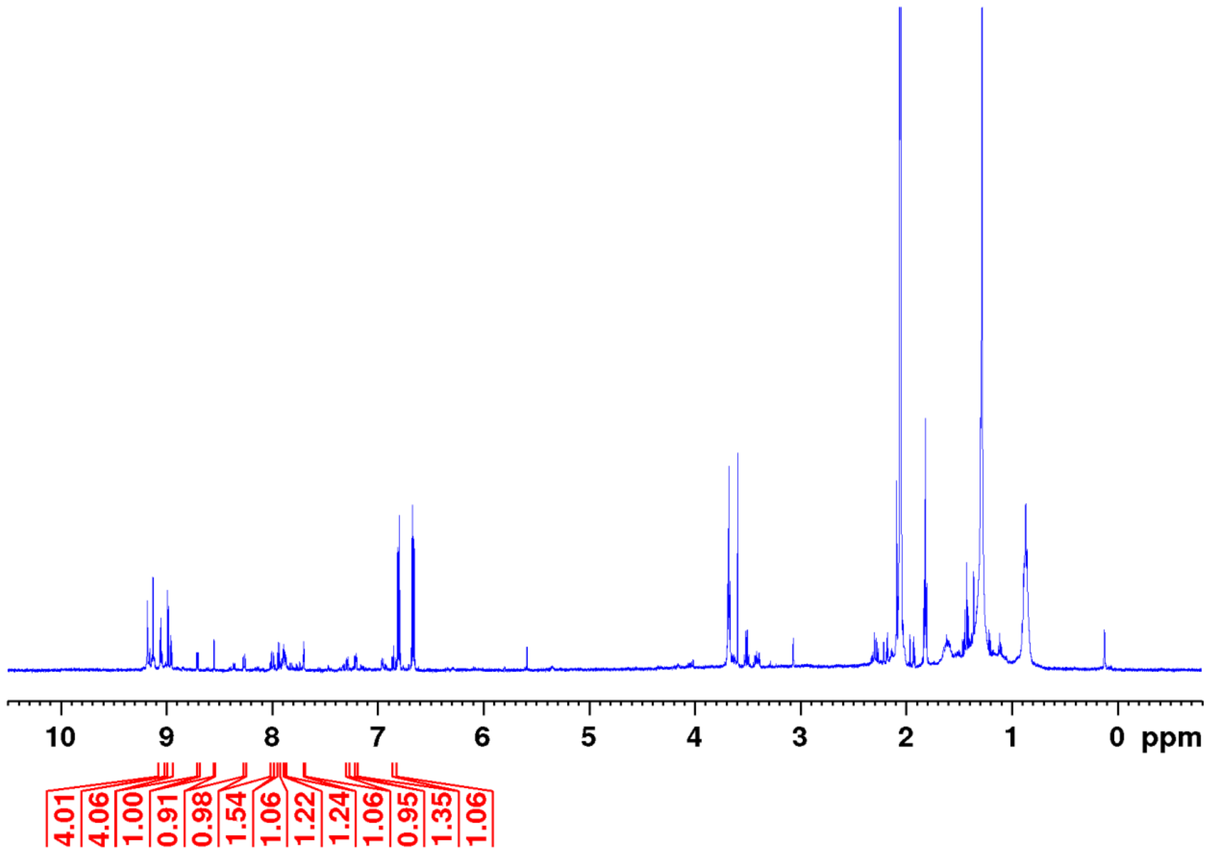

Figure S11.1: Full ^{1}H NMR spectrum of **1h** (600 MHz, acetone-d_6, TFA-d_1, 298 K).

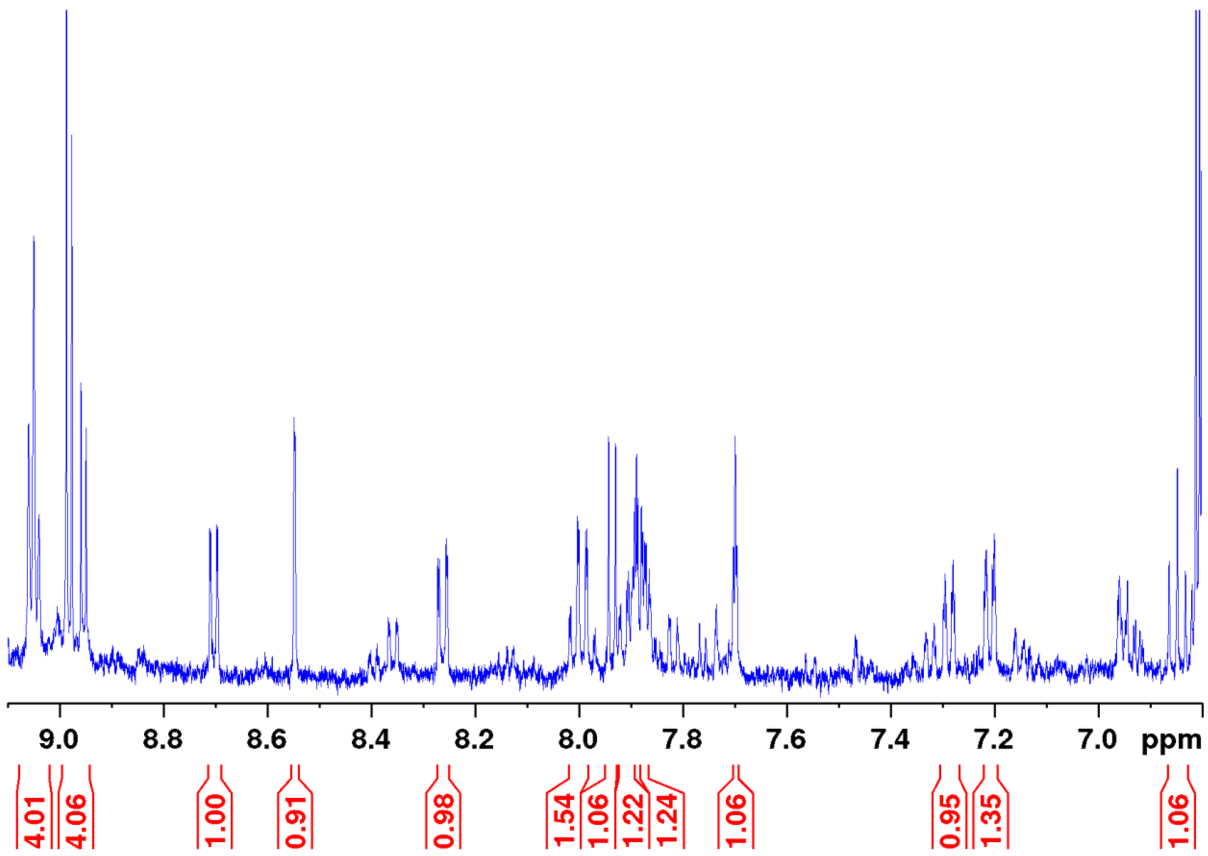

Figure S11.2: Aromatic section of the ^{1}H NMR spectrum of **1h** (600 MHz, acetone-d_6, TFA-d_1, 298 K).

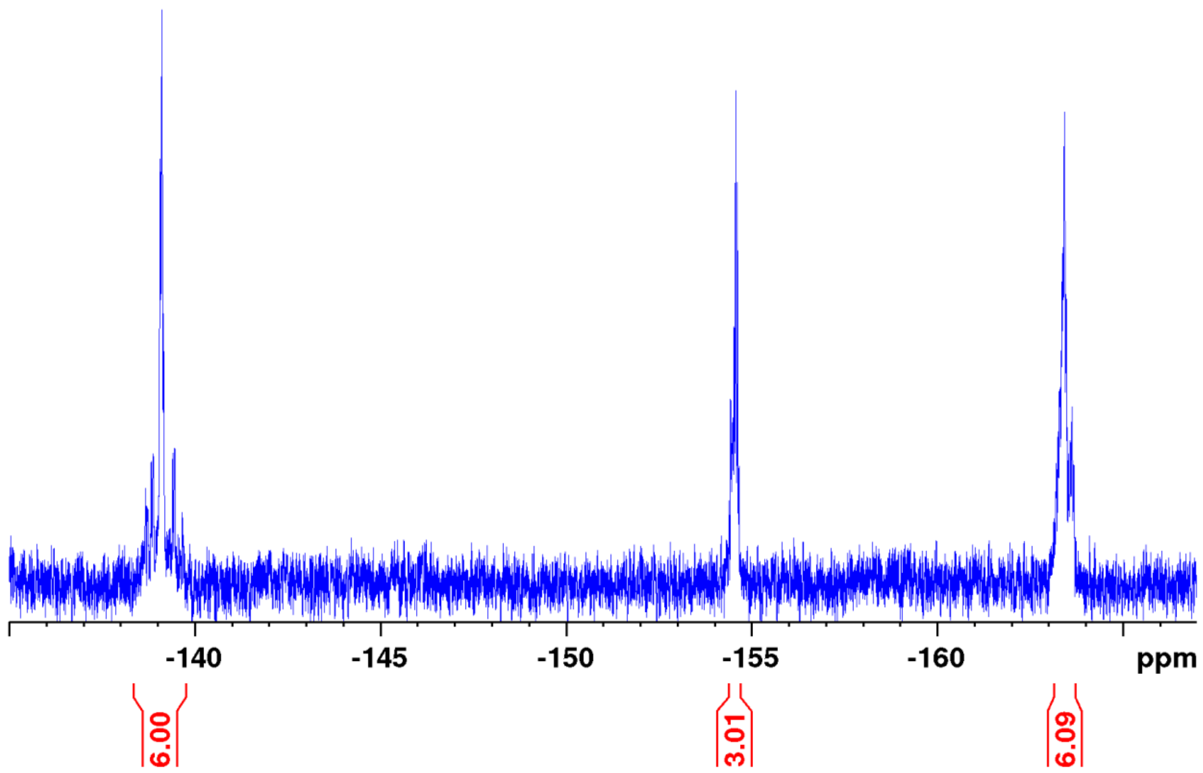

Figure S11.3: ^{19}F NMR spectrum of **1h** (471 MHz, acetone-d_6, TFA-d_1 298 K).

Synthesis of 5-(biphenylazo-cyanopyridine)-10,15,20-tris(pentafluorophenyl)nickel(II)porphyrin (1i)

The solvent (25 mL 1,4-dioxane, 3 mL deionized water) was flushed with nitrogen for 70 min. Porphyrin precursor (100 mg, 93.7 µmol, **22**), 3-(3-bromophenylazo)-4-cyanopyridine (36.8 mg, 128 µmol, **20**), cesium carbonate (56.0 mg, 172 µmol), potassium fluoride (10.0 mg, 172 µmol) and [1,1´-bis(diphenylphosphino)ferrocene]dichloropalladium(II) (10 mg, 13.7 µmol) were added and stirred for 18 h at 60 °C. The mixture was diluted with a saturated aqueous solution of sodium chloride and dichloromethane (each 25 mL). Layers were

separated and the aqueous layer was extracted with dichloromethane (2 × 25 mL). The combined organic layers were dried over magnesium sulfate and the solvent was removed in vacuo. The crude product was applied on silica and purified by column chromatography (silica, cyclohexane/ethyl acetate + trifluoroacetic acid 3:1+0.1%, R_f (3:1+0.1%) = 0.42). The product was obtained as a red solid.

Yield: 101 mg (88.0 µmol, 94%)

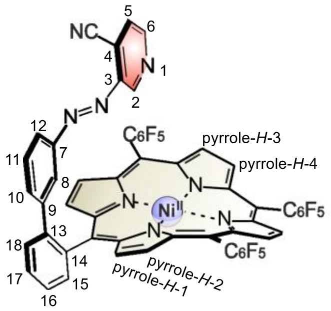

M.p.: 148 °C

^{1}H NMR (500 MHz, acetone-d_6, TFA-d_1, 298 K): δ = 9.15 (s, 4H, pyrrole-*H*-3/4), 9.09 (d, ^3J = 5.02 Hz, 2H, pyrrole-*H*-2), 9.04-9.01 (m, ^3J = 5.02 Hz, 3H, pyrrole-*H*-1,*H*-2), 8.74 (s, br, 1H, *H*-6), 8.31 (dd, ^3J = 7.70 Hz, ^4J = 1.20 Hz, 1H, *H*-15), 8.14 (d, ^3J = 5.00 Hz, 1H, *H*-5), 7.98 (td, ^3J = 7.72 Hz, ^4J = 1.32 Hz, 1H, *H*-17), 7.90-7.85 (m, 2H, *H*-16/18), 7.72 (t, ^4J = 1.82 Hz, 1H, *H*-8), 7.20 (ddd, ^3J = 7.86 Hz, ^4J = 1.83 Hz, ^4J = 1.03 Hz, 1H, *H*-10), 7.17 (ddd, ^3J = 7.86 Hz, ^4J = 1.80 Hz, ^4J = 1.03 Hz, 1H, *H*-12), 6.76 (t, ^3J = 7.85 Hz, 1H, *H*-11) ppm.

^{19}F NMR (471 MHz, acetone-d_6, 298 K): δ = -138.3 - -139.3 (m, 6F, *o*-*F*), -154.6 - -154.9 (m, 3F, *p*-*F*), -163.1 - -163.7 (m, 6F, *m*-*F*) ppm.

EI-MS (70 eV): m/z (%) = 1146.10 (100) $[M]^+$, 1015.06 (8) $[M-C_6H_3N_4]^+$, 103.03 (12) $[M-C_{50}H_{16}F_{15}N_6Ni]^+$.

HR-EI-MS (70 eV): m/z = 1146.08466 (calcd. for $C_{56}H_{19}F_{15}N_8Ni$), 1146.08753 (found) (2.50 ppm).

HR-ESI-MS: m/z = 1147.09194 (calcd. for $C_{56}H_{20}F_{15}N_8Ni$), 1147.09090 (found) (-0.90 ppm).

MALDI-MS (Cl-CCA): m/z = 1147.2 $[M+H]^+$.

FT-IR (ATR): ν = 2923 (m), 2855 (w), 1518 (s), 1487 (s), 1431 (m), 1400 (w), 1348 (m), 1261 (w), 1203 (w), 1166 (w), 1141 (w), 1072 (m), 1059 (m), 1005 (m), 986 (s), 958 (m), 939 (s), 926 (s), 864 (w), 839 (m), 802 (m), 763 (s), 744 (m), 726 (m), 703 (m), 693 (m), 659 (w), 641 (w), 620 (w) cm^{-1}.

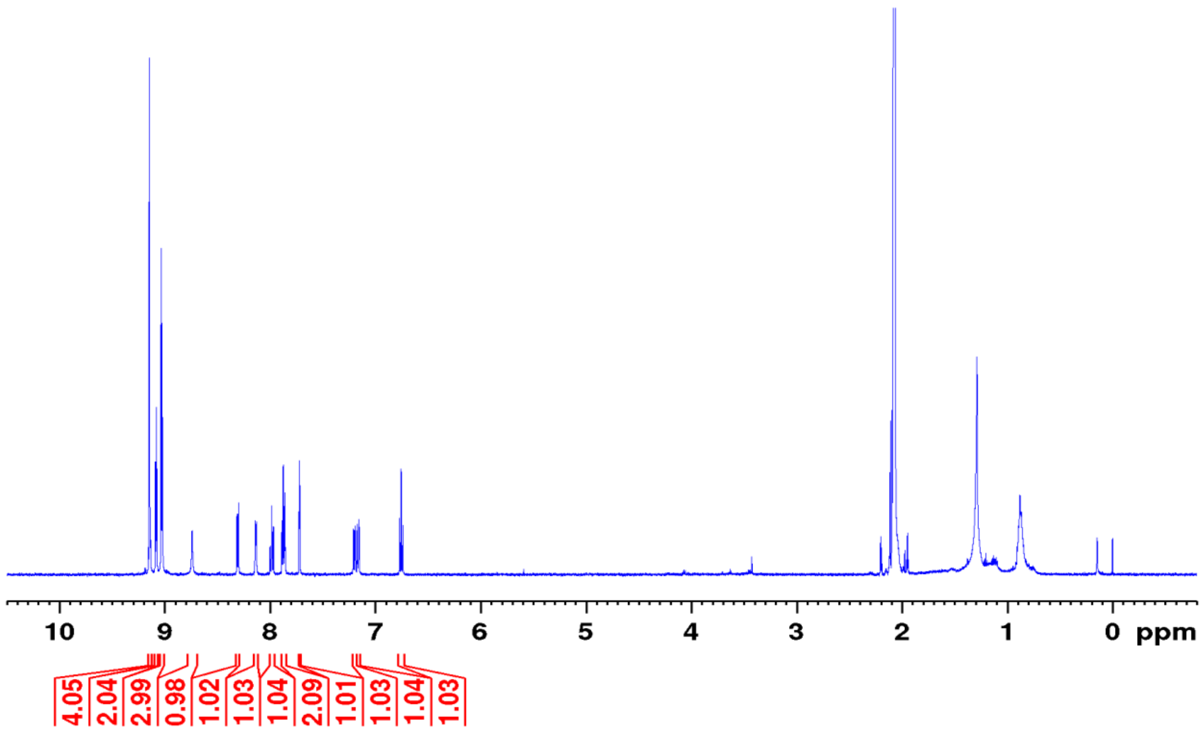

Figure S12.1: Full ^1H NMR spectrum of **1i** (500 MHz, acetone-d_6, TFA-d_1, 298 K).

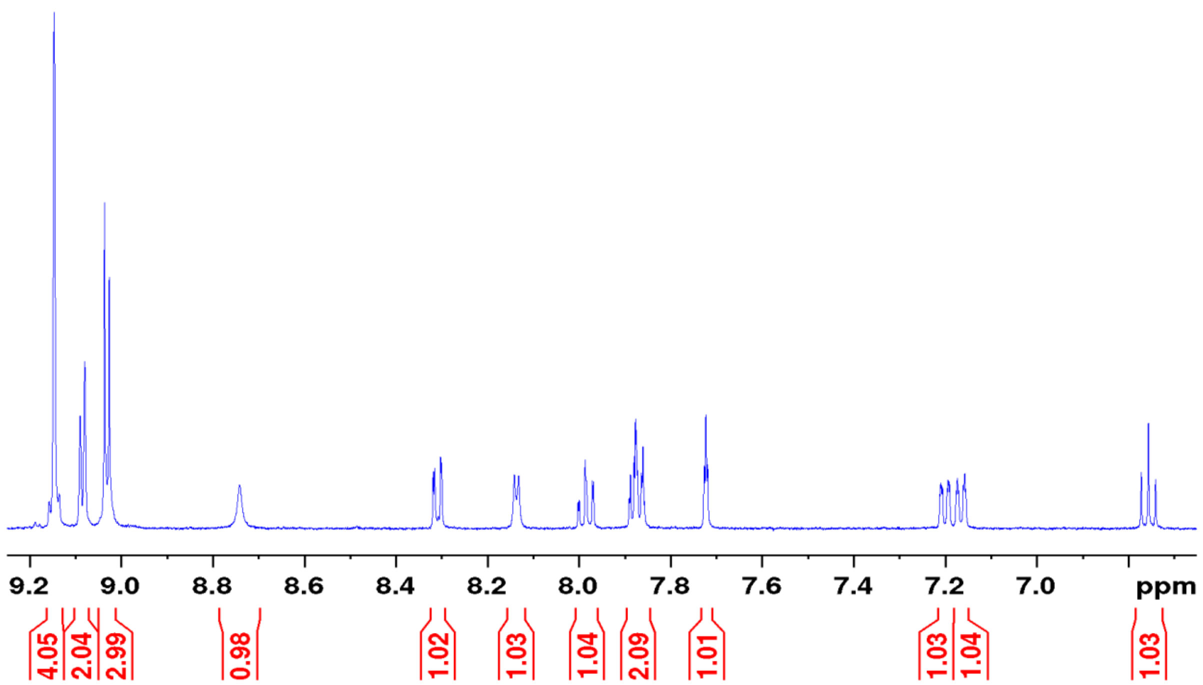

Figure S12.2: Aromatic section of the ^{1}H NMR spectrum of **1i** (500 MHz, acetone-d_6, TFA-d_1, 298 K).

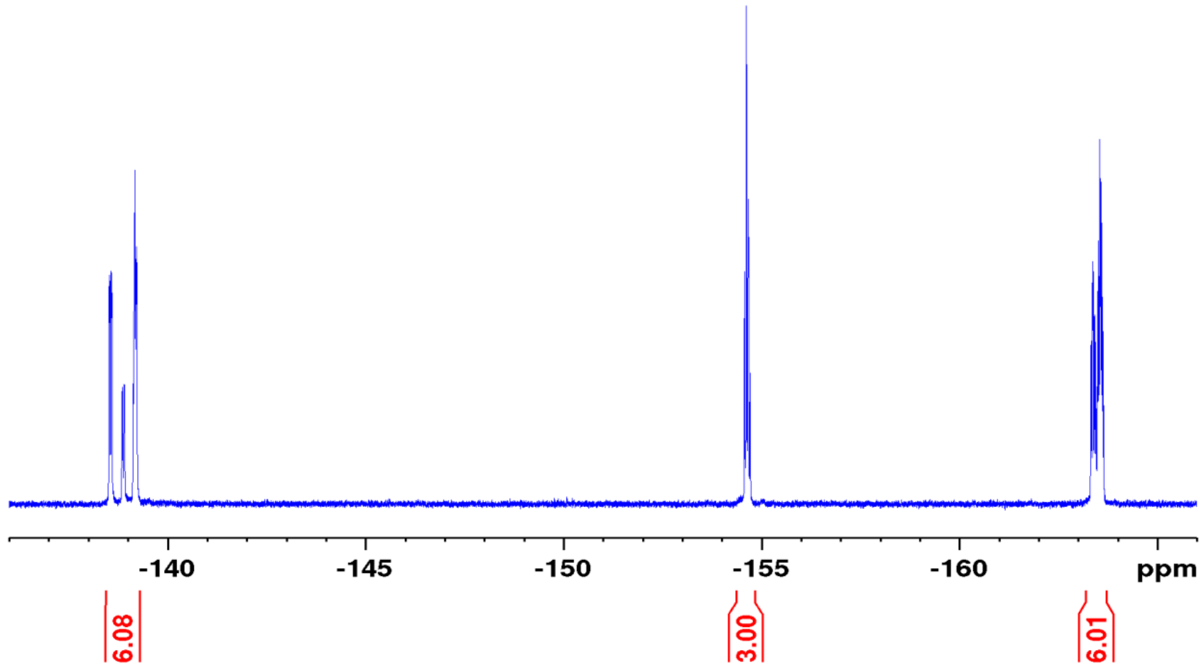

Figure S12.3: ^{19}F NMR spectrum of **1i** (471 MHz, acetone-d_6, 298 K).

Synthesis of 5-(biphenylazo-isonicotinic acid)-10,15,20-tris(pentafluorophenyl)nickel(II)porphyrin (1j)

The solvent (5 mL 1,4-dioxane, 0.6 mL deionized water) was flushed with nitrogen for 30 min. Porphyrin precursor (52.3 mg, 49.0 µmol, **22**), 3-(3-bromophenylazo)-4-pyridinecarboxylic acid (10.0 mg, 32.7 µmol, **21**), cesium carbonate (16.0 mg, 81.8 µmol) and [1,1´-bis(diphenylphosphino)ferrocene]dichloropalladium(II) (10 mg, 13.7 µmol) were added and stirred for 43 h at 90 °C. The mixture was diluted with glacial acetic acid (5 mL) and dichloromethane (25 mL). Layers were separated and the organic layer was washed with deionized water (2 × 50 mL). The solvent was removed in vacuo the crude product was applied on Celite® and purified by column chromatography (silica, dichloromethane/methanol/glacial acetic acid 95:5:0 → 89:10:1, R_f (5:1:0) = 0.68). The product was obtained as a red solid.

Yield: 20.0 mg (17.2 µmol, 52%)

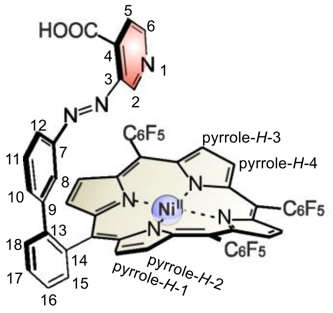

M.p.: 210 °C

¹H NMR (500 MHz, acetone-d_6, TFA-d_1, 300 K): δ = 9.18 (d, ^3J = 5.83 Hz, 1H, *H*-6), 9.10 (s, 4H, pyrrole-*H*-3/4), 9.02 (d, ^3J = 4.96 Hz, 2H, pyrrole-*H*-2), 8.97 (d, ^3J = 4.96 Hz, 2H, pyrrole-*H*-1), 8.83 (s, 1H, *H*-2), 8.36 (d, ^3J = 5.83 Hz, 1H, *H*-5), 8.28-8.25 (m, 1H, *H*-15), 8.00 (td, ^3J = 7.74 Hz, ^4J = 1.32 Hz, 1H, *H*-17), 7.89-7.85 (m, 2H, *H*-16/18), 7.77 (t, ^4J = 1.81 Hz, 1H, *H*-8), 7.17-7.14 (m, 2H, *H*-10/12), 6.74 (t, ^3J = 7.89 Hz, 1H, *H*-11) ppm.

^{19}F NMR (471 MHz, acetone-d_6, 300 K): δ = -139.7 - -140.4 (m, 6F, *o-F*), -155.6 - -155.8 (m, 3F, *p-F*), -164.3 - -164.7 (m, 6F, *m-F*) ppm.

HR-ESI-MS: m/z = 1166.08652 (calcd. for $[C_{56}H_{21}F_{15}N_7NiO_2]^+$), 1166.08600 (found) (-0.45 ppm).

MALDI-MS (Cl-CCA): m/z = 1166.0 $[M+H]^+$.

FT-IR (ATR): ν = 2922 (m), 2852 (m), 1730 (m), 1597 (m), 1519 (s), 1490 (s), 1377 (m), 1342 (m), 1259 (m), 1071 (s), 1054 (s), 986 (s), 956 (m), 938 (s), 927 (s), 838 (m), 801 (s), 763 (s), 741 (m), 705 (m) cm^{-1}.

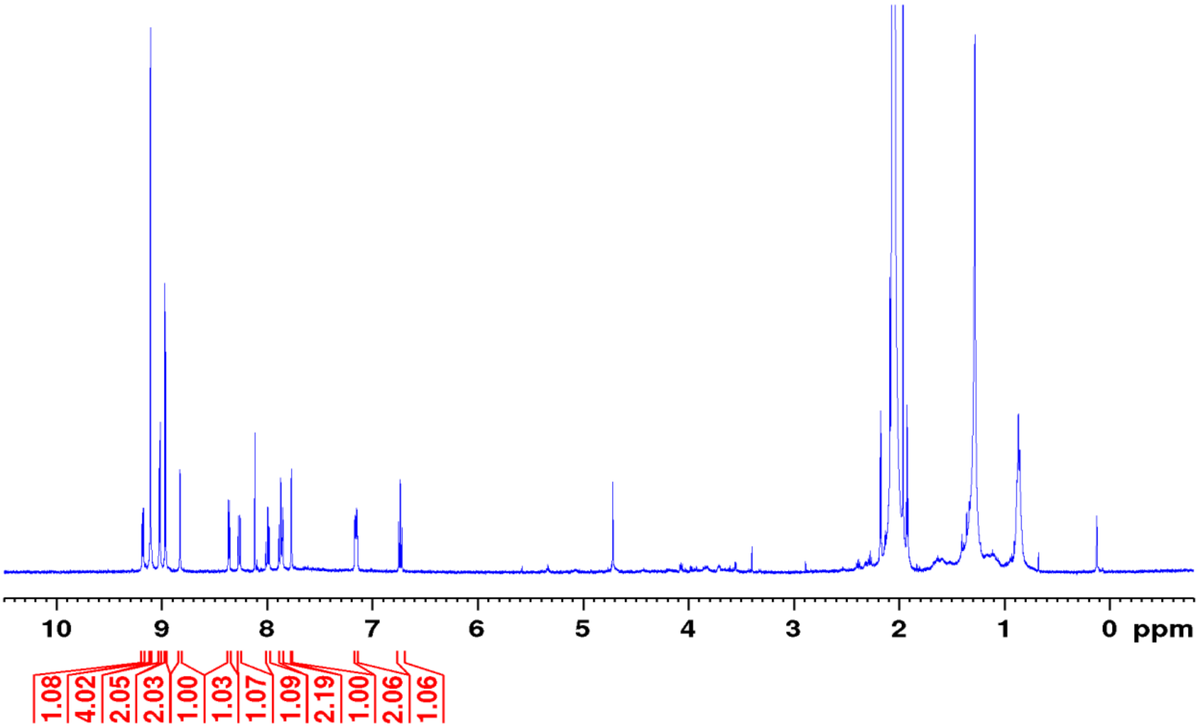

Figure S13.1: Full ^{1}H NMR spectrum of **1j** (500 MHz, acetone-d_6, TFA-d_1, 300 K).

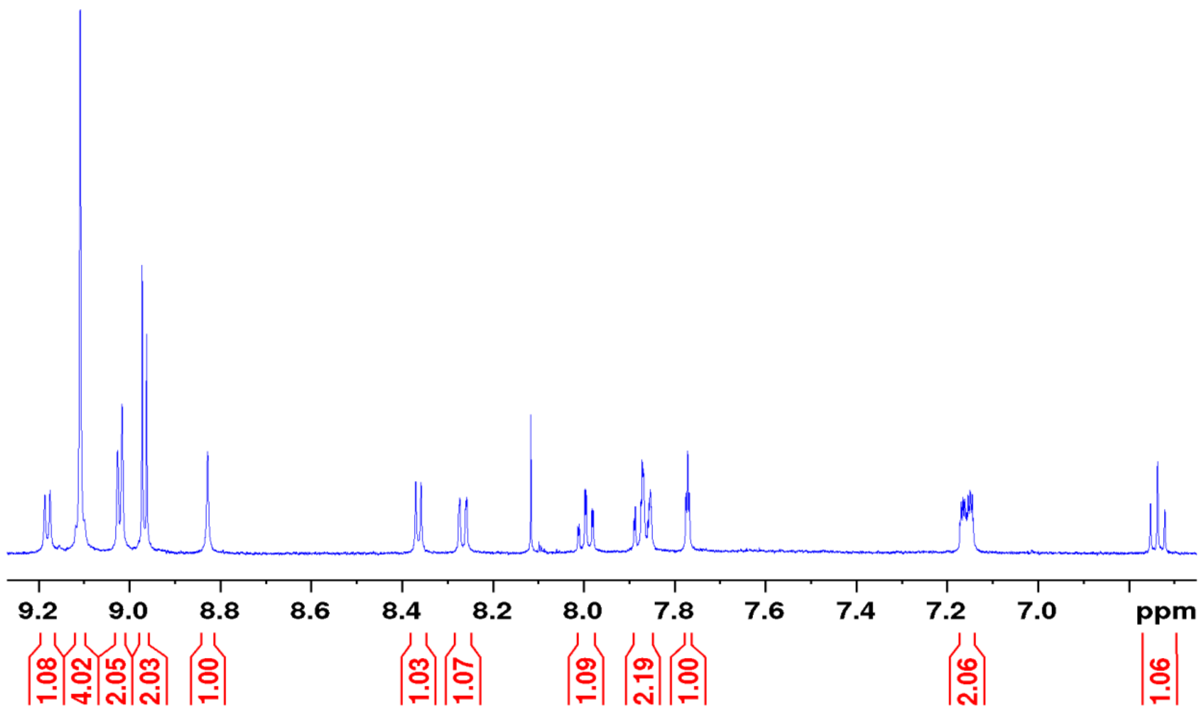

Figure S13.2: Aromatic section of the ^{1}H NMR spectrum of **1j** (500 MHz, acetone-d_6, TFA-d_1, 300 K).

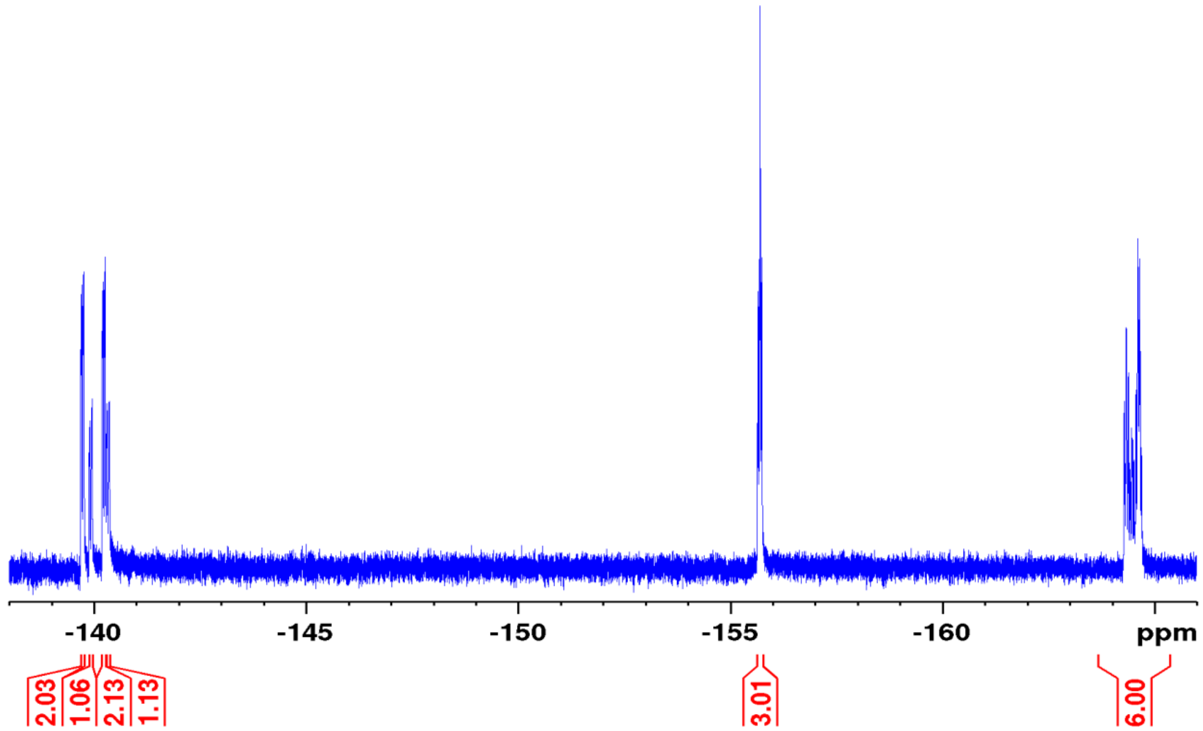

Figure S13.3: ^{19}F NMR spectrum of **1j** (471 MHz, acetone-d_6, 300 K).

III: Procedure for photophysical experiments

The ratio of [coordinated (*cis*-para)] / [decoordinated (*cis*-dia)] isomer was determined from the average shift of the porphyrin pyrrole protons in ^{1}H NMR experiments [5]. The *cis-trans* ratio was obtained by integration of *H*-11 in ^{1}H NMR and if necessary by UV/vis spectroscopy. For the deprotonation of **1e**, **1h**, **1j** cesium carbonate was used. The reduction of the disulfide **1g** was performed in THF using 2 equivalents of catecholborane (solution in THF) under Schlenk conditions.

Determination of the coordinated *cis* isomer by ^{1}H NMR spectroscopy

Solutions of the record player molecules **1e–j** in acetone-d_6 were irradiated with light of 505 nm and ^{1}H NMR spectra were recorded. The average shift of the paramagnetic pyrrole protons at 298 °C was determined and compared to the maximum shift of a Ni(II) porphyrin with a coordination number of five (CN=5) in acetone-d_6 at 298 K, developed by Gutzeit et al. [6]. The amount of paramagnetic *cis*-isomer was calculated by equation [6]:

$$\%_{cis\text{-}\mathbf{coordinated}} = \frac{\boldsymbol{\delta_{obs.}} - \boldsymbol{\delta_{dia.}}}{\boldsymbol{\delta_{max.}} - \boldsymbol{\delta_{dia.}}} \qquad \text{(Equation S1).}$$

$\%_{cis\text{-coordinated}}$	percentage of paramagnetic *cis* record player.
δ_{obs}.	observed average shift of the *cis* pyrrole protons in ppm.
δ_{dia}.	shift of the completely diamagnetic complex (determined by TFA-d_1 addition) in ppm.
δ_{max}.	maximum shift (100 % coordinated) for a fivefold coordinated complex determined by Gutzeit *et al.* [6] in ppm.

For the molecules already described in literature [3] the values were revised due to results of Gutzeit et al. [6].

Table S1: Percentage of coordinated *cis* **1a–j** determined by equation S1 and applied values for $\delta_{max.}$, $\delta_{obs.}$ and $\delta_{dia.}$.

Molecule	solvent	$\delta_{max.}$ [ppm][a]	$\delta_{obs.}$ [ppm]	$\delta_{dia.}$ [ppm]	$\%_{cis\text{-}coordinated}$
**1a**	acetone-d_6	48.55[b]	41.7[b]	9.08	83
**1b**	acetone-d_6	48.55[b]	47.2[b]	9.08	97
**1c**	acetone-d_6	48.55[b]	48.0[b]	9.08	99
**1d**	acetone-d_6	48.55[b]	39.1[b]	9.08	76
**1e**	acetone-d_6	48.85	46.7	9.08	95
**1e**[c]	acetone-d_6	48.85	48.6	9.08	99
**1f**	acetone-d_6	48.85	46.9	9.05	96
**1g**	acetone-d_6	48.85	48.7	9.08	100
**1h**	acetone-d_6	48.85	48.38	9.02	99
**1h**[c]	acetone-d_6	48.85	47.0	9.02	95
**1i**	acetone-d_6	48.85	24.7	9.08	39
**1j**	acetone-d_6	48.85	48.4	9.05	99
**1j**[c]	acetone-d_6	48.85	48.4	9.05	99

[a] maximum shift (100 % coordinated) for a fivefold coordinated complex determined by Gutzeit et al. [6] in ppm, [b] at 300 K, [c] Deprotonated with Cs_2CO_3,

Determination of the *cis–trans* ratio in ^{1}H NMR

The *cis*-*trans*-ratio for **1f** and **1i** was analysed by integration of *H*-11 because this signal suffers least from paramagnetic line broadening and overlap with other signals [4]. The results are given in Figure S14.

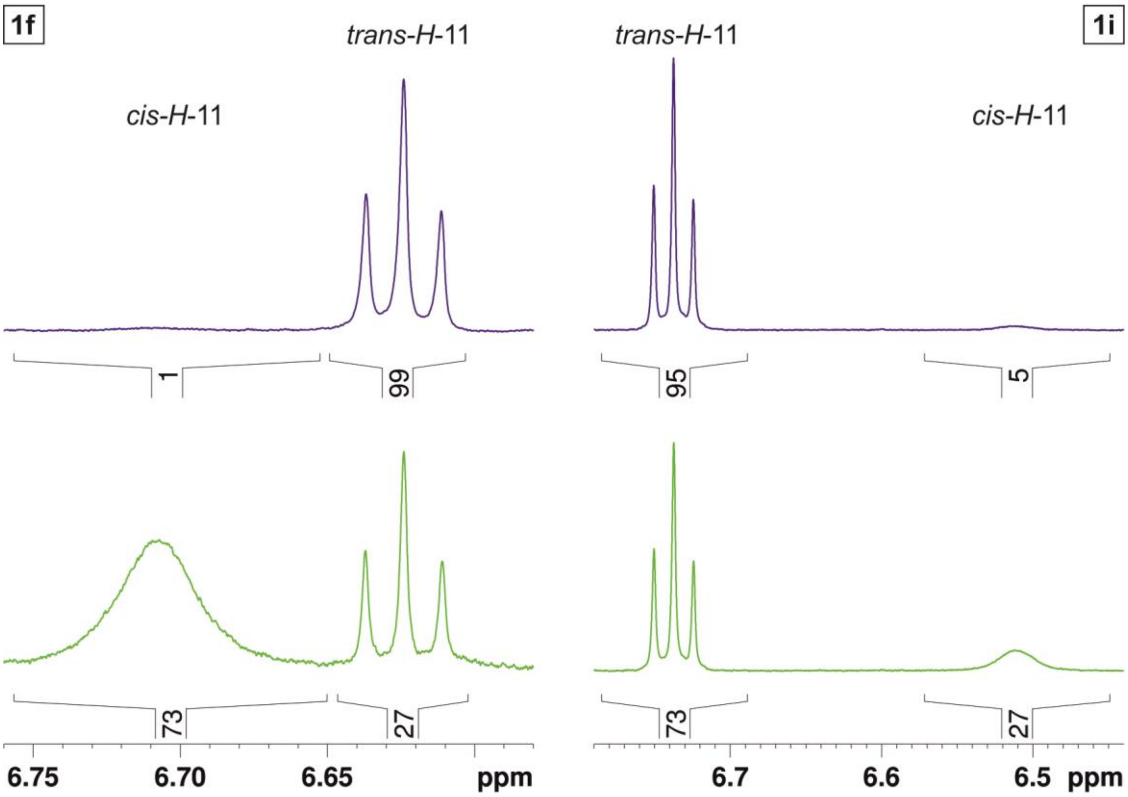

Figure S14: *Cis-trans*-ratio for record player **1f** (left) and **1i** (right) in the PSS at 505 nm (green) and 435 nm (blue) determined by integration of *H*-11 in the ^{1}H NMR (600 MHz, acetone-d_6, 323 K (**1f**)/298 K (**1i**)).

Determination of extinction coefficients of 1e, 1g, 1h, 1j

The extinction coefficient of **1e**, **1g, 1h** and **1j** in acetone in the pure diamagnetic state was determined, by protonation of the pyridine to prevent coordination. Towards this end an excess of trifluoracidic acid was added and the sample was diluted to different concentrations. UV–vis spectra were recorded and the absorbance at λ_{max} of the paramagnetic Soret peak was plotted as a function of the concentration (Figure S15.1-4).

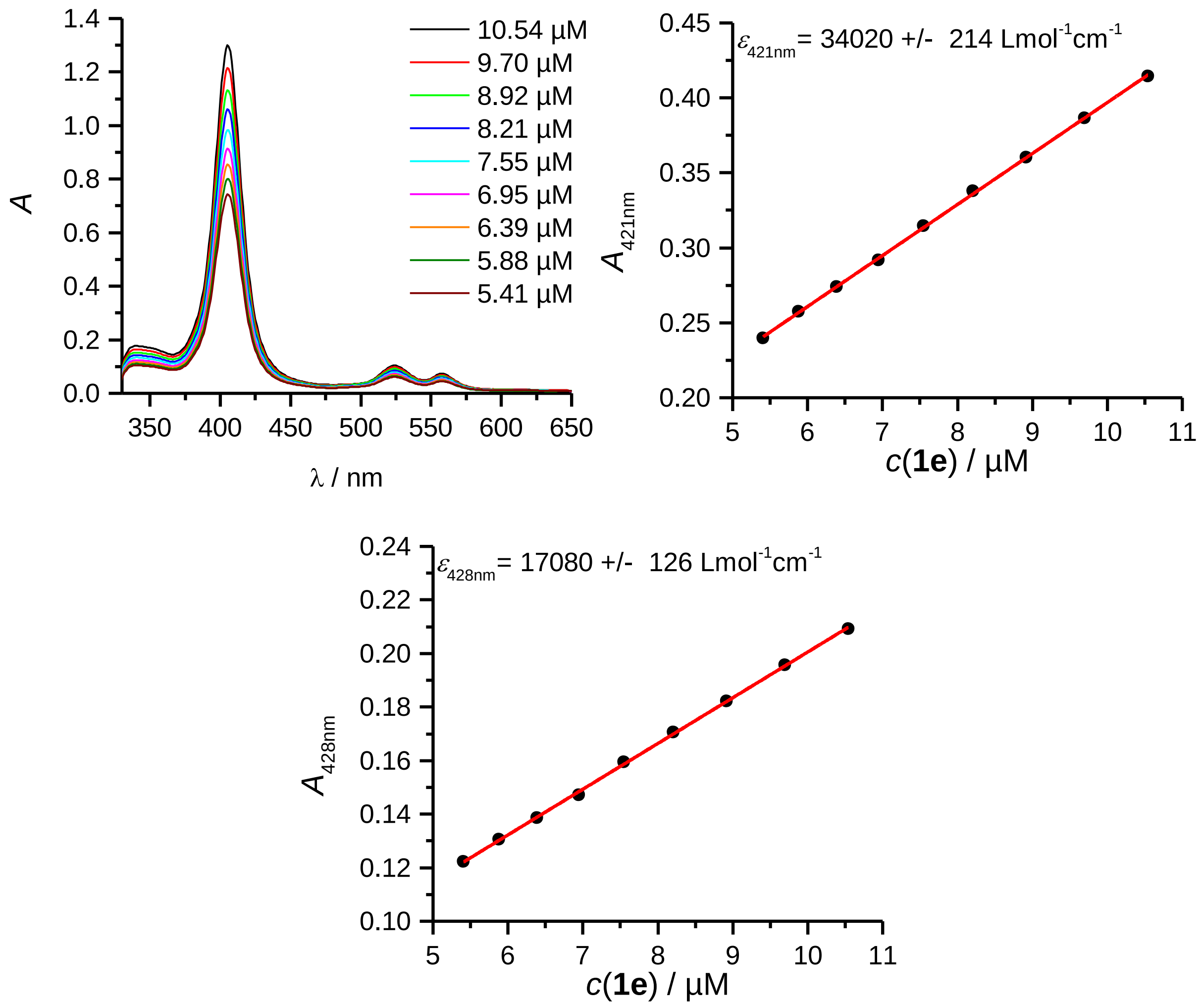

Figure S15.1: UV–vis spectra of completely diamagnetic solutions with different concentrations of porphyrin **1e** in acetone (left) and the plotting of the absorbance at 421 nm / 428 nm as a function of the concentration of **1e**. The extinction coefficient at 421 nm / 428 nm in the completely diamagnetic state was determined as 34020 $Lmol^{-1}cm^{-1}$ / 17080 $Lmol^{-1}cm^{-1}$ (right / bottom).

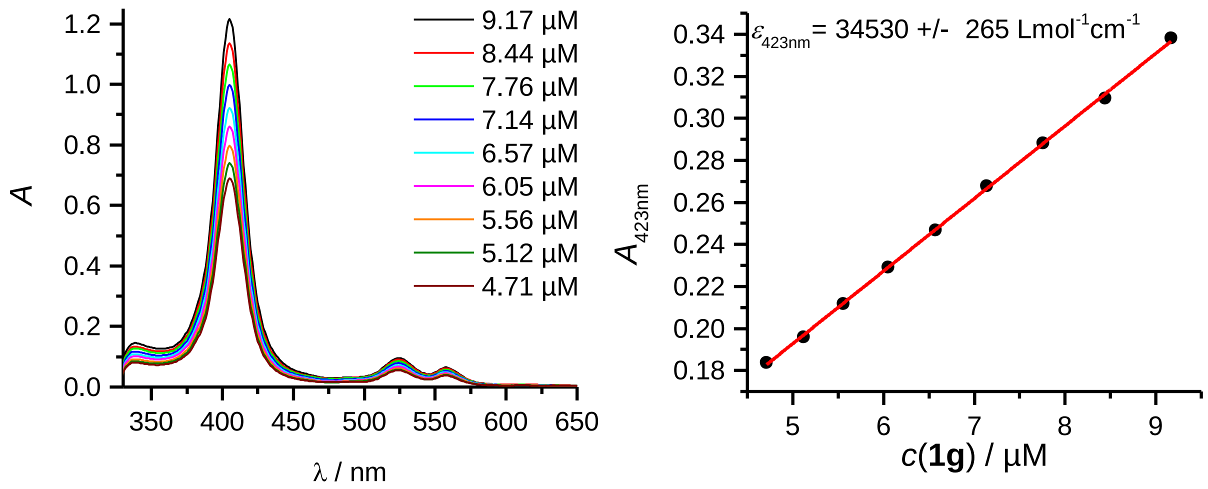

Figure S15.2: UV–vis spectra of completely diamagnetic solutions with different concentrations of porphyrin **1g** in acetone (left) and the plotting of the absorbance at 423 nm as a function of the concentration of **1g**. The extinction coefficient at 423 nm in the completely diamagnetic state was determined as 34530 $Lmol^{-1}cm^{-1}$ (right).

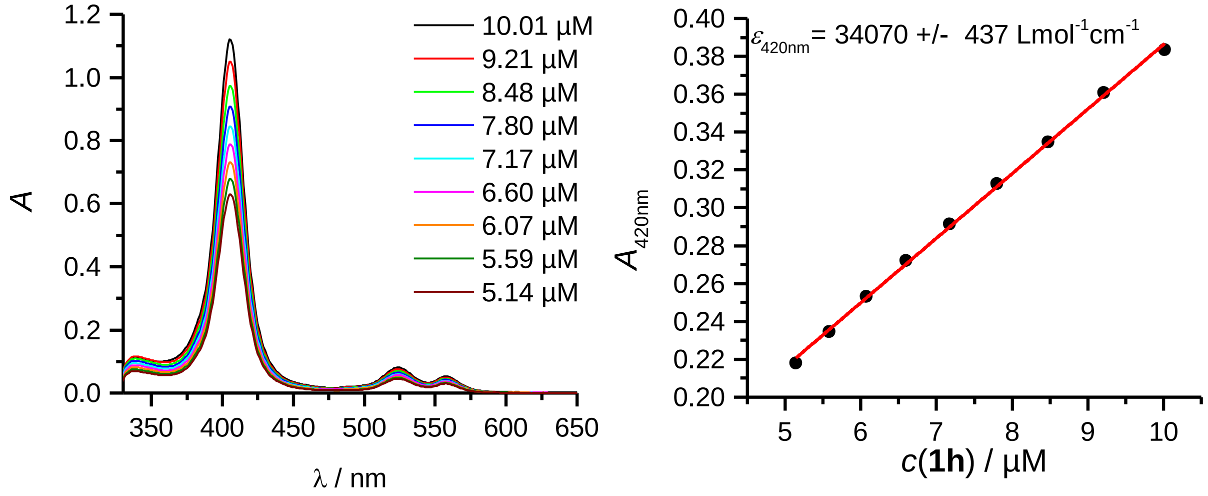

Figure S15.3: UV–vis spectra of completely diamagnetic solutions with different concentrations of porphyrin **1h** in acetone (left) and the plotting of the absorbance at 420 nm as a function of the concentration of **1h**. The extinction coefficient at 420 nm in the completely diamagnetic state was determined as 34070 $Lmol^{-1}cm^{-1}$ (right).

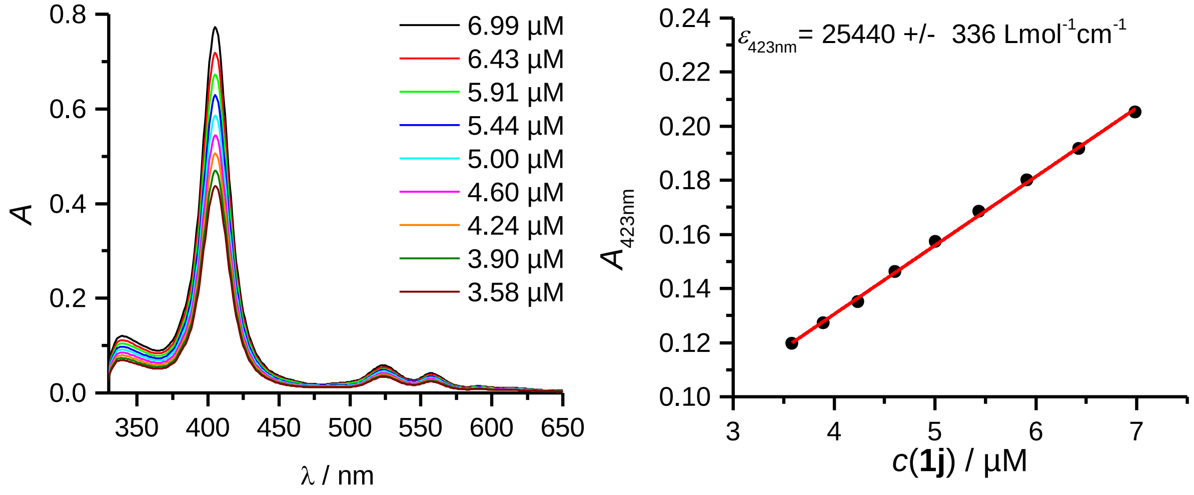

Figure S15.4: UV–vis spectra of completely diamagnetic solutions with different concentrations of porphyrin **1j** in acetone (left) and the plotting of the absorbance at 423 nm as a function of the concentration of **1j**. The extinction coefficient at 423 nm in the completely diamagnetic state was determined as 25440 $Lmol^{-1}cm^{-1}$ (right).

Also the extinction coefficient of the pure paramagnetic compound was determined by adding a huge excess of piperidine as a strong axial ligand. UV–vis spectra were recorded and the absorbance at λ_{max} of the paramagnetic Soret bande was plotted as a function of the concentration (Figure S16.1-4).

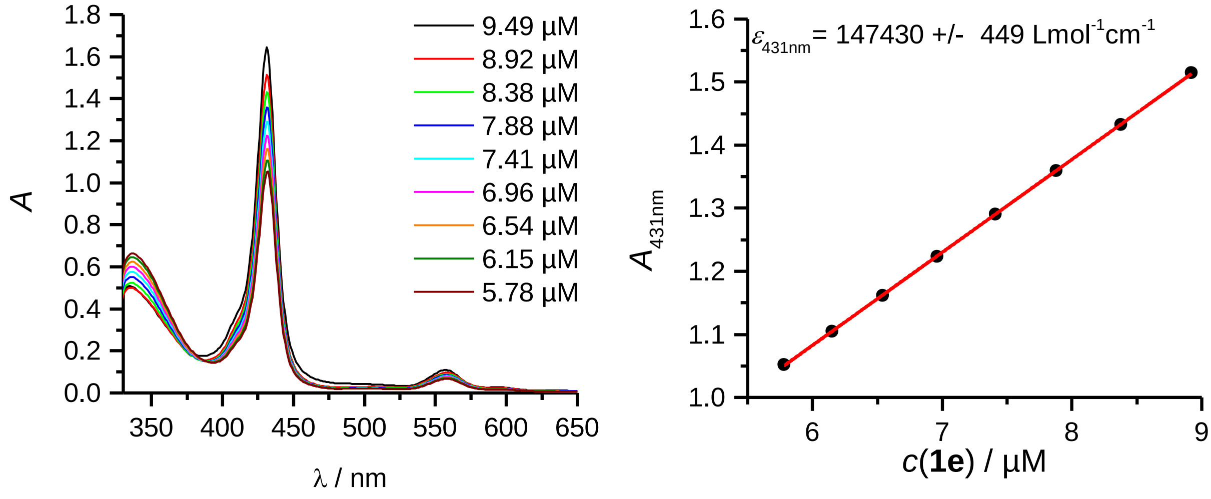

Figure S16.1: UV–vis spectra of completely paramagnetic solutions with different concentrations of porphyrin **1e** in acetone (left) and the plotting of the absorbance at

431 nm as a function of the concentration of **1e**. The extinction coefficient at 431 nm in the completely paramagnetic state was determined as 147430 $Lmol^{-1}cm^{-1}$ (right).

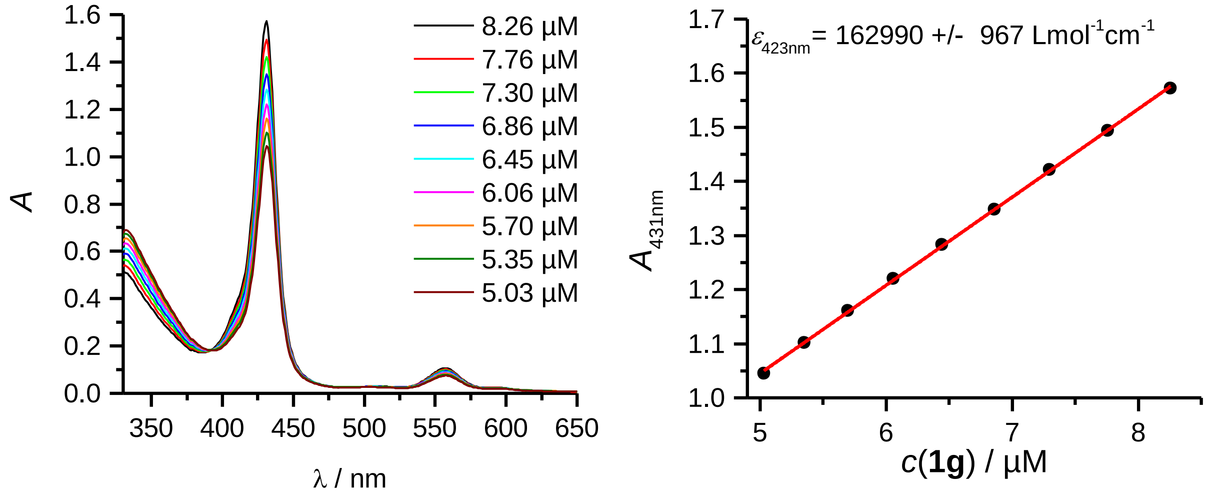

Figure S16.2: UV–vis spectra of completely paramagnetic solutions with different concentrations of porphyrin **1g** in acetone (left) and the plotting of the absorbance at 431 nm as a function of the concentration of **1g**. The extinction coefficient at 431 nm in the completely paramagnetic state was determined as 162990 $Lmol^{-1}cm^{-1}$ (right).

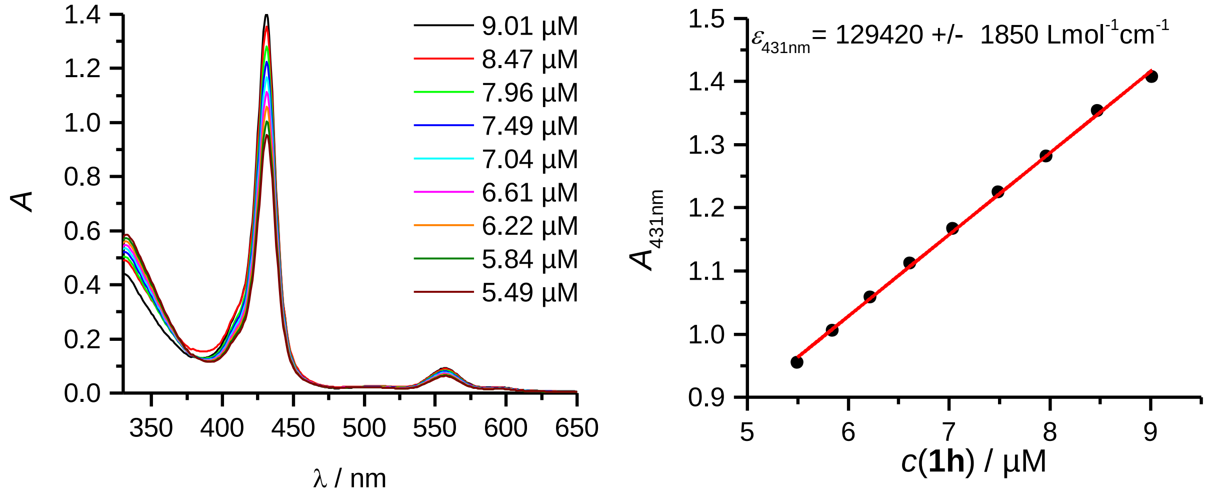

Figure S16.3: UV–vis spectra of completely paramagnetic solutions with different concentrations of porphyrin **1h** in acetone (left) and the plotting of the absorbance at 431 nm as a function of the concentration of **1h**. The extinction coefficient at 431 nm in the completely paramagnetic state was determined as 129420 $Lmol^{-1}cm^{-1}$ (right).

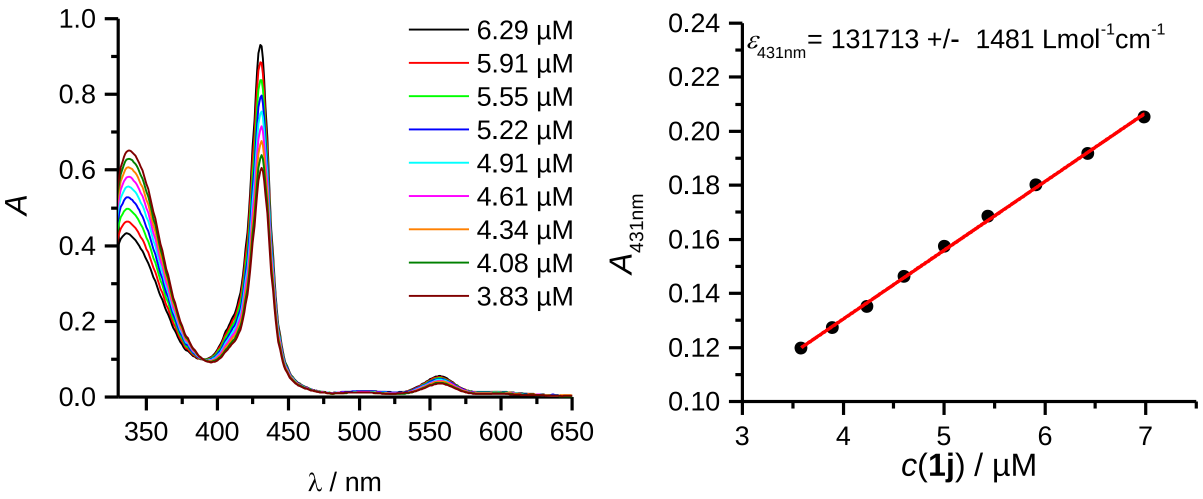

Figure S16.4: UV–vis spectra of completely paramagnetic solutions with different concentrations of porphyrin **1j** in acetone (left) and the plotting of the absorbance at 431 nm as a function of the concentration of **1j**. The extinction coefficient at 431 nm in the completely paramagnetic state was determined as 131713 $Lmol^{-1}cm^{-1}$ (right).

Determination of the switching efficiency by UV–vis spectroscopy

The percentage of paramagnetic record player molecules was determined from UV–vis spectra and calculated by equation [7]:

$$\%\mathbf{para} = \mathbf{100} - \%\mathbf{dia}$$

$$= \mathbf{100} - \left(\frac{\boldsymbol{A_{obs.}} - \boldsymbol{\varepsilon}(\mathbf{para}) \times \boldsymbol{c}}{\boldsymbol{c} \times \boldsymbol{\varepsilon}(\mathbf{dia}) - \boldsymbol{c} \times \boldsymbol{\varepsilon}(\mathbf{para})} \times \mathbf{100} \right) \qquad \text{(Equation S2).}$$

%para	percentage of paramagnetic record player molecules in %.
%dia	percentage of diamagnetic record player molecules in %.
$A_{obs.}$	observed absorbance at λ_{max} of the paramagnetic Soret peak.
ε(para)	extinction coefficient of completely paramagnetic record player molecules at λ_{max} of the paramagnetic Soret peak in $Lmol^{-1}cm^{-1}$.
ε(dia)	extinction coefficient of completely diamagnetic record player molecules at λ_{max} of the paramagnetic Soret peak in $Lmol^{-1}cm^{-1}$.
c	molar concentration of record player molecules in $molL^{-1}$.

DFT calculations on disulfide 1g

General: All calculations were performed with Turbomole7.2 [8], the m4 grid (in Turbomole nomenclature) and the usage of resolution-of-identity (RI) with multipole accelerated RI-J (marij) to speed up the calculations.

All energy values are based on geometry optimizations using density functional theory with the standard GGA functional PBE [9] and Ahlrichs double zeta basis def2SVP [9] followed by single point calculations the hybrid B3LYP [11] (parametrization as implemented in Turbomole7.2) and the triple zeta basis def2TZVP [11]. The B3LYP/def2TZVP/PBE/DZ level of theory performed very well for the prediction of the association enthalpies of pyridines to Ni(II)-pentafluorophenylporphyrins and is therefore a good assumption to describe record player-like systems quantum chemically [5].

Results: The conformational analysis of the disulfide record player system **1g** reveals that the double coordinated *trans* state is favored by more than 20 kcal mol^{-1} (see Table S2) over the decoordinated species. Because of the large difference in energy the equilibrium is strongly driven towards the paramagnetic state which explains the UV/vis measurements (*trans* isomer >80% paramagnetic).

Table S2: Relative energies of geometry optimizations (PBE/def2SVP) and single point calculations (B3LYP/def2TZVP//PBE/def2SVP) of disulfide record player **1g** configurations.

State of **1g \ ** level of theory	PBE/def2SVP kcal mol^{-1}	B3LYP/def2TZVP//PBE/def2SVP kcal mol^{-1}
cis coordinated (quintet)	31.0	29.2
trans decoordinated (singlet)	8.9	21.5
trans coordinated (quintet)	0.0	0.0

Coordinates of PBE/def2SVP structures

Disulfide recordplayer **1g** *cis* coordinated (quintet)

$E_{PBE/def2SVP}$ = -11773.36882575

NImag = 0

C	2.9700348	-1.2016689	0.8744337
C	3.3199658	-2.3254505	1.6541349
C	4.1651997	-3.3238264	1.1313872
C	4.6045645	-3.2450756	-0.2029502
C	4.2990883	-2.0893605	-0.9568745
C	3.4959007	-1.0738782	-0.4168422
C	5.3273116	-4.3946650	-0.8292898
C	4.6271602	-5.5933251	-1.1479515
C	5.3367161	-6.6631398	-1.7331487
C	6.7080334	-6.5596659	-2.0096650
C	7.3927048	-5.3726106	-1.7090787
C	6.7027135	-4.2999539	-1.1256098
C	3.1474427	-5.7214268	-0.9123143
C	2.2810049	-5.0524308	-1.8058707
C	2.7371351	-4.2534342	-2.9294439
C	1.6242694	-3.6848135	-3.4892980
C	0.4846617	-4.1857539	-2.7407289
N	0.9038474	-5.0168505	-1.7263424
C	-0.8659499	-3.8957125	-3.0477300
C	-1.9798847	-4.5416387	-2.4652061
C	-3.3476521	-4.3803690	-2.9255565
C	-4.1264059	-5.1827385	-2.1319158
C	-3.2325896	-5.8136960	-1.1777091
N	-1.9309186	-5.4283895	-1.4149282
Ni	-0.2850065	-5.8392676	-0.2624419
N	-0.4516276	-4.1769983	0.9540489
C	-1.6311696	-3.5406004	1.1344380
C	-1.7472654	-2.4100708	1.9488019
C	-0.6169077	-1.9172251	2.6288881
C	0.6181967	-2.5908858	2.4496550
C	0.6369078	-3.7123329	1.5857050
C	-3.6596209	-6.6345556	-0.1114478
C	-2.8116389	-7.1953419	0.8686028
C	-3.2794742	-7.9859100	1.9937501
C	-2.1703710	-8.3199163	2.7247225
C	-1.0271822	-7.7526925	2.0295582
N	-1.4418086	-7.0673811	0.9093002
C	0.3158633	-7.9248664	2.4312338
C	1.4398030	-7.4513432	1.7142660
N	1.3988535	-6.6767799	0.5761590
C	2.7124576	-6.4867504	0.1941570
C	3.6057706	-7.1647009	1.1165220
C	2.8137901	-7.7632523	2.0625989
N	1.6981229	-2.3586410	3.3419910
N	2.9057710	-2.4088603	3.0136558
H	-5.2098540	-5.3357317	-2.1878600
H	-3.6677621	-3.7581857	-3.7688608
H	-4.3227307	-8.2524845	2.1958513
H	-2.1305269	-8.9065054	3.6487484
H	4.6981298	-7.1880222	1.0380372
H	3.1323094	-8.3809759	2.9093811
H	1.5881858	-3.0036667	-4.3447142
H	3.7792405	-4.1469901	-3.2483014
C	-1.1544047	-2.7737604	-3.9907228
C	-5.1304149	-6.8700173	0.0280561
C	0.5792744	-8.6844278	3.6913840
H	7.2363969	-3.3695089	-0.8771852
H	8.4679536	-5.2808111	-1.9257333
H	7.2397014	-7.4069840	-2.4689112
H	4.7907966	-7.5853202	-1.9849088
H	-2.5035605	-3.9523524	0.6068157
H	1.5611361	-4.2912426	1.4504816
H	-2.7262656	-1.9193283	2.0571995
H	4.4226757	-4.1916213	1.7567630
H	4.6637969	-2.0036366	-1.9907539
H	3.2442632	-0.1942124	-1.0256429
H	2.3116908	-0.4309844	1.3008760
C	-0.8475007	-2.7925379	-5.3653141
C	-1.0862261	-1.6811739	-6.1956224
C	-1.6733106	-0.5253700	-5.6533401
C	-2.0204688	-0.4873325	-4.2927156
C	-1.7486408	-1.5992083	-3.4832016
F	-0.3020918	-3.8805550	-5.9183612
F	-0.7680484	-1.7194335	-7.4899500
F	-1.8905219	0.5452192	-6.4242645
F	-2.5867328	0.6126083	-3.7774307
F	-2.0628134	-1.5224908	-2.1792580
C	1.0703813	-8.0281668	4.8381341
C	1.3321755	-8.7188299	6.0334101
C	1.1033577	-10.1044889	6.0950057
C	0.6169518	-10.7869386	4.9664305
C	0.3629178	-10.0738265	3.7821903
F	1.2971008	-6.7096204	4.8079157
F	1.7911676	-8.0713511	7.1075474
F	1.3480944	-10.7712223	7.2237156
F	0.4063177	-12.1047795	5.0251317
F	-0.0921690	-10.7527298	2.7228942
C	-5.9026952	-6.0903951	0.9095435
C	-7.2854194	-6.2897205	1.0574547
C	-7.9200386	-7.2954887	0.3069857
C	-7.1738786	-8.0872140	-0.5834457
C	-5.7911663	-7.8671621	-0.7135977
F	-5.3184359	-5.1222722	1.6300416
F	-7.9985759	-5.5351645	1.8981641
F	-9.2319798	-7.4959512	0.4378623
F	-7.7813764	-9.0402769	-1.2947984
F	-5.1040212	-8.6309163	-1.5687491
C	-2.9732285	1.2108523	0.8701604
C	-3.3220671	2.3379330	1.6455199
C	-4.1658624	3.3354333	1.1187494
C	-4.6041503	3.2526735	-0.2157406
C	-4.2991868	2.0941152	-0.9654957
C	-3.4981746	1.0792753	-0.4210628
C	-5.3260059	4.4003117	-0.8467733
C	-4.6254851	5.5976477	-1.1695160
C	-5.3343714	6.6654752	-1.7590577
C	-6.7054962	6.5611775	-2.0362802
C	-7.3905247	5.3752799	-1.7319671
C	-6.7011820	4.3047071	-1.1439145
C	-3.1459689	5.7263329	-0.9330876
C	-2.2786087	5.0566238	-1.8252347
C	-2.7333318	4.2557255	-2.9480477
C	-1.6196604	3.6864542	-3.5057476
C	-0.4810102	4.1887690	-2.7566069
N	-0.9015882	5.0212217	-1.7439808
C	0.8700828	3.8990697	-3.0617732
C	1.9828883	4.5472855	-2.4797313
C	3.3506880	4.3895541	-2.9411192
C	4.1275382	5.1963750	-2.1500626
C	3.2327680	5.8256492	-1.1956171
N	1.9322450	5.4351160	-1.4304584
Ni	0.2855047	5.8439962	-0.2794162
N	0.4504174	4.1831136	0.9386235
C	1.6295764	3.5465693	1.1208547
C	1.7450380	2.4188905	1.9392643
C	0.6144851	1.9293738	2.6215608
C	-0.6202685	2.6029859	2.4399813
C	-0.6383078	3.7212333	1.5719356
C	3.6582996	6.6489601	-0.1305710
C	2.8098697	7.2041066	0.8523679
C	3.2767045	7.9900229	1.9812278
C	2.1679036	8.3138628	2.7172765
C	1.0254974	7.7472635	2.0203282
N	1.4406103	7.0711536	0.8946764
C	-0.3176940	7.9118253	2.4247511
C	-1.4409644	7.4447556	1.7024144
N	-1.3989140	6.6788491	0.5586868
C	-2.7121192	6.4900599	0.1748712
C	-3.6063096	7.1627343	1.1002326
C	-2.8152943	7.7541303	2.0516932
N	-1.7003534	2.3749276	3.3332316
N	-2.9079658	2.4255708	3.0048184
H	5.2104044	5.3528916	-2.2075634
H	3.6719744	3.7667657	-3.7835394
H	4.3192195	8.2598767	2.1827991
H	2.1278780	8.8941591	3.6452469
H	-4.6985735	7.1869650	1.0204209
H	-3.1345233	8.3659498	2.9025115
H	-1.5822994	3.0038035	-4.3599374
H	-3.7751215	4.1482529	-3.2676577
C	1.1602167	2.7780260	-4.0053265
C	5.1280686	6.8933741	0.0039476
C	-0.5828437	8.6476188	3.6984188
H	-7.2351975	3.3752321	-0.8925807
H	-8.4655825	5.2827667	-1.9492716
H	-7.2367227	7.4069112	-2.4989451
H	-4.7881204	7.5866850	-2.0136421
H	2.5019250	3.9559176	0.5912508
H	-1.5621568	4.3001989	1.4348157
H	2.7236585	1.9277326	2.0491908
H	-4.4228969	4.2055592	1.7410455
H	-4.6629061	2.0054484	-1.9994770
H	-3.2475893	0.1968474	-1.0261699
H	-2.3164032	0.4406784	1.2998914
C	0.8565764	2.7997557	-5.3805497
C	1.0971869	1.6902081	-6.2126820
C	1.6820494	0.5328751	-5.6712038
C	2.0256177	0.4918686	-4.3096966
C	1.7528240	1.6023619	-3.4985673
F	0.3129853	3.8892602	-5.9324054
F	0.7828233	1.7317795	-7.5078332
F	1.9009851	-0.5361349	-6.4436029
F	2.5896165	-0.6097051	-3.7952195
F	2.0642173	1.5232258	-2.1941126
C	-1.0714345	7.9657865	4.8313519
C	-1.3340996	8.6299270	6.0412234
C	-1.1088101	10.0145472	6.1320992
C	-0.6253399	10.7222380	5.0178947
C	-0.3703430	10.0352889	3.8184156
F	-1.2942055	6.6474588	4.7732380
F	-1.7903194	7.9583680	7.1015948
F	-1.3541768	10.6562994	7.2750307
F	-0.4180632	12.0390756	5.1047061
F	0.0819556	10.7379055	2.7734633
C	5.9079146	6.1225512	0.8865124
C	7.2899526	6.3307179	1.0286780
C	7.9161429	7.3365414	0.2712457
C	7.1623196	8.1196435	-0.6203559
C	5.7805234	7.8907694	-0.7447756
F	5.3318510	5.1545439	1.6136492
F	8.0103885	5.5843276	1.8704860
F	9.2273078	7.5453708	0.3967663
F	7.7617468	9.0729093	-1.3382793
F	5.0860520	8.6463171	-1.6012991
S	-0.8805523	-0.5677734	3.7617470
S	0.8776745	0.5843946	3.7597682

Disulfide recordplayer **1g** *trans* decoordinated (singlet)

$E_{PBE/def2SVP}$ = -11773.40397325

NImag = 0

C	2.6662600	1.5093997	-3.7249633
C	1.6263925	1.3585517	-2.7970153
C	1.8932082	0.8899845	-1.4917216
C	3.2567283	0.5863474	-1.1810981
C	4.2029254	0.7290551	-2.2372050
N	3.9448241	1.1811659	-3.4631582
N	3.8626605	0.1593869	0.0051795
N	3.1822928	0.1450342	1.0771827
C	3.9329989	-0.3006273	2.2015513
C	3.2055989	-0.5894734	3.3763672
C	3.8631642	-1.0528748	4.5369597
C	5.2706057	-1.1949775	4.4938631
C	5.9983509	-0.8922649	3.3309468
C	5.3398967	-0.4523421	2.1822294
C	3.1518974	-1.3760541	5.8094053
C	1.9578394	-2.1516234	5.8801372
C	1.4070070	-2.4502911	7.1474352
C	1.9934575	-1.9929070	8.3338567
C	3.1538777	-1.2079886	8.2676929
C	3.7176302	-0.9118417	7.0216229
C	1.2260185	-2.6756955	4.6812606
C	1.7746034	-3.7051888	3.9105134
C	3.0817971	-4.2597952	4.1671329
C	3.3249761	-5.1637659	3.1724584
C	2.1478712	-5.1823925	2.3372405
N	1.1893250	-4.2928592	2.7959220
C	2.0150036	-6.0460554	1.2453741
C	0.8144563	-6.1752247	0.5441231
C	0.6346975	-7.1205901	-0.5316678
C	-0.6695018	-7.0208371	-0.9230374
C	-1.2691479	-5.9973008	-0.1000220
N	-0.3545800	-5.4743556	0.8006471
Ni	-0.6280624	-4.0125763	2.0935394
N	-0.9054972	-2.5674127	3.3967818
C	-2.0076381	-1.7364573	3.5236510
C	-1.8215845	-0.7818059	4.5893002
C	-0.5894508	-1.0249449	5.1232663
C	-0.0338970	-2.1381005	4.3891432
C	-2.5933897	-5.5795819	-0.2618152
C	-3.1208748	-4.4982443	0.4497737
C	-4.4552758	-3.9949475	0.2254668
C	-4.6012422	-2.9047595	1.0329065
C	-3.3711634	-2.7741464	1.7775555
N	-2.4545295	-3.7474628	1.4066899
C	-3.1871498	-1.8103632	2.7733325
H	-1.1836846	-7.5679463	-1.7195218
H	1.4163398	-7.7736349	-0.9327138
H	-5.1790422	-4.4207964	-0.4763704
H	-5.4641100	-2.2373179	1.1187625
H	-0.0834631	-0.4965174	5.9353540
H	-2.5498456	-0.0238179	4.8927829
H	4.2104501	-5.7900315	3.0224128
H	3.7244984	-3.9760014	5.0058596
C	3.1872536	-6.8778499	0.8410852

C	-3.4543765	-6.2914575	-1.2528262
C	-4.3067295	-0.8774383	3.0992619
H	4.6143587	-0.2758286	6.9726022
H	3.6193544	-0.8198432	9.1864574
H	1.5410022	-2.2454250	9.3047873
H	0.4949340	-3.0650323	7.1886710
H	2.1183752	-0.4369507	3.3587842
H	5.8009395	-1.5674555	5.3836043
H	7.0929581	-1.0130956	3.3275138
H	5.8754019	-0.2195049	1.2516370
H	5.2446508	0.4516415	-1.9944185
H	0.5897023	1.5811570	-3.0922556
H	2.4416925	1.8860283	-4.7384378
C	-5.5062864	-1.3548321	3.6683171
C	-6.5714886	-0.4969837	3.9917778
C	-6.4422099	0.8832542	3.7634219
C	-5.2496534	1.3910437	3.2222859
C	-4.2060766	0.5126414	2.8870351
F	-5.6573685	-2.6594931	3.9215210
F	-7.6969338	-0.9824309	4.5220855
F	-7.4433369	1.7108044	4.0659871
F	-5.1267068	2.7095029	3.0276069
F	-3.0875951	1.0323845	2.3656421
C	-3.9348350	-7.5902831	-0.9911612
C	-4.7417771	-8.2811330	-1.9115455
C	-5.0823178	-7.6684554	-3.1300422
C	-4.6158943	-6.3743348	-3.4197233
C	-3.8089386	-5.7059863	-2.4839808
F	-3.6255483	-8.1978515	0.1587802
F	-5.1876499	-9.5092869	-1.6368358
F	-5.8478470	-8.3138381	-4.0101533
F	-4.9361612	-5.7937555	-4.5789325
F	-3.3744527	-4.4757869	-2.7925570
C	4.2742822	-6.3090949	0.1499615
C	5.3909282	-7.0696904	-0.2347601
C	5.4311192	-8.4400564	0.0772030
C	4.3599992	-9.0367433	0.7651824
C	3.2542590	-8.2532390	1.1383216
F	4.2583145	-5.0062919	-0.1709870
F	6.4052340	-6.5058467	-0.8943676
F	6.4839024	-9.1743044	-0.2820386
F	4.4019667	-10.3379224	1.0609457
F	2.2499883	-8.8457728	1.7923066
S	0.4420710	0.7955065	-0.4621055
C	1.6725082	-3.9588675	-2.1729671
C	1.5865682	-3.0583989	-1.1011483
C	0.4713939	-2.2045146	-0.9842026
C	-0.5499983	-2.3210566	-1.9782452
C	-0.3253743	-3.2638714	-3.0217523
N	0.7391919	-4.0585661	-3.1365290
N	-1.7949433	-1.6940023	-2.0906911
N	-2.1002080	-0.7750578	-1.2698351
C	-3.4127058	-0.2686893	-1.4677600
C	-3.7263466	0.9428301	-0.8154195
C	-4.9952284	1.5420757	-0.9749444
C	-5.9447676	0.8759590	-1.7880514
C	-5.6426018	-0.3435528	-2.4167410
C	-4.3812390	-0.9212667	-2.2672679
C	-5.3893927	2.8311071	-0.3299692
C	-4.5561230	3.9882835	-0.2645818
C	-5.0761653	5.1751663	0.3010059
C	-6.3742207	5.2422089	0.8212578
C	-7.1866542	4.1002168	0.7865607
C	-6.6959363	2.9234415	0.2109180
C	-3.1338421	4.0375559	-0.7375561
C	-2.8288151	3.9641710	-2.1021069
C	-3.8146080	3.6945303	-3.1220531
C	-3.1474757	3.6508490	-4.3134661
C	-1.7690669	3.9694261	-4.0247187
N	-1.5685680	4.1249927	-2.6609979
C	-0.8245087	4.2453187	-5.0200787
C	0.4434016	4.7513218	-4.7106837
C	1.3430834	5.2922363	-5.7019858
C	2.4692821	5.6854110	-5.0335729
C	2.2708955	5.3298493	-3.6483759
N	0.9980818	4.8164305	-3.4450559
Ni	0.1326309	4.4180750	-1.7387740
N	-0.7571993	4.2002255	-0.0198926
C	-0.1707049	4.1306289	1.2335392
C	-1.1703994	4.1082907	2.2718762
C	-2.3859987	4.1311394	1.6488289
C	-2.1233468	4.1662997	0.2277455
C	3.2981186	5.3291674	-2.6981874
C	3.0902936	4.8460380	-1.4000990
C	4.1614081	4.6082873	-0.4617289
C	3.5774536	4.1838661	0.6989238
C	2.1513197	4.2039458	0.4739050
N	1.8608062	4.5466171	-0.8381852
C	1.2036445	4.0495425	1.4914627
H	3.3713543	6.1539884	-5.4401341
H	1.1211120	5.3784358	-6.7709015
H	5.2266612	4.7345611	-0.6812906
H	4.0622793	3.8886734	1.6350228
H	-3.3795753	4.0843723	2.1009429
H	-0.9613029	4.0713136	3.3457772
H	-3.5543742	3.4584840	-5.3112418
H	-4.8821767	3.5471601	-2.9354189
C	-1.1727570	4.0437292	-6.4562715
C	4.6656151	5.7780102	-3.0869996
C	1.6673800	3.8592890	2.8954854
H	-7.3329692	2.0260394	0.1985648
H	-8.2017968	4.1213937	1.2111881
H	-6.7448145	6.1825472	1.2566027
H	-4.4305054	6.0661720	0.3258474
H	-2.9535291	1.3952387	-0.1800968
H	-6.9329382	1.3339297	-1.9466568
H	-6.4054439	-0.8369236	-3.0392856
H	-4.1093143	-1.8707166	-2.7481203
H	-1.1179259	-3.3437814	-3.7863094
H	2.3986314	-2.9996888	-0.3614285
H	2.5549226	-4.6165843	-2.2593424
C	2.3846799	4.8707383	3.5695246
C	2.8434992	4.7079192	4.8876615
C	2.5750175	3.5092676	5.5692715
C	1.8456614	2.4931259	4.9306033
C	1.4004171	2.6729743	3.6106383
F	2.6421397	6.0324102	2.9591662
F	3.5213155	5.6840719	5.4970476
F	3.0059038	3.3373637	6.8195439
F	1.5899839	1.3546033	5.5876158
F	0.7104170	1.6797960	3.0396256
C	5.2321213	6.9377120	-2.5186883
C	6.5232591	7.3746573	-2.8589986
C	7.2797102	6.6387125	-3.7875438
C	6.7436704	5.4757773	-4.3673198
C	5.4496448	5.0570853	-4.0121200
F	4.5345215	7.6579987	-1.6337399
F	7.0334179	8.4814871	-2.3125466
F	8.5060106	7.0441823	-4.1186877
F	7.4677333	4.7758504	-5.2438848
F	4.9743389	3.9418711	-4.5724144
C	-0.5663232	3.0052557	-7.1920284
C	-0.8541052	2.7921921	-8.5504267
C	-1.7706709	3.6365569	-9.2015507
C	-2.3883012	4.6832183	-8.4948308
C	-2.0827568	4.8767562	-7.1366510
F	0.3084687	2.1844659	-6.5954658
F	-0.2709204	1.7978522	-9.2231130
F	-2.0535193	3.4448833	-10.4900587
F	-3.2526061	5.4880173	-9.1183591
F	-2.6768982	5.8882565	-6.4937402
S	0.4989670	-1.1137644	0.4261095

Disulfide recordplayer **1g** *trans* coordinated (quintet)

$E_{PBE/def2SVP}$ = -11773.41822439

NImag = 0

C	0.8234184	2.1775404	-3.0890774
C	1.4971428	0.9617443	-3.2068329
C	2.2932226	0.4952471	-2.1370060
C	2.3008880	1.3084433	-0.9564898
C	1.5388368	2.4924056	-0.9121133
N	0.8448108	2.9403042	-1.9616101
N	2.9923341	0.9210325	0.1690560
N	3.8325296	0.0033037	-0.0803281
C	4.4263171	-0.6751303	1.0012252
C	3.8508490	-0.6604786	2.2916447
C	4.4782711	-1.3371964	3.3501391
C	5.6877071	-2.0269264	3.0919131
C	6.2434825	-2.0606071	1.8026667
C	5.6139338	-1.3931336	0.7464443
C	3.9240152	-1.2528775	4.7347127
C	2.6351052	-1.7422817	5.0867805
C	2.1755297	-1.5413425	6.4087267
C	2.9590437	-0.8897169	7.3699163
C	4.2397268	-0.4321622	7.0270734
C	4.7110315	-0.6163350	5.7209489
C	1.7582869	-2.5422008	4.1647059
C	2.1571778	-3.8655325	3.8628716
C	3.3880416	-4.4705647	4.3398870
C	3.4273199	-5.7419157	3.8306772
C	2.2037059	-5.9203731	3.0716787
N	1.4424772	-4.7721378	3.1061931
C	1.8570884	-7.1188224	2.4074647
C	0.6220876	-7.3672996	1.7716134
C	0.2655543	-8.6292687	1.1473857
C	-1.0218354	-8.4922521	0.6994611
C	-1.4432521	-7.1463963	1.0494447
N	-0.4282175	-6.4806709	1.6966580
Ni	-0.4022824	-4.4888371	2.2145801
N	-0.5121588	-2.6628031	3.1558162
C	-1.5684309	-1.7825622	3.0991534
C	-1.1846598	-0.4964045	3.6532561
C	0.1092442	-0.6249417	4.0864839
C	0.5127655	-1.9885212	3.7872316
C	-2.7389310	-6.6348180	0.8119484
C	-3.1894599	-5.3604117	1.2222223
C	-4.5508320	-4.8833845	1.0543709
C	-4.5944174	-3.6237362	1.5892258
C	-3.2524310	-3.3233310	2.0570790
N	-2.4200356	-4.3993950	1.8412086
C	-2.8654079	-2.0874020	2.6222674
H	-1.6407244	-9.2451266	0.1993540
H	0.9090058	-9.5137191	1.0833704
H	-5.3725897	-5.4425388	0.5945280
H	-5.4620480	-2.9594991	1.6643770
H	0.7390906	0.1331736	4.5639137
H	-1.8214459	0.3937683	3.7009491
H	4.2040711	-6.5003152	3.9771716
H	4.1172836	-3.9873565	4.9981584
H	5.7040928	-0.2342994	5.4373848
H	4.8696055	0.0770008	7.7722827
H	2.5713153	-0.7510032	8.3905701
H	1.1827221	-1.9295077	6.6822379
H	2.9265142	-0.0873065	2.4527264
H	6.1885461	-2.5568594	3.9164377
H	7.1773970	-2.6141065	1.6235616
H	6.0326088	-1.3950884	-0.2704923
S	3.2463959	-0.9785615	-2.1222635
C	1.3607829	-4.4064905	-0.1397515
C	1.8983777	-4.0115454	-1.3673012
C	1.2405986	-3.0348754	-2.1336720
C	-0.0098965	-2.5491836	-1.6610870
C	-0.4619600	-2.9857649	-0.3919174
N	0.2167767	-3.8763886	0.3524056
N	-0.7796331	-1.7464756	-2.5117571
N	-1.6019592	-0.9501031	-1.9695672
C	-2.4976811	-0.3317896	-2.8742386
C	-3.3209012	0.6835846	-2.3424885
C	-4.3139337	1.2923072	-3.1353715
C	-4.4584106	0.8639244	-4.4737503
C	-3.6210605	-0.1296499	-5.0145253
C	-2.6393617	-0.7306575	-4.2262786
C	-5.2493062	2.3062693	-2.5652442
C	-4.7986843	3.5102260	-1.9546979
C	-5.7520487	4.3946378	-1.4032674
C	-7.1241630	4.1148344	-1.4528579
C	-7.5697127	2.9369596	-2.0708313
C	-6.6372693	2.0480550	-2.6211106
C	-3.3535364	3.9091061	-1.9275063
C	-2.7463631	4.2591157	-3.1554562
C	-3.4403045	4.2664042	-4.4309330
C	-2.5162009	4.6102176	-5.3818836
C	-1.2689162	4.8481462	-4.6793381
N	-1.4306841	4.6363830	-3.3276999
C	-0.0709980	5.2579714	-5.3071206
C	1.1052505	5.6438867	-4.6292755
C	2.2808195	6.1857195	-5.2880439
C	3.1617512	6.5257632	-4.2961139
C	2.5265173	6.1761098	-3.0369550
N	1.2785766	5.6416510	-3.2635205
Ni	0.0076634	4.8205103	-1.8724820
N	-1.4231014	4.4254292	-0.4483844
C	-1.2149579	4.3285095	0.9088228
C	-2.3867472	3.7734791	1.5621029
C	-3.3195886	3.5617405	0.5797628
C	-2.7046922	3.9712001	-0.6723471
C	3.1075040	6.3990855	-1.7683347
C	2.4894098	6.0979197	-0.5338565
C	3.1123670	6.3058192	0.7613840
C	2.2314661	5.8388422	1.7011380
C	1.0729662	5.3491343	0.9764265
N	1.2427994	5.5318991	-0.3796679
C	-0.0471941	4.7447133	1.5891365
H	4.1455747	6.9944028	-4.4066534
H	2.4019697	6.3189999	-6.3686687
H	4.1005715	6.7461338	0.9319778
H	2.3510972	5.8366658	2.7901673
H	-4.3325812	3.1595891	0.6816962
H	-2.4895564	3.5840727	2.6364151
H	-2.6722300	4.7151781	-6.4608645
H	-4.5036397	4.0487799	-4.5741502
H	-6.9813640	1.1095976	-3.0827925
H	-8.6444448	2.7040669	-2.1176547
H	-7.8447205	4.8227805	-1.0158700
H	-5.3974729	5.3298014	-0.9436464
H	-3.1938994	0.9534338	-1.2857762
H	-5.2361844	1.3237508	-5.1029983
H	-3.7505082	-0.4383301	-6.0634167
H	-1.9816521	-1.5225748	-4.6114351
S	2.0505893	-2.3004119	-3.5426836
H	2.8543318	-4.4316090	-1.7111123
H	1.8684723	-5.1573905	0.4823496
H	-1.4129336	-2.5947809	0.0028771
H	1.5289934	3.0766639	0.0187217
H	1.4074374	0.3710669	-4.1293416
H	0.2367604	2.5751484	-3.9308774
C	-0.0479539	5.3031793	-6.8017204
C	-0.6706018	6.3359409	-7.5282364
C	0.6146127	4.2995784	-7.5342376
C	-0.6387898	6.3734033	-8.9331988
C	0.6625642	4.3149798	-8.9381547
C	0.0326203	5.3590752	-9.6378929
C	4.4801330	6.9898331	-1.7311808
C	4.7001356	8.3081192	-1.2841831

C	5.6059834	6.2503311	-2.1452115
C	5.9861466	8.8740096	-1.2479637
C	6.9013483	6.7942163	-2.1199795
C	7.0898144	8.1120710	-1.6687882
C	0.0240252	4.5051555	3.0638556
C	-0.7093375	5.2815678	3.9821390
C	0.8316495	3.4751260	3.5866985
C	-0.6404129	5.0528600	5.3679597
C	0.9191336	3.2295341	4.9665306
C	0.1707913	4.0153649	5.8598341
C	-3.9034919	-1.0183562	2.7232096
C	-4.3912511	-0.5742852	3.9693287
C	-4.4228132	-0.3985372	1.5704225
C	-5.3485540	0.4504869	4.0695253
C	-5.3843025	0.6209305	1.6442984
C	-5.8376848	1.0575712	2.8999661
C	-3.7055830	-7.5197926	0.0919172
C	-4.7878273	-8.1293874	0.7569695
C	-3.5592111	-7.7835861	-1.2845724
C	-5.6953887	-8.9654441	0.0840129

C	-4.4521032	-8.6171355	-1.9792881
C	-5.5245487	-9.2088868	-1.2898099
C	2.8790266	-8.2109349	2.3749717
C	3.0845349	-9.0684014	3.4721600
C	3.6738453	-8.4128796	1.2311048
C	4.0470456	-10.0925970	3.4375530
C	4.6421458	-9.4289822	1.1707220
C	4.8260444	-10.2717198	2.2810489
F	3.6695764	9.0591161	-0.8805713
F	6.1658686	10.1276054	-0.8229271
F	8.3149492	8.6388039	-1.6397608
F	7.9517540	6.0688970	-2.5150376
F	5.4593034	4.9913951	-2.5756440
F	1.5443381	2.6938043	2.7618721
F	-1.4928625	6.2721981	3.5443312
F	1.6848540	2.2342562	5.4343296
F	0.2399283	3.7853785	7.1712854
F	-1.3424722	5.8071828	6.2172828
F	-1.3158087	7.3136970	-6.8830185
F	1.2169820	3.2903408	-6.8897366

F	1.2947364	3.3487196	-9.6101137
F	-1.2363375	7.3638407	-9.6012244
F	0.0696528	5.3854855	-10.9708902
F	4.2253571	-10.8949963	4.4903389
F	2.3596643	-8.9159197	4.5852087
F	5.7424784	-11.2398913	2.2379520
F	5.3873301	-9.5983290	0.0747046
F	3.5201043	-7.6184806	0.1614081
F	-2.5481405	-7.2337881	-1.9675514
F	-4.2922636	-8.8470405	-3.2858096
F	-6.7123555	-9.5326811	0.7390264
F	-4.9743283	-7.9203073	2.0652060
F	-6.3793962	-10.0011473	-1.9386974
F	-3.9469246	-1.1310374	5.1007976
F	-3.9880280	-0.7709054	0.3590860
F	-5.8391917	1.2141077	0.5325542
F	-6.7295272	2.0463504	2.9782961
F	-5.7913912	0.8527664	5.2637432

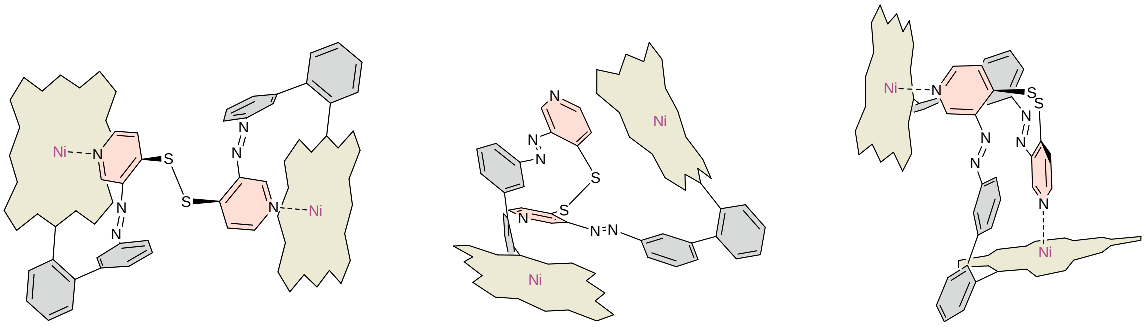

Figure S17: DFT optimized structures for disulfide **1g**, where both azo units are in the coordinated *cis*- (left), decoordinated-*trans*- (middle) or intramolecular coordinated *trans*-state (right). The porphyrin structure was simplified by removing the *meso* substituents, double bonds and nitrogen atoms for clarity.

Switching and stability

A solution of porphyrin **1e**, **1f**, **1h**, **1i**, **1j** in acetone was alternately irradiated with 505 nm and 435 nm into the PSS. UV–vis spectra were recorded after the irradiation cycle and the absorbance of $\lambda_{max.}$ of the paramagnetic (diamagnetic for **1i**) Soret peak was measured (Figure S18.1-S18.8).

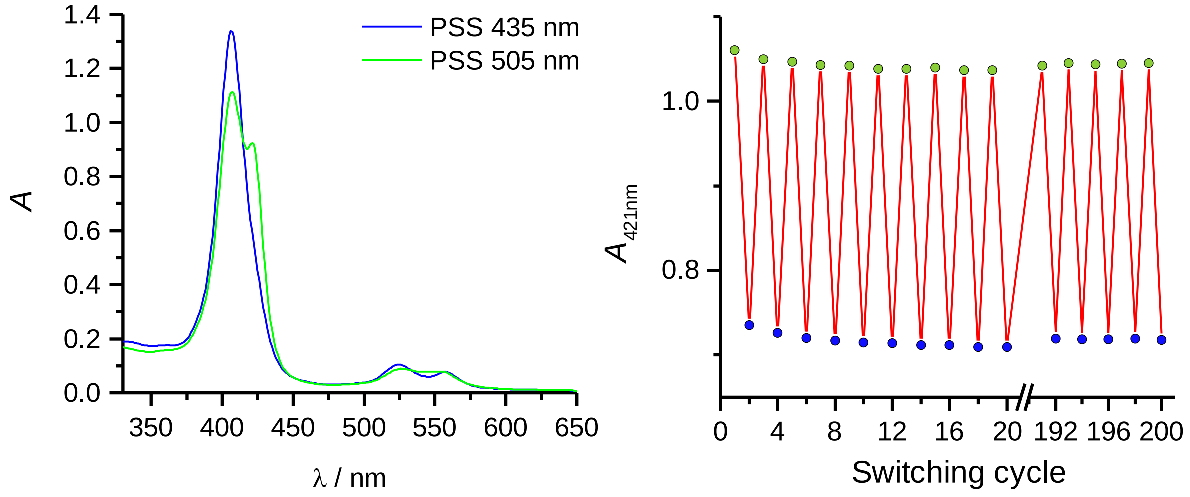

Figure S18.1: UV–vis spectra in the PSS at 505 nm and 435 nm of **1e** (left) and absorbance at 421 nm ($\lambda_{max.}$ paramagnetic Soret peak) of porphyrin **1e** in acetone at 25 °C after irradiation with 505 nm (green) and 435 nm (blue) plotted against the number of switching cycles (right). No degradation was observed within 200 switching processes.

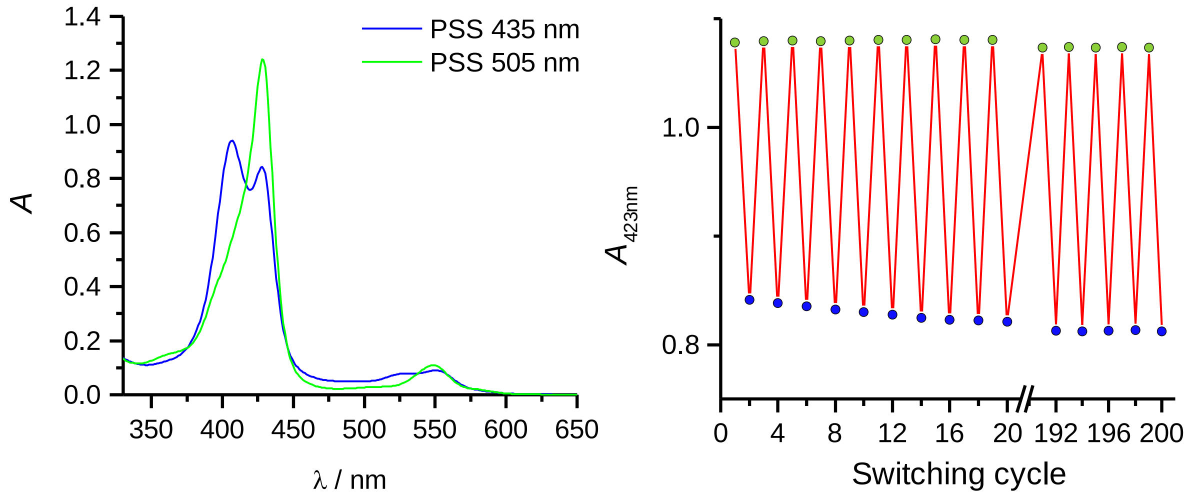

Figure S18.2: UV–vis spectra in the PSS at 505 nm and 435 nm of **1e** (left) and absorbance at 423 nm ($\lambda_{max.}$ paramagnetic Soret peak) of porphyrin **1e** in acetone and addition of Cs_2CO_3 at 25 °C after irradiation with 505 nm (green) and 435 nm (blue) plotted against the number of switching cycles (right). No degradation was observed within 200 switching processes.

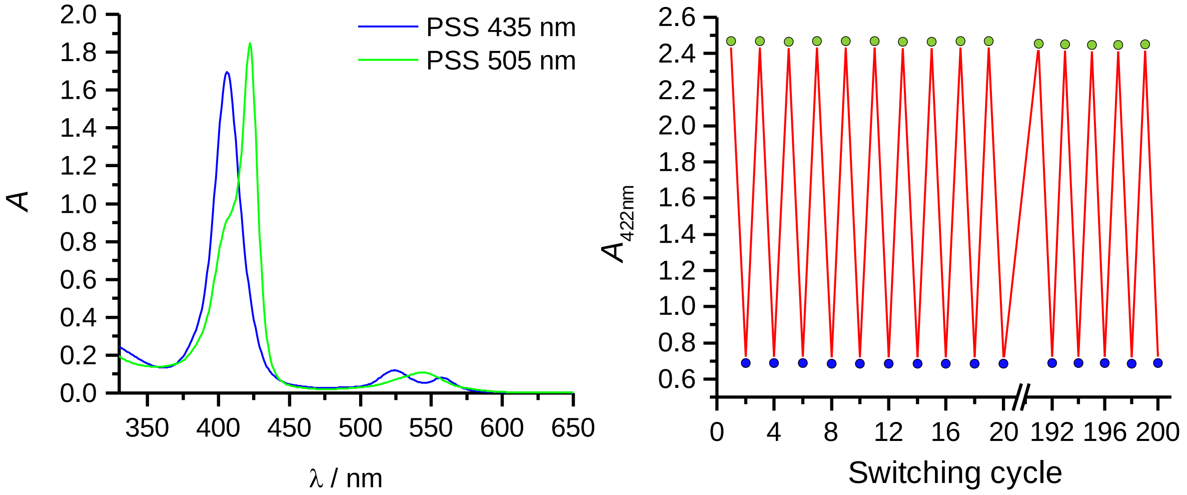

Figure S18.3: UV–vis spectra in the PSS at 505 nm and 435 nm of **1f** (left) and absorbance at 422 nm ($\lambda_{max.}$ paramagnetic Soret peak) of porphyrin **1f** in acetone at 25 °C after irradiation with 505 nm (green) and 435 nm (blue) plotted against the number of switching cycles (right). No degradation was observed within 200 switching processes.

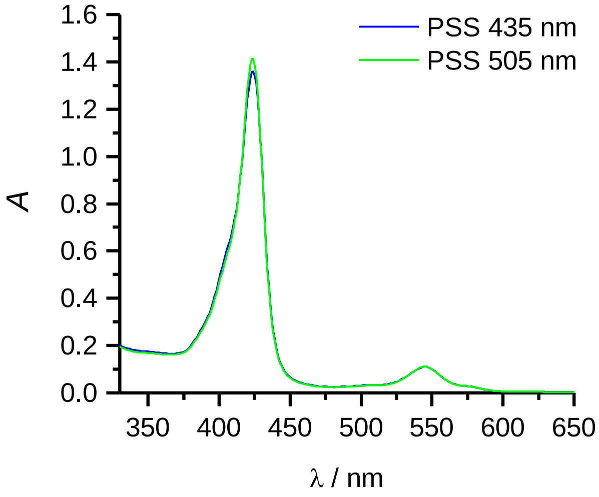

Figure S18.4: UV–vis spectra in the PSS at 505 nm and 435 nm of **1g**. The paramagnetic state is a result of both, the intramolecular coordinating *cis* and *trans*-isomer (Figure S17).

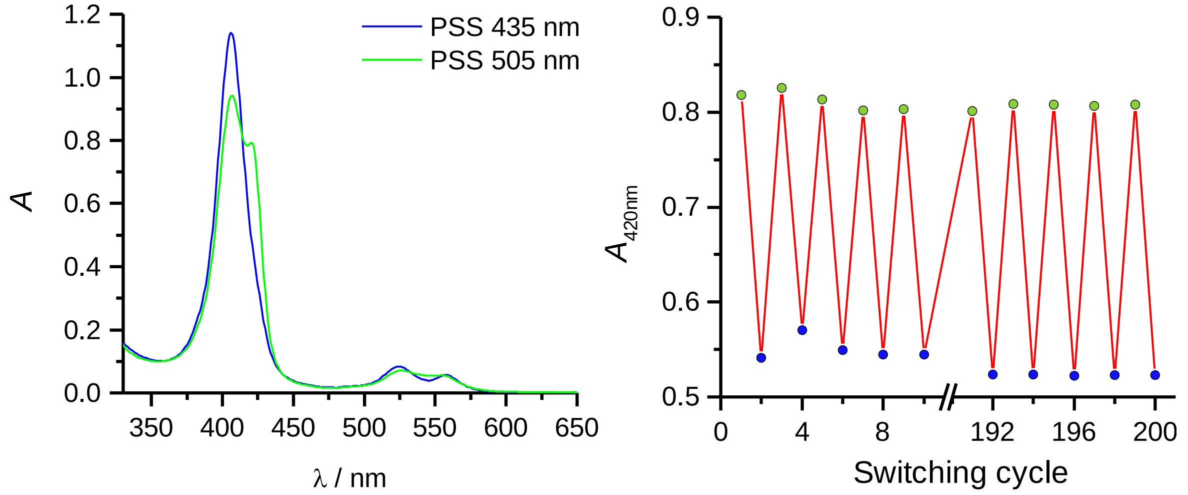

Figure S18.5: UV–vis spectra in the PSS at 505 nm and 435 nm of **1h** (left) and absorbance at 420 nm ($\lambda_{max.}$ paramagnetic Soret peak) of porphyrin **1h** in acetone at 25 °C after irradiation with 505 nm (green) and 435 nm (blue) plotted against the number of switching cycles (right). No degradation was observed within 200 switching processes.

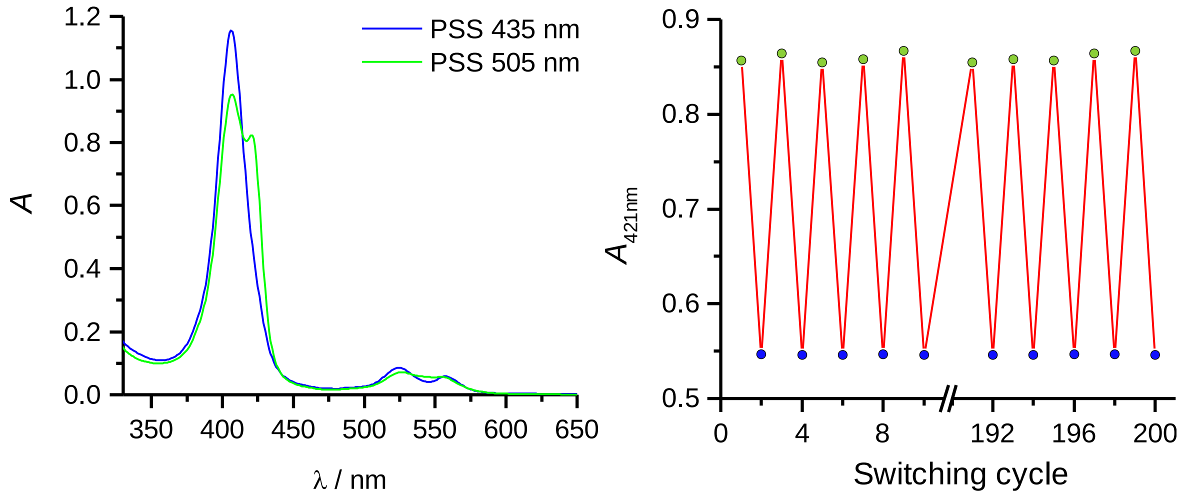

Figure S18.6: UV–vis spectra in the PSS at 505 nm and 435 nm of **1h** (left) and absorbance at 421 nm ($\lambda_{max.}$ paramagnetic Soret peak) of porphyrin **1h** in acetone and addition of Cs_2CO_3 at 25 °C after irradiation with 505 nm (green) and 435 nm (blue) plotted against the number of switching cycles (right). No degradation was observed within 200 switching processes.

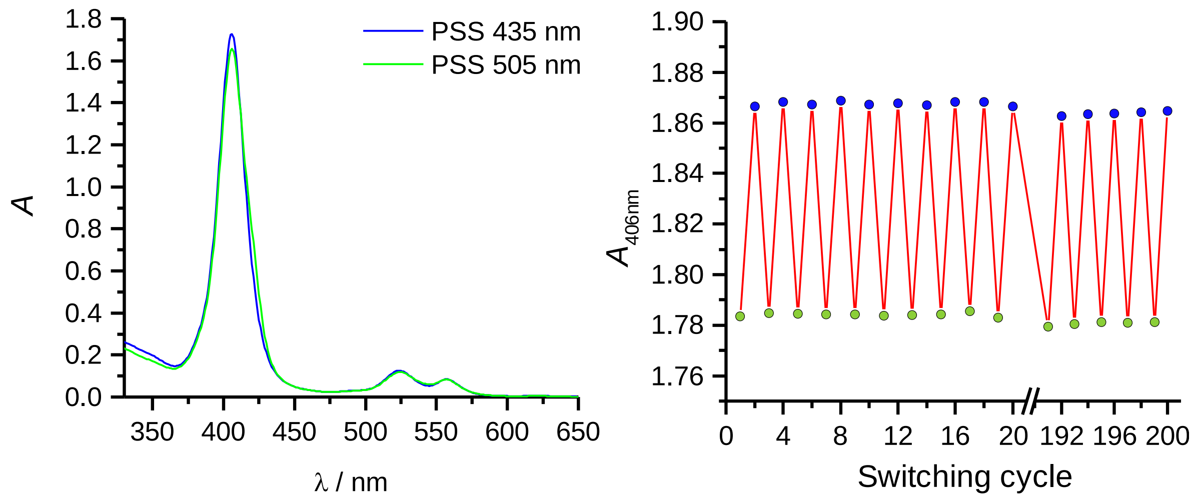

Figure S18.7: Absorbance at 406 nm ($\lambda_{max.}$ paramagnetic Soret peak) of porphyrin **1i** in acetone at 25 °C after irradiation with 505 nm (green) and 435 nm (blue) plotted against the number of switching cycles. No degradation was observed within 200 switching processes.

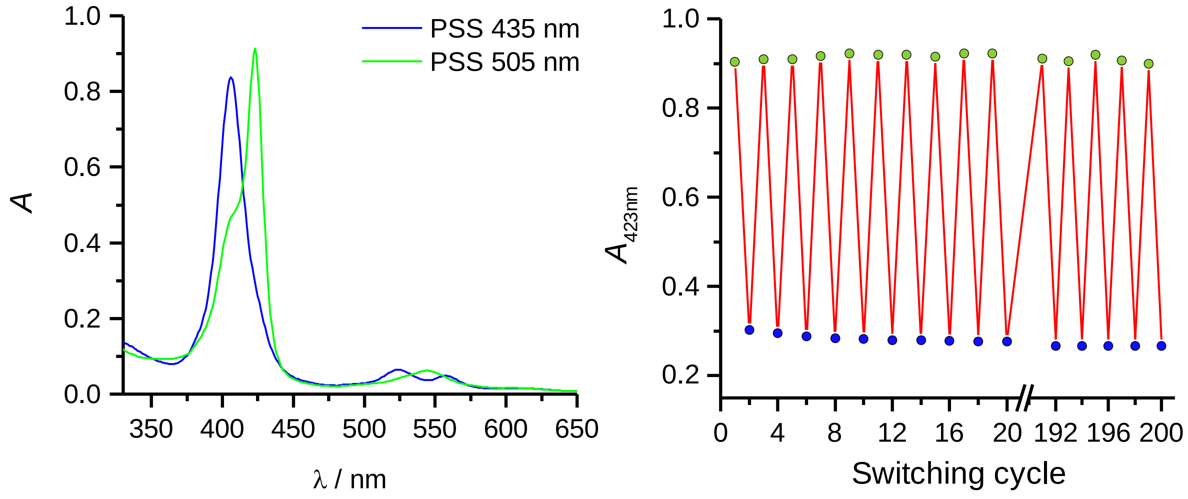

Figure S18.8: UV–vis spectra in the PSS at 505 nm and 435 nm of **1j** (left) and absorbance at 423 nm ($\lambda_{max.}$ paramagnetic Soret peak) of porphyrin **1j** in acetone at 25 °C after irradiation with 505 nm (green) and 435 nm (blue) plotted against the number of switching cycles (right). No degradation was observed within 200 switching processes.

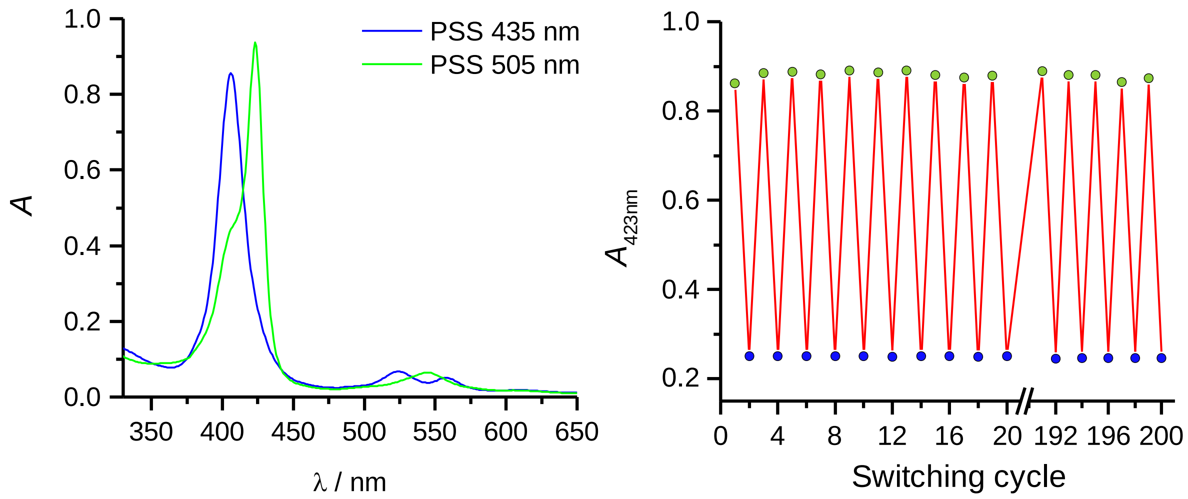

Figure S18.9: UV–vis spectra in the PSS at 505 nm and 435 nm of **1j** (left) and absorbance at 423 nm ($\lambda_{max.}$ paramagnetic Soret peak) of porphyrin **1j** in acetone and addition of Cs_2CO_3 at 25 °C after irradiation with 505 nm (green) and 435 nm (blue) plotted against the number of switching cycles (right). No degradation was observed within 200 switching processes.

Spin switching in an aqueous environment

A solution of record player **1e**, **1h**, **1j** in acetone was diluted with oxygen free water and acetone to obtain a ratio of 1:9 for acetone:water. UV–vis spectra in the PSS at 505 nm and 435 nm were recorded. Afterwards an excess of Cs_2CO_3 was added for deprotonation and UV/vis spectra in the PSS were recorded.

Hammett Plot

The equilibrium constant (K_1) in the PSS at 505 nm (Table S3) for the coordination of the axial ligand (CN4 → CN5) was determined from ^{1}H NMR spectroscopy (Equation S3), which is a powerful tool to obtain the ratio of coordinated/decordinated *cis* isomer (Equation S1).

$$\boldsymbol{K_1} = \frac{\boldsymbol{c^*(\mathrm{CN5})}}{\boldsymbol{c^*(\mathrm{CN4})}} = \frac{\dfrac{\boldsymbol{\delta_{obs.}} - \boldsymbol{\delta_{dia.}}}{\boldsymbol{\delta_{max.}} - \boldsymbol{\delta_{dia.}}}}{\mathbf{1} - \left(\dfrac{\boldsymbol{\delta_{obs.}} - \boldsymbol{\delta_{dia.}}}{\boldsymbol{\delta_{max.}} - \boldsymbol{\delta_{dia.}}}\right)} \quad \text{(Equation S3).}$$

K_1	equilibrium constant for the coordination/decoordination of the axial ligand (CN4 → CN5).
*c**(CN5)	relative concentration of the fivefold coordinated complex.
*c**(CN4)	relative concentration of the fourfold coordinated complex.
$\delta_{obs.}$	observed average shift of the *cis* pyrrole protons in ppm.
$\delta_{dia.}$	shift of the completely diamagnetic complex (determined by TFA-d_1 addition) in ppm.
$\delta_{max.}$	maximum shift (100 % coordinated) for a fivefold coordinated complex determined by Gutzeit et al. [6] in ppm.

The logarithm ($\log_{10}$) of K_{1x}/K_H as a function of the Hammett parameter σ_{para} of the 4-substituent of the axial ligand gave the Hammett plot (Figure 2).

Table S3: Relative concentration of intramolecular coordinated (*c**(CN5)) and decoordinated (*c**(CN4) **1a–j** in the PSS at 505 nm and the determined equilibrium constant (K_1) for the coordination (CN4 → CN5) and the logarithm ($\log_{10}$ (K_{1x}/K_H)).

Molecule	*c**(CN5)	*c**(CN4)	K_1	$\log_{10}$ (K_{1x}/K_H)
**1a**	83	17	4.9	0
**1b**	97	3	32.3	0.81
**1c**	99	1	99.0	1.31
**1d**	76	24	3.2	-0.19
**1e**	95	5	19.0	0.59
**1e**[d]	99	1	99.0	1.31
**1f**	96	4	24.0	0.69
**1h**	99	1	99.0	1.31

**1h**[a]	95	5	19.0	0.59
**1i**	39	61	0.64	-0.89
**1j**	99	1	99.0	1.31
**1j**[d]	99	1	99.0	1.31

[a]Deprotonated with Cs_2CO_3

IV: References

1. Fulmer, G. R.; Miller, A. J. M.; Sherden, N. H.; Gottlieb, H. E.; Nudelman, A.; Stoltz, B. M.; Bercaw, J. E.; Goldberg, K. I. *Organometallics* **2010,** *29,* 2176–2179. DOI: 10.1021/om100106e

2. Yasukawa, N.; Kuwata, M.; Imai, T.; Monguchi, Y.; Sajiki, H.; Sawama, Y. *Green Chem.* **2018,** *20,* 4409–4413. DOI: 10.1039/C8GC01373J

3. Tibiletti, F.; Simonetti, M.; Nicholas, K. M.; Palmisano, G.; Parravicini, M.; Imbesi, F.; Tollari, S.; Penoni, A. *Tetrahedron* **2010,** *66,* 1280-1288. DOI: 10.1016/j.tet.2009.12.020

4. Dommaschk, M.; Peters, M.; Gutzeit, F.; Schütt, C.; Näther, C.; Sönnichsen, F. D.; Tiwari, S.; Riedel, C.; Boretius, S.; Herges, R. *J. Am. Chem. Soc.* **2015**, *137,* 7552–7555. DOI: 10.1021/jacs.5b00929

5. Dommaschk, M.; Schütt, C.; Venkataramani, S.; Jana, U.; Näther, C.; Sönnichsen, F. D.; Herges, R. *Dalton Trans.* **2014**, *43*, 17395-17405. DOI: 10.1039/C4DT03048F

6. Gutzeit, F.; Dommaschk, M.; Levin, N.; Buchholz, A.; Schaub, E.; Plass, W.; Näther, C.; Herges, R. *Inorg. Chem.* **2019**, 12542–12546. DOI: 10.1021/acs.inorgchem.9b00348

7. Dommaschk, M.; Gröbner, J.; Wellm, V.; Hövener, J.-B.; Riedel, C.; Herges, R. *Phys. Chem. Chem. Phys.* **2019**, 24296-24299. DOI: 10.1039/C9CP04156G

8. TURBOMOLE V7.2 2017, a development of University of Karlsruhe and Forschungszentrum Karlsruhe GmbH, 1989-2007, TURBOMOLE GmbH, since 2007; available from http://www.turbomole.com.

9. Perdew, J. P.; Burke, K.; Ernzerhof, M. *Phys. Rev. Lett.* **1996**, *77*, 3865–3868. DOI: 10.1103/PhysRevLett.77.3865

10. Weigend, F.; Ahlrichs, R. *Phys. Chem. Chem. Phys.* **2005**, *7*, 3297–3305. DOI: 10.1039/B508541A

11. Becke, A. D. *J. Chem. Phys.* **1993**, *98,* 5648–5652. DOI: 10.1063/1.464913
